# Quantitative Differences in Nuclear β-catenin and TCF Pattern Embryonic Cells in *C*. *elegans*

**DOI:** 10.1371/journal.pgen.1005585

**Published:** 2015-10-21

**Authors:** Amanda L. Zacharias, Travis Walton, Elicia Preston, John Isaac Murray

**Affiliations:** Department of Genetics, Perelman School of Medicine, University of Pennsylvania, Philadelphia, Pennsylvania, United States of America; California Institute of Technology, UNITED STATES

## Abstract

The Wnt signaling pathway plays a conserved role during animal development in transcriptional regulation of distinct targets in different developmental contexts but it remains unclear whether quantitative differences in the nuclear localization of effector proteins TCF and β-catenin contribute to context-specific regulation. We investigated this question in *Caenorhabditis elegans* embryos by quantifying nuclear localization of fluorescently tagged SYS-1/β-catenin and POP-1/TCF and expression of Wnt ligands at cellular resolution by time-lapse microscopy and automated lineage tracing. We identified reproducible, quantitative differences that generate a subset of Wnt-signaled cells with a significantly higher nuclear concentration of the TCF/β-catenin activating complex. Specifically, β-catenin and TCF are preferentially enriched in nuclei of daughter cells whose parents also had high nuclear levels of that protein, a pattern that could influence developmental gene expression. Consistent with this, we found that expression of synthetic reporters of POP-1-dependent activation is biased towards cells that had high nuclear SYS-1 in consecutive divisions. We identified new genes whose embryonic expression patterns depend on *pop-1*. Most of these require POP-1 for either transcriptional activation or repression, and targets requiring POP-1 for activation are more likely to be expressed in the cells with high nuclear SYS-1 in consecutive divisions than those requiring POP-1 for repression. Taken together, these results indicate that SYS-1 and POP-1 levels are influenced by the parent cell’s SYS-1/POP-1 levels and this may provide an additional mechanism by which POP-1 regulates distinct targets in different developmental contexts.

## Author Summary

The Wnt signaling pathway is active during the development of all multi-cellular animals and also improperly re-activated in many cancers. Here, we use time-lapse microscopy to quantify the nuclear localization of several proteins in response to Wnt signaling throughout early embryonic development in the nematode worm, *C*. *elegans*. We find that cells that received a Wnt signal in the previous division respond more strongly to a Wnt signal in the next division, in part by localizing more of the regulator β-catenin to the nucleus. This causes the relative enrichment of Wnt pathway proteins in the nuclei of repeatedly signaled cells, which we show likely impacts the activation of Wnt target genes. This represents a novel mechanism for the regulation of Wnt pathway targets in development and disease.

## Introduction

The Wnt pathway is a conserved cell-cell signaling pathway repeatedly utilized during development and homeostasis in all metazoans. Wnt plays a prominent conserved role in many developmental processes including patterning of anterior-posterior (A-P) body axes [1] and stem cell self-renewal [2]. Wnt ligands are expressed posteriorly to establish gradients that pattern the A-P axis in diverse contexts including planarian regeneration [3], neurectoderm patterning in frogs [4], and primary axis specification [5], neural crest diversification [6], and limb patterning in mice [7]. Wnt can act both as a long-range diffusible morphogen and through direct cell contact to regulate gene expression and to orient cell divisions [8–11]. In all systems examined, the same signal can activate the expression of different target genes depending on cell type and developmental stage [12]. This context-dependent activity results in part from the activity of distinct partner transcription factors that interact with the Wnt pathway to regulate target genes [13,14]. In other signaling pathways, the strength of pathway activation, through increased or temporally extended ligand-receptor interactions, affects the target genes activated (i.e. morphogen), often with a gradient of repressor acting in opposition to the activator [15–17]. However it remains unclear whether Wnt can act as a morphogen [18] or whether quantitative differences in Wnt pathway activity, as inferred by the nuclear localization of β-catenin, exist *in vivo* and contribute to context-specific regulation.

The *C*. *elegans* embryo is an ideal system for quantitative analysis of Wnt pathway-mediated regulation because of the broad role of the pathway in patterning most embryonic divisions [14,19] and the embryo’s known invariant lineage [20] and transparency. While other well-known signaling pathways each regulate a few important cell fate decisions in the worm (e.g. [21–24]), Wnt acts recursively across most divisions to orient them along the A-P axis and ensure high nuclear β-catenin and appropriate fate in each posterior daughter [14,19,25]. Similar binary patterning occurs in the annelid *Platynereis dumerilii* and the ascidian *Ciona intestinalis*, suggesting it may reflect an ancestral role for Wnts in diversifying cell fates [26,27].

**Fig 1:**
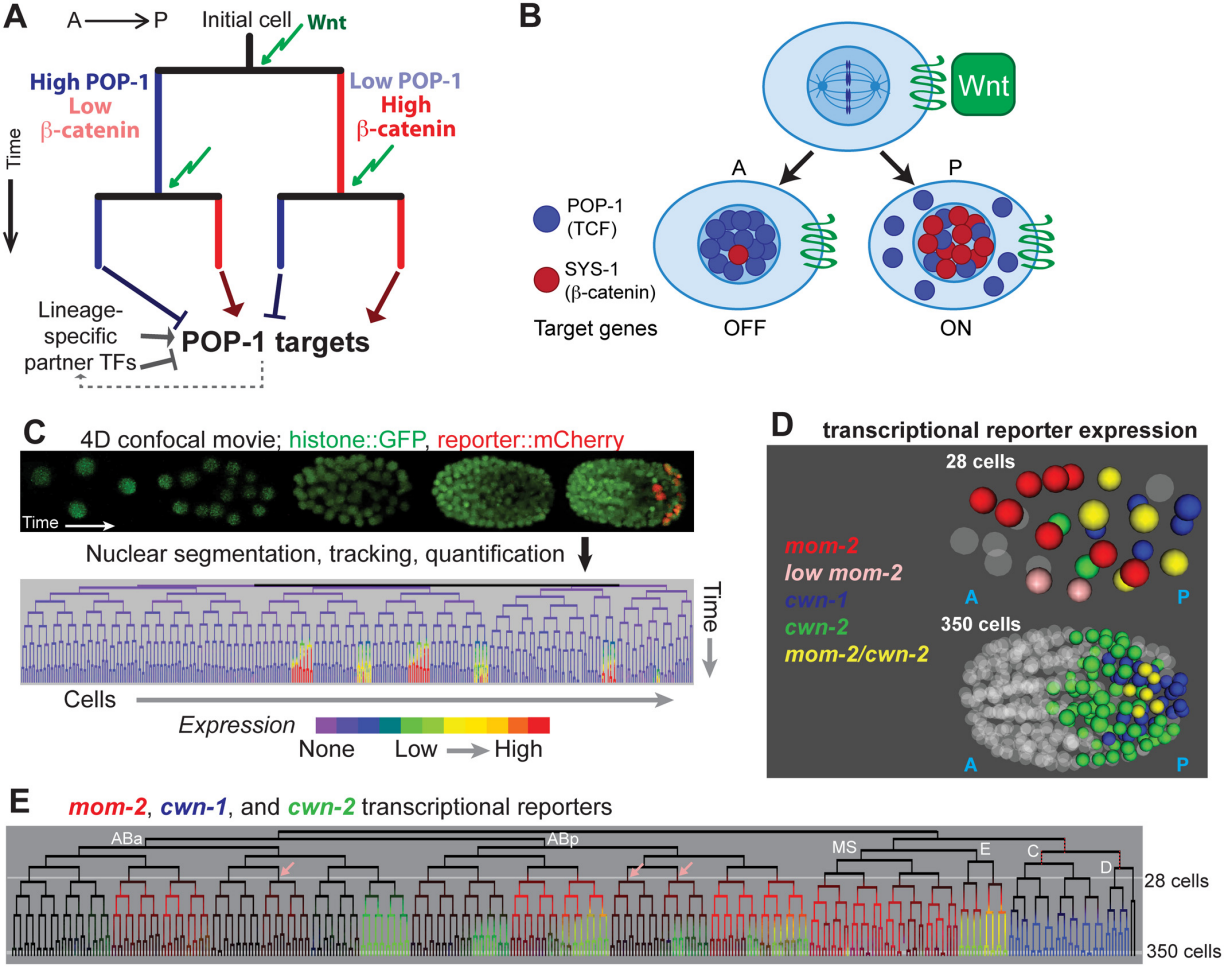
Wnt signaling and sources of Wnt ligand in the early *C*. *elegans* embryo. A) Existing model for Wnt-mediated expression. Wnt (green arrow) induces posterior daughters to have high nuclear beta-catenin (red) and low nuclear POP-1 (blue), and the converse in anterior daughters. This results in activation of POP-1 targets in posterior daughters and repression in anterior daughters. The process is repeated in each subsequent division. Since POP-1 targets differ between cells, other factors, possibly themselves POP-1 targets, must provide context information. B) Stoichiometry model of POP-1/SYS-1 target expression regulation. Wnt ligand orients the division axis of a cell and generates an anterior daughter with high nuclear POP-1 and low SYS-1 and a posterior daughter with high nuclear SYS-1 and low POP-1. C) In automated lineage tracing, movies of embryos expressing fluorescent histones (*Pnob-1* shown) is processed by StarryNite cell tracking software [47, 80] to produce a lineage tree with expression quantified at each time point. D) Location of the nuclei expressing the 3 earliest Wnt ligands at the 28 and 350 cell stages. E) Transcriptional reporter expression of the earliest three Wnt ligands: *mom-2* (red), *cwn-1* (blue) and *cwn-2* (green). Lineages expressing low levels of *mom-2* are marked with pink arrows. Outside of the E lineage, there is little overlap (yellow) suggesting *mom-2* expression is down-regulated as *cwn-2* is activated in co-expressing lineages. Known maternal expression of *mom-2* is indicated by red dotted lines [25].

Most *C*. *elegans* embryonic divisions are patterned by the Wnt/β-catenin asymmetry pathway [28], a variant of “canonical” Wnt signaling found in both worms and humans in which signaling leads to both nuclear localization of β-catenin and nuclear export of some, but not all, of the Wnt-effector transcription factor POP-1/TCF [14,25,29–31]. Exposure of a dividing cell to Wnt orients the spindle such that the daughters are positioned proximal and distal to the signaling cell (Fig 1B) [25,32]. Subsequently, the β-catenin expressed in embryos, SYS-1, and the β-catenin related protein, WRM-1, are preferentially localized to the nucleus of posterior-born daughters of most divisions [33–35]. Nuclear WRM-1 partners with the Nemo-Like Kinase, LIT-1, to phosphorylate some POP-1 protein, triggering its nuclear export [30,36,37]. As a result, the anterior daughter nucleus has higher POP-1 and lower β-catenin relative to the posterior nucleus (Fig 1A and 1B). Most early divisions prior to the 16-cell stage require the Wnt ligand *mom-2* for asymmetric localization of POP-1 and β-catenin [31]. Curiously, POP-1 asymmetry in later divisions appears to require neither *mom-2* nor a neighboring inducing cell, although both are needed for proper division orientation [25,38].

POP-1/SYS-1 target regulation is thought to depend on the stoichiometry of POP-1 and SYS-1 [31]. Nuclear export of POP-1 likely ensures that all remaining nuclear POP-1 is associated with SYS-1 [39]; in posterior daughters, POP-1 can bind targets as a complex with SYS-1 and activate expression, while in anterior daughters, POP-1 binding in the absence of SYS-1 leads to repression [36]. The differential nuclear localization of POP-1 and SYS-1 can thus regulate distinct target gene expression between sister cells [28,31]. Targets are differentially regulated in different divisions, even consecutive divisions separated by as little as 15 minutes [40], in part due to unique expression of context-specific co-regulators (Fig 1A, e.g. [14,41,42]).

Previous studies suggested a binary model for Wnt activity (Fig 1A) but it is not known whether quantitative variability in nuclear POP-1/SYS-1 localization influences context-specific target regulation. We addressed this question by assessing the expression and regulation of Wnt pathway components and targets across all embryonic cells through morphogenesis by using automated lineage-tracing methods. We identified the cells that express Wnt ligands and quantified the nuclear localization of β-catenin and POP-1 in each cell throughout development. We identified reproducible quantitative variation in nuclear β-catenin and POP-1. “Double-posterior” daughter nuclei that were the posterior daughter in two successive divisions had higher β-catenin than “single-posterior” nuclei whose parents had low nuclear β-catenin and the reverse was true for POP-1 in “double-anterior” nuclei, irrespective of position in the embryo. Synthetic TCF activity reporters are preferentially expressed in the cells where this cousin enrichment leads to the highest SYS-1:POP-1 ratio. We identify new genes with *pop-1*-dependent expression and find that genes that require *pop-1* for activation are preferentially expressed in lineages derived from the cells with higher nuclear β-catenin compared with repressed targets. Taken together, our results indicate that *C*. *elegans* embryonic cells integrate the nuclear localization of POP-1/SYS-1 across multiple divisions, and suggest that the resulting activity differences diversify gene expression in embryonic progenitor cells.

## Results

### Wnt ligands are dynamically expressed from the posterior of the embryo

Previous studies demonstrated that the five *C*. *elegans* Wnt ligands, *mom-2*, *cwn-1*, *cwn-2*, *egl-20* and *lin-44*, are expressed at distinct times in medial and posterior embryonic positions, but did not determine which cells express each Wnt [43–46]. We hypothesized that cells that express Wnt ligand might have increased nuclear localization of SYS-1. In order to understand whether exposure to Wnt ligand quantitatively affects the nuclear localization of POP-1/SYS-1, it is necessary to know when and where the Wnt ligands are expressed. We identified all cells expressing transcriptional reporters for each Wnt ligand at single cell resolution throughout embryonic cleavage by 4D imaging and automated cell tracking and reporter quantification (“lineage analysis”, Fig 1C, described in Methods) [47–51].

*mom-2* is expressed maternally in descendants of the P_1_ and P_2_ blastomeres [44,52], and we observed transient zygotic activity of the *mom-2* promoter in MS, E and several posterior AB sublineages. The *cwn-2* promoter is activated in a partially overlapping set of AB sublineages (Fig 1E) and *cwn-1* promoter expression is limited to the C and D lineages [53,54]. Reporters for *egl-20*, *lin-44* and *mom-2* are expressed much later, just prior to the final round of embryonic divisions, in a handful of cells in the tail (S1 Fig). These patterns are consistent with these genes’ endogenous mRNA expression patterns as measured by FISH [44], and provide the first cellular resolution map of Wnt ligand transcription across the embryo.

### Asymmetric nuclear β-catenin localization occurs after most embryonic divisions

Previous work indicated that the Wnt pathway is preferentially active in the posterior daughters of most embryonic divisions [14,19,25,28,35]. However, given the posterior bias in Wnt ligand expression, we investigated whether nuclear β-catenin localization was quantitatively biased towards the posterior, as it is the major mechanism by which Wnt ligand regulates transcription [28,31,34,35]. Two *C*. *elegans* β-catenin homologs influence embryonic expression; SYS-1 binds to POP-1 and converts it to an activator [28,31] and WRM-1 promotes nuclear export of excess POP-1 that would otherwise repress target transcription [30]. The asymmetric localization of these β-catenins to the nuclei of posterior daughter cells is well established, but nuclear concentrations have not been quantified or compared between cells across developmental time [28,31].

**Fig 2:**
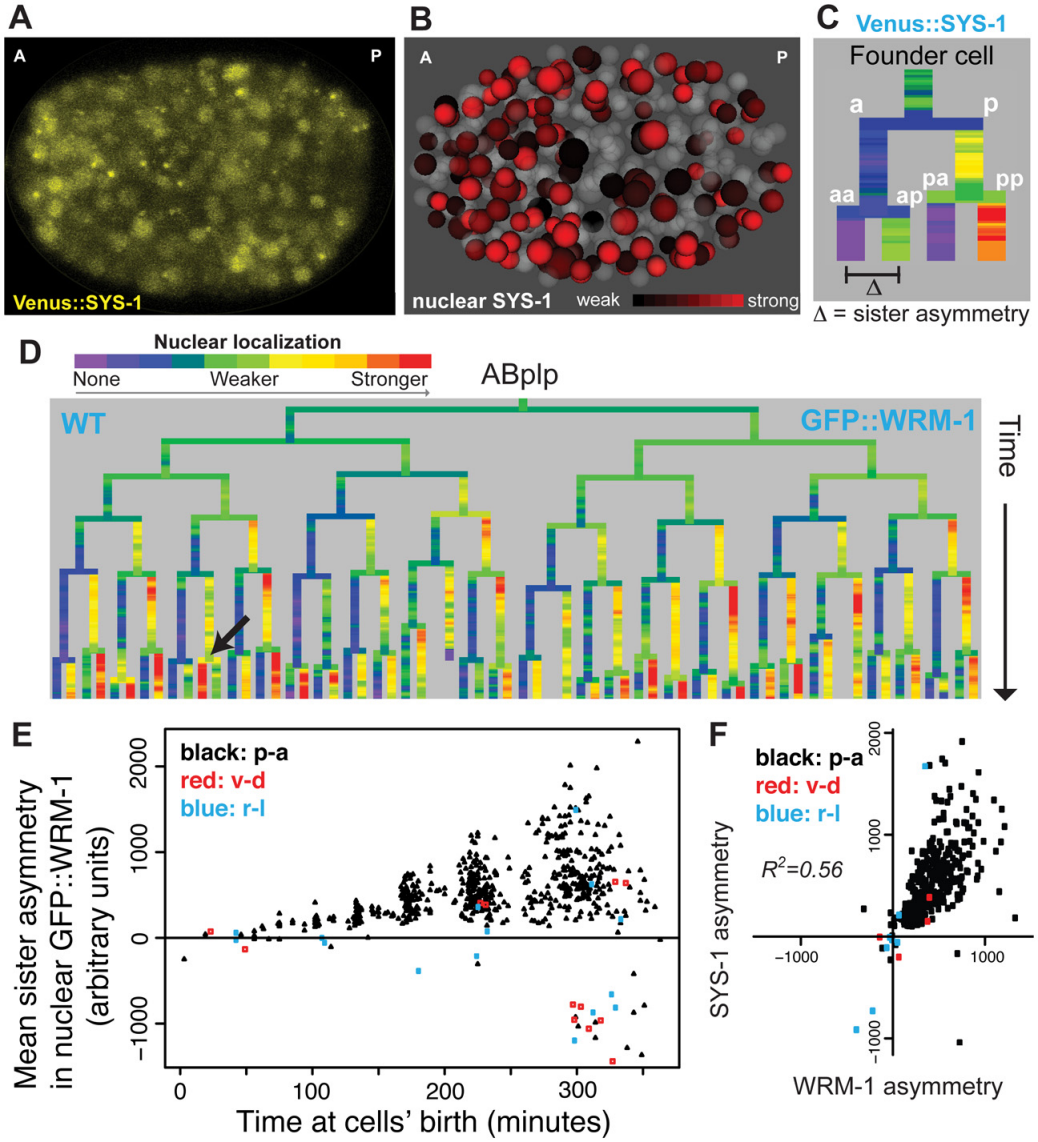
Nuclear localization of β-catenins is asymmetric across most embryonic cells. A) Confocal image of Venus::SYS-1 in a 350 cell stage embryo. B) Quantification of nuclear localization for Venus::SYS-1 shows that cells with strong nuclear localization of β-catenin (red) are distributed across the A-P axis. Grey cells have little to no detectable nuclear Venus::SYS-1. C) Overview of naming rules. Posterior daughter cells are represented as right branches on lineage trees. Sister asymmetry is measured by subtracting expression between daughter cells. D) Mean nuclear GFP::WRM-1 levels across the ABplp lineage. Branch color represents nuclear fluorescence intensity on a scale from purple (weak nuclear depletion) to green (weak nuclear enrichment) to red (strong nuclear enrichment). Note stronger enrichment in posterior daughters of later divisions and rare divisions with reversed polarity (black arrow). E) Mean sister asymmetry in nuclear GFP::WRM-1 for each division assayed through bean stage (~600 cells). Colors denote orientation of division according to Sulston. F) Correlation between mean GFP::WRM-1 and Venus::SYS-1 asymmetry for each division through the 350-cell stage.

We measured levels (quantified as the total fluorescent intensity in 3D for each nucleus) and concentrations (mean 3D pixel intensity) of rescuing GFP::WRM-1 and Venus::SYS-1 fluorescent reporters in all nuclei relative to the surrounding cytoplasm through the 600-cell stage by lineage analysis (Fig 2A–2C, S2 Fig). As expected, nearly every embryonic division has asymmetric nuclear β-catenin levels between its daughter cells (“sister asymmetry”). 91% of observed embryonic divisions had significant sister asymmetry (542/594; nominal p<0.05; false discovery rate < 6%; S3 Table,) and nearly all other divisions had reproducible sister asymmetry between replicates. Consistent with the demonstrated role of Wnt in defining posterior daughter identity, β-catenin levels were higher in the posterior daughter for over 98% of A-P oriented divisions with significant asymmetry (Fig 2D). However we identified ten late divisions that consistently escape this rule and have reversed polarity with higher levels in the anterior daughter, indicating unexpected complexity in these cells’ polarization (S3 Table, S3 Fig). Finally, we observed significant β-catenin sister asymmetry for 19 of 26 divisions that are oriented on the L-R and D-V axes. Many of these divisions occurred near the anterior or posterior embryonic pole, and the daughter with higher nuclear β-catenin was either closer to the posterior pole or farther from the anterior pole. The location of putative signaling centers can be identified based on these patterns of β-catenin asymmetry (S4 Fig), suggesting Wnt distinguishes A-P across the whole embryo and also medial-lateral axes at the poles. Together, this dataset defines the orientation and strength of nuclear β-catenin polarization for most *C*. *elegans* embryonic divisions and is consistent with the known widespread use of this pathway to diversify embryonic cell fates.

### Nuclear β-catenin is quantitatively enriched over successive cell cycles

We next asked whether there is reproducible quantitative variation in β-catenin localization between cells. GFP::WRM-1 [35] and Venus::SYS-1 [28] had reproducible nuclear concentrations between replicate embryos (mean r = 0.91 for WRM-1, 0.93 for SYS-1) and similar patterns between WRM-1 and SYS-1 (Fig 2F, r = 0.56). Nuclear β-catenin concentrations increase as nuclei become smaller over time but total amounts per nucleus were roughly constant (Fig 2E, S5 Fig). Despite the posterior expression of Wnt ligands, β-catenin sister asymmetry is more pronounced in anterior regions of the embryo compared with posterior regions (S4 Fig). Furthermore, cells with high levels of nuclear β-catenin are found throughout the anterior-posterior axis (Fig 2B), suggesting that higher ligand expression does not necessarily lead to higher nuclear β-catenin in neighboring cells. When we compared the daughters of Wnt ligand expressing cells to those not expressing ligand, we again found that the daughters of ligand expressing cells had lower β-catenin sister asymmetry (30% lower, p<10^−6^, Wilcoxon rank sum test).

**Fig 3:**
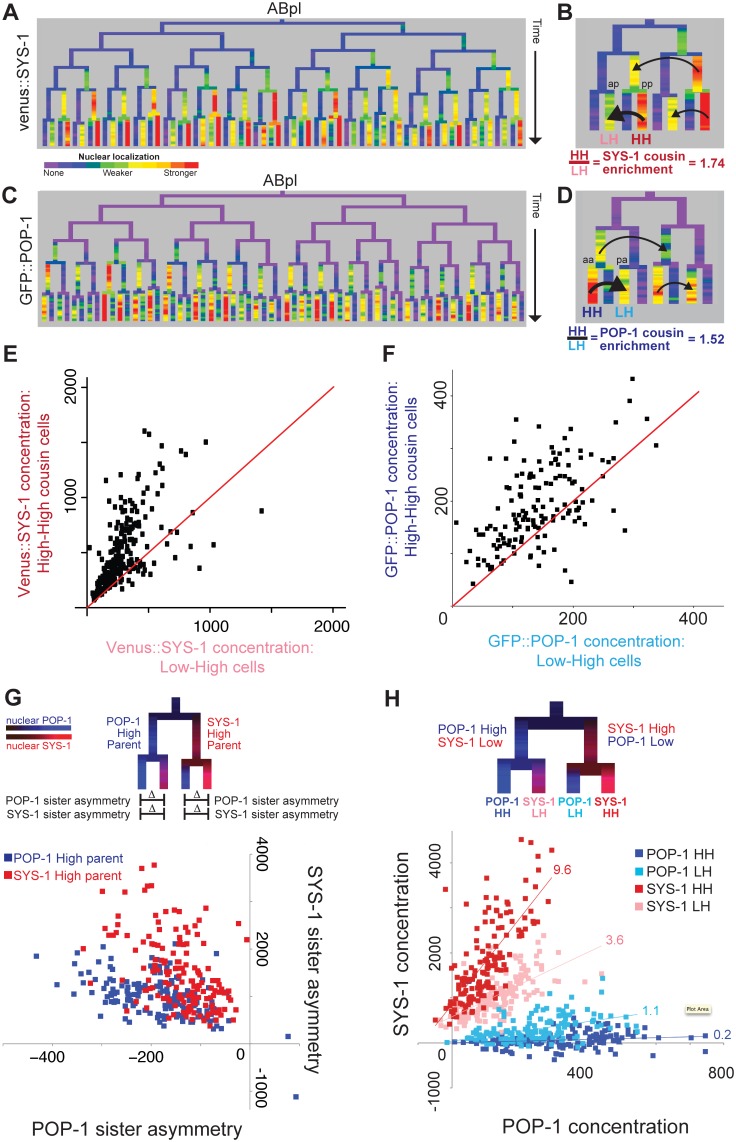
β-catenin and TCF show enrichment when localized to the nucleus in successive cell cycles. A) Nuclear localization of Venus::SYS-1 in the ABpl lineage, shows the highest level in cells derived from consecutive posterior parents. B) Detail showing ABplaaa lineage; Black arrows show comparison between posterior cousin cells with high nuclear Venus::SYS-1 in two consecutive divisions (High-High or HH cells, ABplaaaapp marked “pp”), which have higher levels of nuclear Venus::SYS-1 than their cousins with parents that had no/low nuclear Venus::SYS-1 (Low-High or LH cells, ABplaaaaap marked “ap). C) Nuclear localization of GFP::POP-1, detectable after the 50 cell stage. GFP::POP-1 polarity is the opposite of Venus::SYS-1 with highest expression in cells derived from consecutive anterior parents. D) Detail of ABplppp lineage for GFP::POP-1 showing comparison between anterior cousin cells with high nuclear POP-1 in consecutive divisions (ABplpppaa marked “aa”) and their cousins with parents that had low nuclear POP-1 (ABplppppa marked “pa”). E) Venus::SYS-1 concentration for embryonic High-High cells (y axis) compared with their Low-High cousin cell. Most points fall above the red y = x line. F) Average GFP::POP-1 concentration for all High-High cells (y-axis) born after the 50 cell stage compared with their Low-High cousin cells. Most points fall above the red y = x line. G) Diagram illustrating comparison of SYS-1 and POP-1 asymmetry levels in daughter cells and graph showing POP-1 and SYS-1 asymmetry levels for all divisions after the 50-cell stage. Divisions of parent cells with high levels of POP-1 (blue) tend to have greater POP-1 asymmetry (x-axis) and less SYS-1 asymmetry (y-axis) than parent cells with high levels of SYS-1(red). H) Low/High labeling scheme and graph showing comparison of concentrations of Venus::SYS-1 and GFP::POP-1 for all individual cells after the 50-cell stage, colored by whether they are POP-1 High-High (dark blue), POP-1 Low-High (light blue), SYS-1 High-High (red) or SYS-1 Low-High (pink). The relative ratio of Venus::SYS-1 fluorescence intensity to GFP::POP-1 intensity for each group can be estimated from slope of a best-fit line (indicated above each line). Concentration values can be negative because nuclear intensity is subtracted from local background.

Strikingly, we found that both nuclear β-catenin concentration and sister asymmetry are significantly higher in posterior daughters of cells that were themselves posterior daughters (Fig 3A and 3E). These β-catenin “High-High” cells are defined by high nuclear β-catenin over two successive cell cycles. The “High-High” cells have significantly higher nuclear β-catenin than their “Low-High” cousin cells whose mother had low nuclear β-catenin (Fig 3B and 3E) ([SYS-1] 74% higher; [WRM-1] 24% higher; combined p<10^−5^). This effect is present at all time points and for all nuclear sizes. Furthermore, the low nuclear Venus::SYS-1 in anterior daughters was further decreased if the mother also had low Venus::SYS-1 (Fig 3H, S4 Fig). We also examined an mCherry-tagged version of SYS-1 (S2 Fig) and found it was enriched 160% in “High-High” cells over their “Low-High” cousins. This suggests that the level of β-catenin localization varies quantitatively, even across cells with high nuclear β-catenin. For shorthand, we refer to the transmitotic accumulation of additional nuclear β-catenin in the “High-High” daughter cells over their “Low-High” cousins as the “cousin enrichment” of nuclear β-catenin. We emphasize that the anterior-posterior naming rules reflect local differences between daughter cells, so the “High-High” cells are distributed throughout the embryo, including many in the physical anterior (e.g. brighter red cells in Fig 2B).

We asked whether this cousin enrichment of nuclear β-catenin compounds over more than two cell cycles by comparing concentration between triple-posterior (“High-High-High”) cells and double-posterior “Low-High-High” cells. We saw a small but significant further increase in concentration in triple-posterior cells compared with double-posterior cells for SYS-1 (18%, p = 0.02) but not WRM-1, suggesting that cousin enrichment of nuclear β-catenin is strongest after one cell division but may be maintained through a second division. Cousin enrichment is not solely due to preferential inheritance of β-catenin by posterior daughter cells, as we find that “Low-High-High” double-posterior cells show higher concentration than the single-posterior “High-Low-High” cells with the same total number of posterior divisions (SYS-1 63% higher, p<10^−12^; WRM-1 47% higher, p<10^−5^).

### POP-1 shows cousin enrichment in double-anterior nuclei

Since the ratio of POP-1 to SYS-1 in each nucleus is thought to determine the transcriptional response [31], we measured nuclear GFP::POP-1 levels by lineage analysis. We analyzed GFP::POP-1 expressed from a nearly uniform promoter beginning at the 50 cell stage (Fig 3C, 3D and 3F and S2 Fig) [55]; a strain expressing a pulse of GFP::POP-1 in the EMS lineage from a *med-1* promoter [40] gave similar results (S6 Fig). As previously reported [14,25], GFP::POP-1 levels were higher the anterior daughters of A-P divisions, which we observed for 96% of divisions between the 50 cell stage and the 350 cell stage (nominal p<0.05, FDR <6%), and polarity was reversed for the same divisions that we identified as having reversed β-catenin asymmetry (S3 Table). POP-1 sister asymmetry was also lower in cells with parents that expressed Wnt ligand compared to non-expressing parents (30% lower, p<10^−11^, Wilcoxon rank sum test).

We found that nuclear POP-1 levels mirrored nuclear SYS-1 levels with significantly higher nuclear GFP::POP-1 concentrations in cells that are the products of consecutive anterior divisions (“POP-1 High-High” cells) compared with their single-anterior cousins (“POP-1 Low-High” cells) (52% increase, p<10^−4^; Fig 3D and 3F) indicating that TCF has cousin enrichment inverse to that of β-catenin. We verified that this enrichment also occurs for endogenous POP-1 protein by immunofluorescence quantification of POP-1 in wildtype embryos. We examined 60 high-high cells and found significant cousin enrichment (p<10^−15^) and similar levels of enrichment in the cells examined (31% with antibody vs. 27% with GFP::POP-1, S6 Fig). For GFP::POP-1, we also saw a smaller but significant increase for triple-anterior POP-1 High-High-High divisions as compared to Low-High-High (23%, p = 0.0009), similar to that observed for SYS-1. As a result, the asymmetry biases of POP-1 and SYS-1 are in opposition to each other (Fig 3G and 3H). Divisions of parent cells with high nuclear POP-1 give high POP-1 asymmetry and moderate SYS-1 asymmetry, while divisions of parent cells with high nuclear SYS-1 give high SYS-1 asymmetry and moderate POP-1 asymmetry (Fig 3G). The resulting quantitative differences in the ratio of Venus::SYS-1 fluorescence intensity to GFP::POP-1 intensity for each group can be estimated from slope of a best-fit line (Fig 3H). We find that the SYS-1 High-High cells have on average 48-fold more SYS-1 per unit POP-1 than the POP-1 High-High cells; SYS-1 Low-High cells and POP-1 Low-High cells have 18-fold and 5.5-fold more, respectively. These findings suggest POP-1 may be most potent as a transcriptional activator in the double-posterior SYS-1 High-High cells and as a transcriptional repressor in the double-anterior POP-1 High-High cells.

### Frizzled receptor and Wnt ligands are essential for full nuclear β-catenin sister asymmetry but only Frizzled affects cousin enrichment

**Fig 4:**
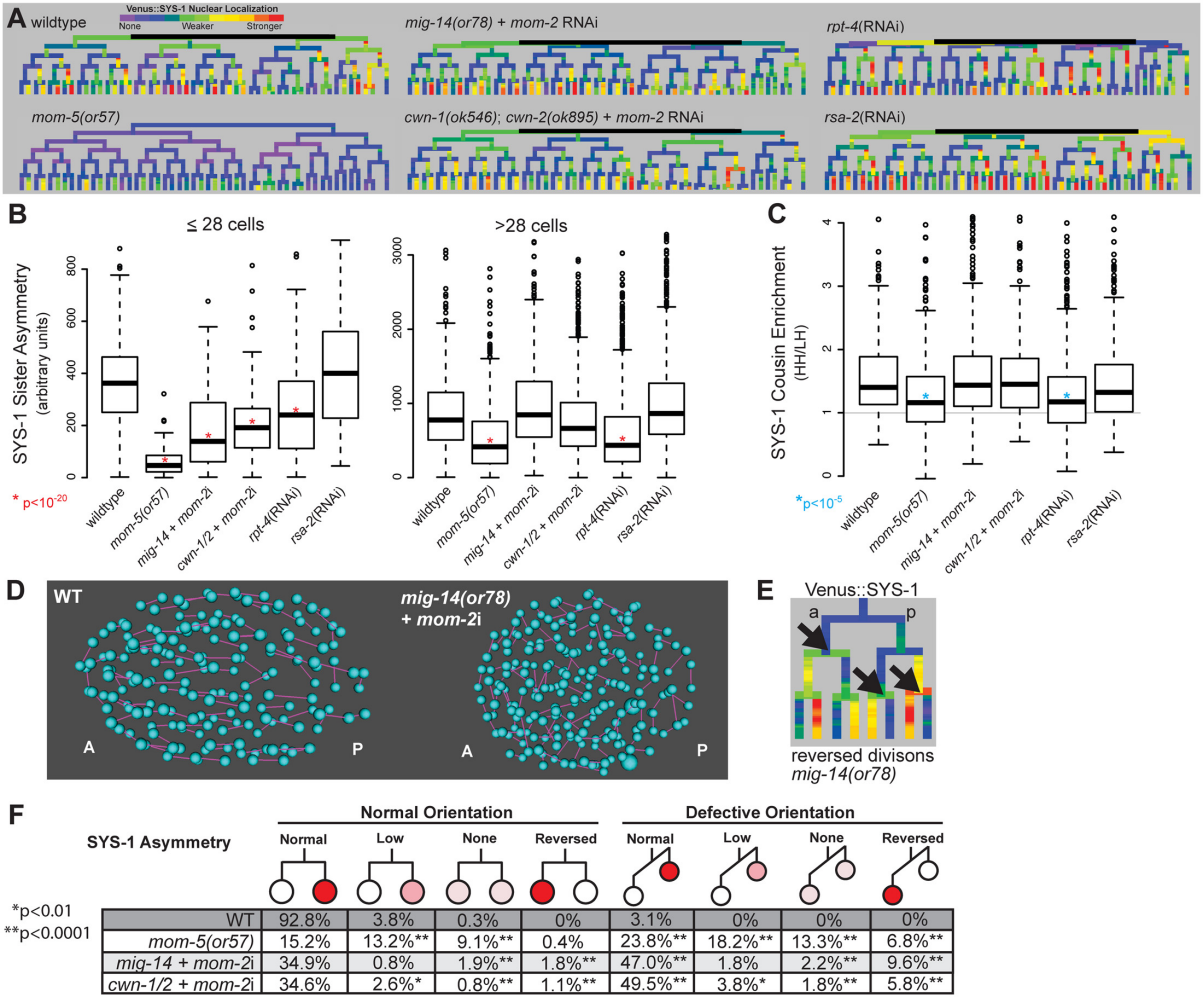
Reduction in Wnt receptor or ligand disrupts nuclear β-catenin asymmetry, cousin enrichment, and division orientations. A) Lineage trees showing examples of Venus::SYS-1 nuclear localization through the 51 cell stage show that in *mom-5(or57)* frizzled receptor mutants SYS-1 is completely excluded from the nucleus in virtually all early divisions, while mutations that disrupt Wnt ligand, *mig-14(or78)* and *cwn-1(ok546);cwn-2(ok895)* that were treated with RNAi against *mom-2*, show some divisions with reduced or reversed asymmetry. RNAi against the proteasomal subunit *rpt-4* decreases nuclear SYS-1 in some lineages, while RNAi against the centrosome component *rsa-2* increases it. B) Boxplots show SYS-1 sister asymmetry is dramatically reduced in early *C*. *elegans* Wnt receptor and ligand mutant embryos as compared to wild type (p<10^−20^, *) and reduced only in *mom-5(or57)* mutant and *rpt-4* RNAi-treated embryos after the 28 cell stage. SYS-1 sister asymmetry is unaffected by *rsa-2* RNAi. Edges of the boxes represent the upper and lower quartile, the heavy center line represents the median, the whiskers show the upper and lower bounds, and outliers are shown as circles. C) SYS-1 cousin enrichment (see Fig 3B) is disrupted only in the *mom-5(or57)* mutant and *rpt-4* RNAi-treated embryos. Gray line indicates the SYS-1 cousin enrichment value at which SYS-1 High-High and Low-High cousin nuclear localization are equal (1). D) Division orientations (purple lines) between daughter cells (blue) in wild-type and Wnt ligand mutant embryos at mid-embryogenesis. E) In divisions with reversed polarity (black arrows), the anterior (left) cell has more nuclear SYS-1 than the posterior (right) cell. F) Breakdown of the divisions that have normal or defective orientations and normal, low, none, or reversed SYS-1 asymmetry in wild-type (WT) and Wnt ligand mutant embryos. Total numbers of defective division orientations, cells with reversed polarity and no polarity are significantly different (p<0.0001, **) from wild-type in all three mutant conditions. Divisions with low polarity are also significantly increased for *mom-5(or57)* and *cwn-1/2* + *mom-2*i.

The maternally expressed Wnt ligand, *mom-2* is required for SYS-1/POP-1 asymmetry in the early divisions prior to the 28-cell stage (AB16), but asymmetry recovers in later divisions (25, 31). This recovery could be due in part to the initiation of *cwn-1* and *cwn-2* Wnt ligand expression at this time (Fig 1D), but observations in isolated cells suggest that POP-1 asymmetry becomes cell-autonomous in later development [25]. To investigate this *in vivo*, we determined the Wnt-dependence of nuclear β-catenin levels by examining SYS-1 and WRM-1 nuclear localization in Wnt ligand-deficient mutants (Fig 4, S7 Fig). We examined embryos carrying null mutations in the Wnt ligands *cwn-1* and *cwn-2* and treated with *mom-2* RNAi to disrupt all three early embryonic Wnt ligands. We also observed similar phenotypes in embryos treated with *mom-2* RNAi derived from mothers homozygous mutant for *mig-14* (*Wntless*), which is required for Wnt ligand secretion [52,56], and after overexpression of the secreted Wnt inhibitor *sfrp-1* (S7 Fig) [44].

Consistent with previous results, loss of Wnt ligands caused significant decreases nuclear SYS-1 concentration in posterior daughters (p<10^−13^), particularly prior to the 28-cell stage. This resulted in quantitative decreases in average SYS-1 sister asymmetry prior to the 28-cell stage (p<10^−23^), although sister asymmetry still occurred in most divisions (Fig 4A, 4B and 4F). Sister asymmetry in Wnt ligand mutants is not significantly different from the wild type after the 28-cell stage, suggesting that SYS-1 sister asymmetry may become cell-autonomous in later development, although recovery of zygotic *mom-2* expression or incomplete knockdown could also play a role. The difference in SYS-1 sister asymmetry between daughters of ligand-expressing and non-expressing parents was lost in the Wnt ligand mutants, suggesting the difference is due to the expression of ligand itself and not founder cell effects (S8 Fig). We observed no significant changes in SYS-1 cousin enrichment in any Wnt ligand mutant background (Fig 4C), even in cells with low β-catenin asymmetry.

Wnt ligand mutants had pronounced defects in division orientation (Fig 4D), with over 60% of all divisions deviating significantly from the range of orientations seen in wild-type embryos (p<0.0001), consistent with the known role for Wnt ligands in spindle orientation [11,14,51]. We observed no overall correlation between abnormal division orientation and asymmetry levels or cousin enrichment (S8 Fig), but divisions that were “reversed”, with higher nuclear SYS-1 in the anterior daughter (Fig 4E), were more likely to also have defective division orientation (Fig 4F). Although *cwn-1;cwn-2* double mutants are embryonically viable [43], we observed “reversed” polarity divisions where the anterior cell had higher nuclear SYS-1 in 2 of 6 *cwn-1;cwn-2* double mutant embryos with no RNAi treatment (S8 Fig), indicating these ligands are important for normal patterning. We also observed that the *cwn-1;cwn-2* +*mom-2* RNAi mutants have a synergistic increase in defective divisions orientations and reversed divisions over *cwn-1;cwn-2* mutant embryos or *mom-2* RNAi alone (orientation: 61% vs. 13.9% and 38.2% respectively; reversed: 7.6% vs. 0.4% and 3.1%), suggesting that all three Wnt ligands play a role in robust division orientation.

The *mom-5*/Frizzled receptor is reported to play a role in SYS-1/POP-1 asymmetry throughout development [25,31], so we investigated whether it might be essential for cousin enrichment. We examined Venus::SYS-1 nuclear localization in embryos carrying a maternal-effect lethal mutation in the only embryonically-expressed Frizzled receptor, *mom-5*. We found that mutation of *mom-5* caused a nearly complete loss of nuclear SYS-1 prior to the 28-cell stage (Fig 4A and 4B) as well as a significant decrease in the asymmetry between sister cells in later divisions (Fig 4B and 4F). We observed similar defects in division orientation and reversals to those seen in the Wnt ligand mutants (Fig 4F). In contrast to the Wnt ligand mutants, we also observed a significant decrease in SYS-1 cousin enrichment in the *mom-5* mutant embryos (Fig 4C, S8 Fig). Even in the small proportion of cells (20%) for which SYS-1 asymmetry is normal in both parent and daughter cells, SYS-1 cousin enrichment is significantly reduced relative to wildtype (1.37 vs. 1.62, p = 0.003). This indicates SYS-1 cousin enrichment depends on Frizzled receptor but not Wnt ligand, suggesting it occurs through a ligand-independent process and is not an inevitable result of SYS-1 asymmetry.

### Proteasome function is required for β-catenin cousin enrichment but not TCF cousin enrichment

Recent work [57] showed that the RPT-4 proteasome subunit plays a key role in the SYS-1 nuclear asymmetry in the earliest embryonic divisions. Furthermore, the centrosomal protein RSA-2 reduces nuclear SYS-1 by promoting its degradation by the proteasome [57]. We investigated whether these proteins might affect SYS-1 cousin enrichment and asymmetry in later embryonic divisions. We found that *rpt-4* RNAi reduces SYS-1 asymmetry in divisions both before and after the 28-cell stage, and it significantly reduces SYS-1 cousin enrichment (Fig 4A, 4B and 4C and S9 Fig). Although *rsa-2* RNAi led to increased nuclear SYS-1 (Fig 4A) it did not affect SYS-1 asymmetry or cousin enrichment (Fig 4B and 4C). We examined whether proteasome function was also important for the regulation of the β-catenin WRM-1 and found that RNAi against *rpt-4* causes similar decreases in WRM-1 asymmetry and cousin enrichment (S9 Fig). In contrast, POP-1 asymmetry and cousin enrichment were not affected by *rpt-4* RNAi (S9 Fig). Together this suggests that β-catenin cousin enrichment is likely regulated at least in part through the proteasome.

### A TCF-inducible reporter is preferentially expressed in cells signaled by Wnt in consecutive cell cycles

**Fig 5:**
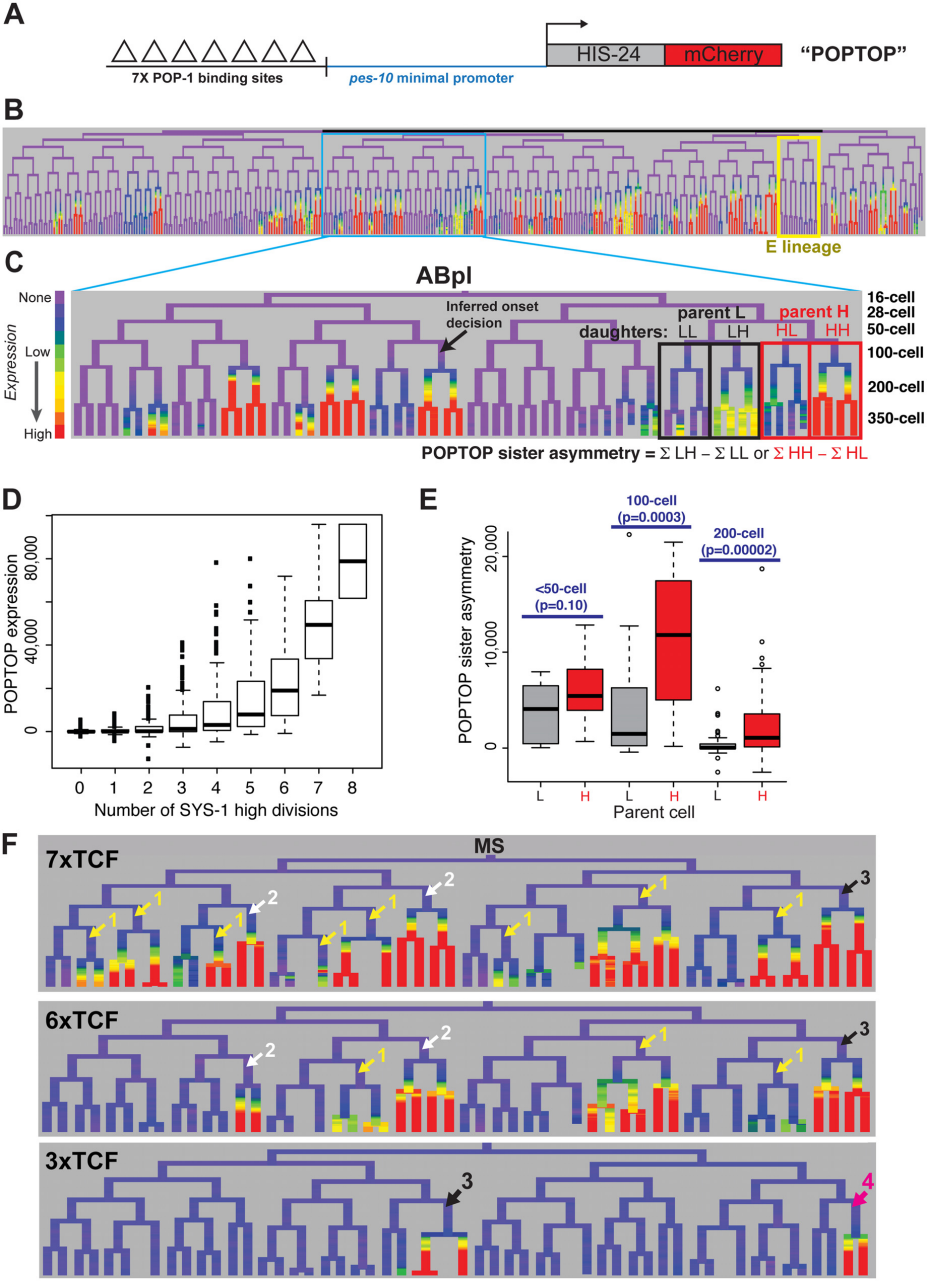
A reporter of POP-1-dependent activation shows stronger activity in double-posterior lineages. A) POPTOP construct [59], a minimal promoter driven by 7 TCF binding sites. B) Expression of POPTOP in the embryo. Expression is limited to descendants of posterior daughter cells (right branches, histone-mCherry reporter is stable even after transcription ceases). C) Detail of expression of POPTOP in the ABpl lineage. We infer that expression initiated in the common ancestor of multiple expressing cells, but visualization lags by ~30 minutes, consistent with previous estimates of the time required for transcription, translation and mCherry maturation [60,83]. POPTOP activity for each cell is the average expression of that cell and its descendants (boxes). We then calculate differential activity between sister sublineages (e.g. ABplpppp HH sublineage minus ABplpppa “HL” sublineage). In the case marked, the SYS-1 High-High cell (HH) expresses POPTOP more strongly than its sister lineage (HL). D) POPTOP levels increase with number of previous divisions with high levels of nuclear SYS-1, as expected if it is specifically transcribed in posterior daughters. E) POPTOP sister asymmetry as outlined in panel C (SYS-1 high cells: red, SYS-1 low cells: grey) clustered by the time at which the division occurred. p-values test for significant differences between Xpp and Xap cells (Wilcoxon rank-sum test). POPTOP expression is more asymmetric in lineages of parent cells with high levels of nuclear SYS-1. Asymmetry is highest at the 100-cell stage, when most lineages initiate POPTOP expression. F) Enhancers with seven (*POPTOP*), six, and three TCF sites; cells with expressing daughters are marked by the number of consecutive high nuclear SYS-1 parents. A synthetic enhancer with a single TCF site showed no detectable embryonic expression (see S10 Fig). Fewer TCF sites leads to more restricted expression in multiply posterior lineages.

We next sought to determine whether the cousin enrichment of nuclear β-catenin and TCF affects target expression. We measured the ability of POP-1 to activate transcription by lineage analysis of a synthetic Wnt-activated reporter construct. This “POPTOP” (POP-1 TOPFLASH) reporter contains seven consensus TCF binding sites capable of binding POP-1 [58] and a minimal (*pes-10*) promoter driving histone-mCherry expression (Fig 5A), and is regulated by the Wnt pathway in L3 larvae [59]. We quantified POPTOP expression in all embryonic cells through the 350-cell stage by automated lineage tracing. Due to the delay between transcription initiation and detection of stable histone-mCherry in the rapidly dividing cells of the *C*. *elegans* embryo, reporter expression is not observed in the activating cell itself, but in its daughter cells [59–61]. Therefore, we developed a “POPTOP sister asymmetry” metric to measure the impact of an individual division on POPTOP expression. “POPTOP activity” in each cell is the average fluorescence in all of that cell’s descendants (Fig 5B and 5C); this activity metric integrates expression across that cell’s ancestors and descendants. We then defined “POPTOP sister asymmetry” as the difference in this “POPTOP activity” between sister lineages (Fig 5C); this corrects for any expression that occurred prior to the division. Embryonic POPTOP activity is exclusive to posterior daughter lineages (Fig 5B–5D), and POPTOP expression requires *pop-1* (S10 Fig).

Like synthetic TCF site reporters in other systems [62], the POPTOP reporter is not activated in all locations where Wnt signaling occurs. For example, we detected no consistent POPTOP sister asymmetry in the division of the EMS cell (Fig 5B), where target expression and cell fate is well-established as POP-1 and Wnt-dependent [14,28,31,63,64], suggesting that binding sites for additional factors are required for Wnt target expression in this division. Similarly, we observed POPTOP activity in only a subset of later lineages even though many additional cells with high nuclear β-catenin are born early enough to allow for mCherry transcription, translation and maturation. These patterns were consistent across independent strains made with the same construct so are unlikely to result primarily from transgene integration site effects. The quantitative variation in POPTOP expression suggests that the ability of POP-1/SYS-1 to activate transcription in response to Wnt signaling is not uniform throughout the embryo by lineage or across developmental time.

We observed a striking trend towards higher POPTOP activity and sister asymmetry in SYS-1 High-High lineages (Fig 5C and 5E). Activity increases significantly with number of SYS-1 High-High ancestors, even after accounting for the total number of SYS-1 High ancestors (p<10^−15^). This suggests that the higher levels of β-catenin and lower levels of POP-1 observed in cells that received Wnt signals in multiple consecutive divisions impact POP-1’s ability to activate transcription. POPTOP asymmetry also increased with birth time through the 100-cell stage (Fig 5E), suggesting that POP-1 is a less-potent activator in early divisions, consistent with the lower β-catenin localization we see at those times. While we saw decreased asymmetry in 200-cell stage divisions, this likely reflects the lack of time for mCherry production and maturation between these divisions and the end of our movies. Cells at posterior locations within the embryo have slightly higher POPTOP activity, but many POPTOP-expressing cells are present in anterior locations (S10 Fig).

If the cousin enrichment of POP-1 and SYS-1 determines which cells express POPTOP, reducing the binding of POP-1 should further bias expression to the double-posterior cells where activation potential is highest (i.e. “SYS-1 High-High” cells with the highest ratio of β-catenin to POP-1). To test this, we generated POPTOP variants with six, three or one TCF binding sites and compared their expression to the seven-site POPTOP pattern by lineage analysis. The comparison of these synthetic enhancers with different affinities for TCF (via the different numbers of binding sites) serve as a read-out for the amount of the POP-1/SYS-1 activating complex in each cell throughout development. A single TCF site was not sufficient to induce expression in embryos (S10 Fig). An enhancer with six sites drove slightly more restricted expression than that with seven sites and had a stronger bias towards SYS-1 High-High lineages (Fig 5F). With three sites, activity was not observed until the 200-cell stage and expression was later and weaker and even more restricted to a handful of triple-posterior lineages (SYS-1 High-High-High; Fig 5F, S10 Fig). We also examined an enhancer containing six copies of an optimal TCF site and a “Helper” site that binds the C-clamp and is reported to stabilize the interaction between DNA and the TCF/β-catenin complex but not repressive TCF complexes [65]. Expression of this reporter was also restricted to posterior branches and was expressed in a similar number of lineages as the six TCF site enhancer (S10 Fig). Across all embryos examined, the percentage of initiating cells that were SYS-1 High-High were 70% in 7xTCF, 80% in 6xTCF, 93% in 6x(TCF+Helper) and 89% in 3xTCF. The cells expressing the three-site reporter also have quantitatively higher expression of the seven-site POPTOP reporter: six of the nine cells with the highest level of POPTOP activity express the 3xTCF reporter. This indicates that these cells have the highest levels of POP-1/SYS-1 activated transcription and demonstrates that this activity is highest in SYS-1 High-High cells.

### Posterior lineage-specific transcription factors represent potential targets of *pop-1*

One drawback of synthetic enhancer approach is that repression cannot be assayed, and repression of targets by POP-1 is one of its critical functions [40,66]. All but one of the direct Wnt target genes known in the *C*. *elegans* embryo are expressed in either the daughters of the first Wnt signaled division in the embryo (EMS) and therefore unsuitable for evaluating the effect of consecutive Wnt signals, or in late stages which cannot be easily analyzed by our approach due to the movement of the embryo [13,40,64,66,67]. Therefore, we sought to identify additional endogenous embryonic target genes that might require POP-1 for repression of expression.

**Fig 6:**
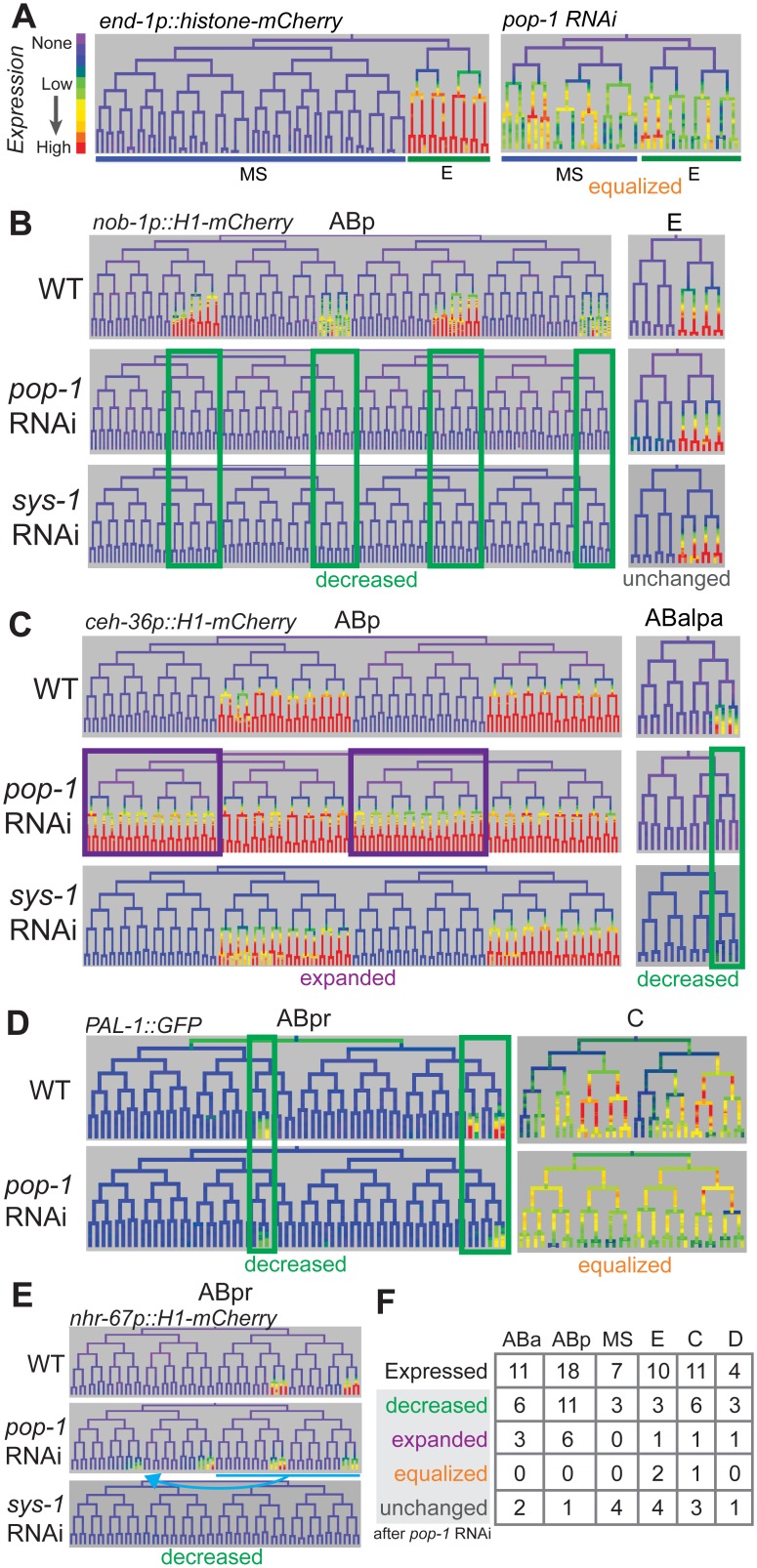
Most genes expressed in multiple posterior lineages are either activated or repressed by *pop-1*. A) Expression driven by the *end-1* promoter in the EMS lineage is high in the posterior E descendants and absent from the anterior MS descendants. Depleting *pop-1* by RNAi increases *end-1* promoter activity in the MS lineage and decreases it in the E lineage, equalizing expression as previously demonstrated. B) *nob-1* expression in posterior daughter-derived lineages is lost after both *pop-1/TCF* and *sys-1/β-catenin* RNAi (green boxes) in the ABp lineage, but expression is unchanged in the E lineage. C) Expression of *ceh-36* in ABp is expanded into previously unexpressing cells with *pop-1* RNAi (purple boxes) but unchanged in response to *sys-1* RNAi. Expression of *ceh-36* in ABalpa is decreased in response to *pop-1* and *sys-1* by RNAi (green boxes). D) In response to *pop-1* RNAi, expression of *pal-1* in AB lineage (ABpr shown) is greatly decreased, but expression in the C lineage becomes equalized. E) A known anterior to posterior fate transformation (blue underline, arrow) caused by *pop-1* RNAi causes ectopic expression of *nhr-67*, while normal expression is decreased. The loss of expression from the *nhr-67* promoter in response to *sys-1* RNAi suggests that *nhr-67* depends on *pop-1/sys-1* for activation. F) Table showing the number of genes expressed in each sub-lineage and the response to pop-1 RNAi in each. See full lineage diagram in Fig 1E.

We searched a database of cellular resolution expression patterns [54] and our additional unpublished patterns for genes expressed in lineages derived from posterior daughter cells (referred to as “posterior lineages”) since this is where TCF targets should be expressed [25]. This identified 22 genes (Table 1), including one known Wnt target gene, *ceh-13*, and the Wnt ligands *mom-2*, *cwn-1*, and *cwn-2* [67]. Many of these candidate targets are expressed in repetitive patterns, with multiple posterior daughters at a given time initiating expression simultaneously. The expressing lineages generally produce multiple cell types. For example, the *nhr-67/*Tlx promoter drives expression in the “SYS-1 High-High” great-granddaughters of the ABplp and ABprp cells (Fig 6E). All but one of the predicted genes’ predicted upstream regulatory sequences contain consensus TCF binding sites, many with TCF “Helper” sites [65] and the TCF motif is significantly enriched (2.2-fold, p = 0.0004) in these sequences relative to random upstream regions (Table 1, S4 Table). These genes thus represent potential direct transcriptional targets of Wnt signaling, although we did not rule out indirect regulation. Most of these genes encode conserved transcription factors and are essential for viability [68] (Table 1).

**Table 1:** Posterior lineage-dependent genes are targets of TCF.

Gene	Homologs	TCF regulation class	TCF sites	TCF w/Helper sites	Regulatory Region (Kb)
*ceh-13* * †	*HOX1/labial*	Moderately Activated in ABp	10	0	8.2^
*ceh-27* *	*NKX/scarecrow*	Moderately Repressed in ABp	1	1	3.4
*ceh-36* *	*OTX/orthodenticle*	Strongly Activated in Aba; Strongly Repressed in ABp	6	0	5.1
*ceh-43* *	*DLX/distalless*	Indirect	0	0	2.4
*ceh-6* *	*POU3F4*	Strongly Activated in Aba; Weakly Activated in ABp	6	2	6.8^
*cwn-1*	*WNT*	Moderately Activated	2	0	1.9
*cwn-2*	*WNT*	Weakly Activated in ABa and E; Weakly Repressed in ABp	7	1	6.1
*elt-6* #	*GATA4/5/6*	Moderately Activated	2	0	2.4
*end-1* †	*GATA1*	Activated + Repressed	2	2	2.5
*end-3* †	*GATA1*	Activated + Repressed	3	1	2.2
*ets-7*	*ELK*	Moderately Activated	2	0	1.5^
*mom-2*	*WNT*	Unregulated	1	0	3.1
*mir-57*	microRNA	Weakly Activated	1	1	2.3
*nhr-25* *	*NR5A*	Weakly Activated in Aba; Moderately Activated in ABp	6	2	8.9^
*nhr-67* *	*NR2E/tailless*	Weakly Activated in ABa; Moderately Activated in MS	7	1	5.1
*nob-1* *	*HOX9-13/Abd-B*	Strongly Activated in ABp and C	5	3	5.4
*pal-1* *	*CDX/caudal*	Moderately Activated in AB Weakly Activated in D; Activated + Repressed in C	6	1	4.6^
*pax-3* *	*PAX3/paired*	Moderately Activated	2	1	2.6
*sem-2* *	*SOX11*	Strongly Repressed in AB	6	0	3.9
*tbx-11*	*TBX2* subfamily	Moderately Repressed except in ABp	3	0	2.1
*tlp-1* #	*ZNF503/703*	Strongly Repressed in AB	4	2	5.1
*unc-130* *	*FOXD*	Weakly Activated	11	2	10.5^
*unc-30*	*PITX*	Strongly Repressed	6	3	6.2^
*vab-7* *	*EVX/even-skipped*	Moderately Activated	1	1	3.6

* deletion lethal,

^†^ known embryonic target [40,64,67],

^#^ known post-embryonic target [85,86],

^^^ fosmid protein fusion reporter, so additional elements may lie outside the 5’ regulatory region.

### Most posterior lineage-specific transcription factors require *pop-1* for either repression or activation

We tested whether POP-1 regulates lineally posterior expression patterns by examining their expression after using RNAi to deplete Wnt pathway components. We used lineage analysis to examine the effect of depleting *pop-1* by RNAi. Previous work showed that *pop-1* is required in the division of the EMS cell both to activate expression (with SYS-1) in the posterior daughter E, and to repress expression in the anterior daughter MS, which we refer to here as “dual” regulation [66]. Our quantitative microscopy approach similarly shows equalized expression of the Wnt target promoters of *end-1* and *end-3* in MS and E descendants after *pop-1* RNAi (Fig 6A, S11 Fig). We also found that a reporter for the known Wnt target *ceh-13*, loses expression in many lineages when POP-1 levels are reduced, similar to previous lower resolution reports [67], suggesting POP-1 is required for its activation but not repression, as is true for several postembryonic targets (e.g. *psa-3*, *ceh-22*, *mab-5*) [58,69,70].

We tested the role of *pop-1* in activating and repressing the 21 new posterior-lineage genes by quantifying their expression after *pop-1* RNAi. Expression of each gene except *mom-2* was altered after *pop-1* RNAi, indicating that they are directly or indirectly regulated by *pop*-1. We found that expression could change independently in different sublineages. For example, expression of *nob-1* is completely lost in the ABp lineage after *pop-1* RNAi, but unchanged in the E lineage (Fig 6B). We observed lost or reduced expression in some sublineage of 15 genes indicating a role for *pop-1* in activation (Table 1, S11 Fig). We also observed that *pop-1* RNAi caused increased or expanded expression for seven genes; for example *pop-1* RNAi causes expression of *ceh-36* in the anterior branches of ABp where it was previously not expressed (Fig 6C). For one gene, *pal-1*, expression was equalized between expressing and non-expressing sublineages of the C lineage, while it was reduced in the ABa and ABp lineages (Fig 6D). Many genes show the same response to POP-1 knockdown in all lineages where they are expressed, others show changes in some lineages but no change in others; three genes showed regulation by activation and repression in different lineages, *ceh-36*, *cwn-2*, and *pal-1* (Fig 6C and 6D and S11 Fig). These differences are intrinsic to each target and not due to variable knockdown of *pop-1*, as changes were reproducible across replicates for each gene (n = 4–5 per target; S11 Fig). It should be noted that loss of *pop-1* is known to cause anterior to posterior fate transformations in the early AB founder cells (e.g. ABpla→ABplp) [14], but these changes cannot explain the expression loss in posterior lineages that we observe for many genes. Furthermore, if the gains in expression we observe for some genes were an indirect cause of these fate transformations, we would expect the expression in anterior founders to match the expression in posterior founders. This was only observed in the case of *nhr-67* (see below), *unc-30*, *sem-2*, and *ceh-36* expansion in ABp, although we cannot rule out additional later anterior to posterior fate transformations for the other genes with expanded expression. In any case, these genes are regulated by *pop-1*, directly or indirectly via other posterior determinants that depend on POP-1. For example, *ceh-36* expression is either directly repressed by POP-1 in ABpla and ABpra or it is activated by an early lineage fate regulator that is directly repressed by POP-1 in ABpla and ABpra; in either case a critical factor is repressed by POP-1.

To verify that these targets are indeed regulated by the Wnt pathway, we examined the expression when other Wnt pathway effectors were knocked down. In embryos, POP-1-mediated transcriptional activation, but not repression, requires the β-catenin homolog SYS-1 [28,31]. If only a subset of targets require *pop-1* for activation, expression of only these targets should depend on *sys-1*. Consistent with this, we found that none of the expression domains that expanded after *pop-1* RNAi require *sys-1* for expression (one exception, see below), while all expression domains that were reduced after *pop-1* RNAi also showed reduced expression with *sys-1* knockdown (Fig 6B and 6C and S11 Fig). We therefore conclude that the genes which show reduced expression with *pop-1* and *sys-1* RNAi require *pop-1* for activation, while the genes that show expanded expression with *pop-1* RNAi and no dependence on *sys-1* require *pop-1* for repression. One unique case was *nhr-67*, which absolutely requires *sys-1* for expression, but shows mixed results with *pop-1* RNAi: up to 68% reduction in the sublineages in which it is normally expressed but ectopic expression in additional posterior lineages. Since the gained expression is consistent with anterior→posterior fate changes in the AB founder cells known to occur in *pop-1* mutants [14], we therefore classified *nhr-67* as likely requiring *pop-1* and *sys-1* for activation (Fig 6F).

As a further check that each target is regulated by the Wnt/β-catenin asymmetry pathway, we depleted *lit-1*/NLK by RNAi (S11 Fig). Loss of *lit-1* leads to high levels of nuclear POP-1 in all cells, preventing Wnt target expression [30], regardless of whether *pop-1* normally is required for activation or repression. We found that *lit-1* RNAi caused widespread reduction in expression for most tested targets, but *ceh-43* and *ceh-27* in the ABa sublineage instead showed a widespread expansion of expression (S11 Fig) inconsistent with simple regulation by *pop-1*. Overall, we conclude that *pop-1* can regulate expression independently in different lineages, and in most cases regulates either anterior repression or by posterior activation but not both.

### Genes activated by *pop-1* are biased towards double-posterior lineages

**Fig 7:**
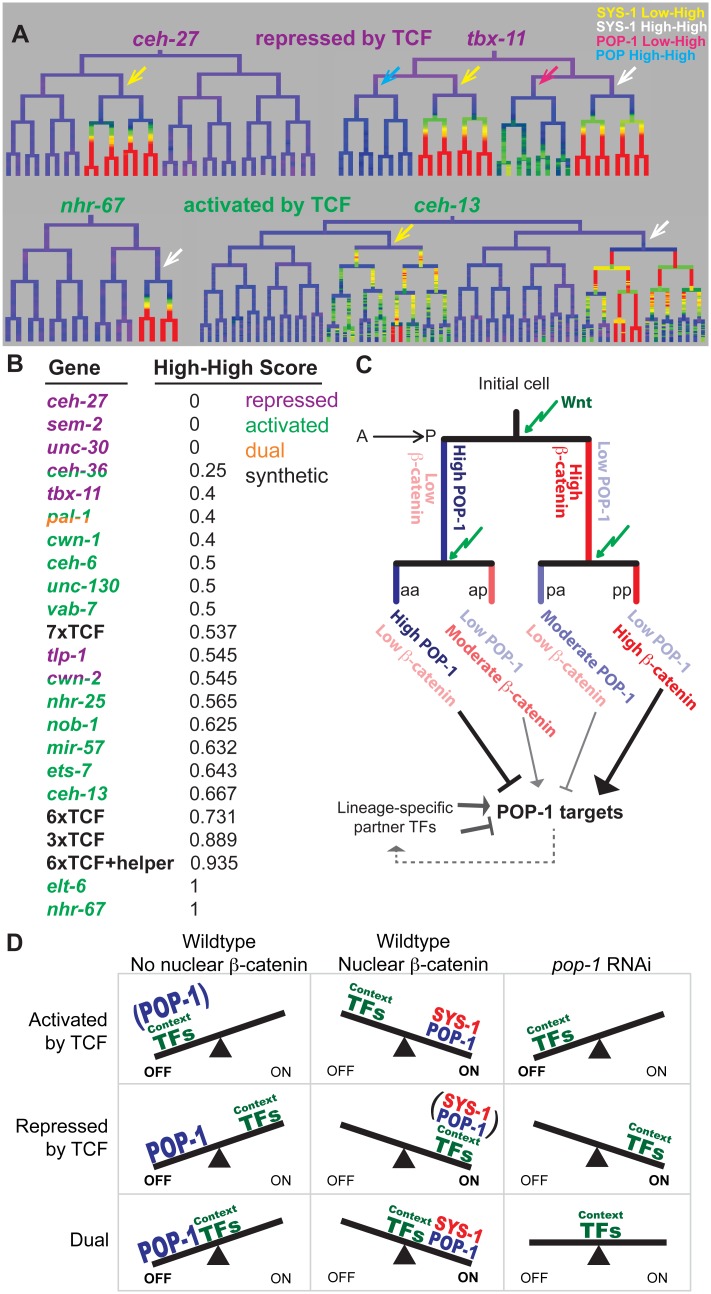
Updated model for TCF-mediated lineage diversification. A) Examples of wild-type expression patterns for genes that require TCF primarily for repression or activation. Repressed genes have exclusive (*ceh-27*) or equal (*tbx-11*) expression in SYS-1 Low-High lineages (yellow arrows), while activated genes have exclusive (*nhr-67*) or stronger (*ceh-13*) expression in SYS-1 High-High lineages (white arrows). B) Expression in SYS-1 High-High lineages for all genes and synthetic enhancers assayed. SYS-1 High-High score is the fraction of expressing lineages that are SYS-1 High-High. Expression is significantly more limited to SYS-1 High-High lineages for genes that require POP-1 for activation (green) than repression (purple) (p = 0.004, Wilcoxon rank sum test), as predicted by our model. C) A proposed model of TCF-mediated lineage patterning in *C*. *elegans* embryos. POP-1 and β-catenin concentrations vary depending on previous signaling history. Cells that are consecutive posterior daughters (pp) have the highest β-catenin levels and are predicted to strongly activate POP-1 targets. Consecutive anterior daughters (aa) have the highest POP-1 levels and are predicted to most strongly repress POP-1 targets. Single-anterior and single-posterior daughters (pa, ap) are predicted to have intermediate levels of repression and activation, respectively. D) Proposed model for the potential role of context transcription factors in determining the type of TCF regulation. When POP-1 is present, all targets are repressed by high levels of nuclear POP-1 and activated by SYS-1 and moderate levels of POP-1. We hypothesize that POP-1 RNAi reveals the activity of the context factors co-regulating a gene. We predict genes requiring TCF for activation are co-regulated by weak context factors that cannot activate transcription in the absence of POP-1, while genes requiring TCF for repression are co-regulated by context factors that can activate robust expression independently. Genes that are both activated and repressed (dual) could be co-regulated by context factors that produce moderate expression in the absence of POP-1.

We predicted that if quantitative differences in nuclear POP-1 and SYS-1 are important for endogenous expression patterns, then genes expressed in SYS-1 High-High lineages would be more likely to depend on *pop-1* for activation. We quantified the extent to which each target’s expression is enriched in SYS-1 High-High lineages relative to SYS-1 Low-High lineages (all targets were selected based on expression in either Low-High or High-High cells). The *pop-1*-activated targets were significantly more likely to be expressed in SYS-1 High-High lineages as compared to the *pop-1-*repressed targets (p = 0.004; Wilcoxon rank sum test; Fig 7A and 7B). We conclude that genes expressed primarily in double-posterior SYS-1 High-High lineages are more likely to depend on *pop-1* for transcriptional activation.

## Discussion

### A transmitotic enrichment of Wnt effectors

Previous studies in *C*. *elegans* embryonic development identified asymmetric localization of POP-1/TCF and SYS-1/β-catenin in almost all dividing cells, and showed this process depends on the frizzled receptor but not Wnt ligand [14,25]. By quantifying this phenomenon, we identified variation in the nuclear localization of these proteins depending on cells’ lineage history, and found that this cousin enrichment depends on the Frizzled receptor but not Wnt ligand. We demonstrated that the cousin enrichment of POP-1/TCF and SYS-1/β-catenin impacts synthetic target gene transcription and find evidence that genes requiring TCF for activation are preferentially expressed in cells with high cousin enrichment, suggesting that this phenomenon may influence endogenous target expression. Thus quantitative differences in the nuclear localization of POP-1/SYS-1 in response to consecutive signals represent an important new mechanism that contributes to context-specific gene regulation.

The transmitotic enrichment of POP-1/TCF and SYS-1/β-catenin produces a greater diversity of transcriptional states than previously recognized (Fig 7C). POP-1 is thought to function as a repressor in the absence of SYS-1, while addition of SYS-1 can convert it to an activator. Our observations suggest an extended model for POP-1-mediated regulation (Fig 7C): In consecutively signaled cells, nuclear POP-1 levels are low and SYS-1 levels are highest, implying the most potent activation due to the highest dose of the POP-1/SYS-1 activation complex. In contrast, posterior daughters of cells whose parents had low Wnt activity have moderate levels of nuclear SYS-1 and low nuclear POP-1, which should result in weaker activation due to a lower dose of the activation complex. We demonstrated that this is the case for our synthetic POPTOP variants: targets requiring higher levels of the POP-1/SYS-1 activation complex (e.g. because of a reduced number of TCF binding sites) are more likely to be activated in consecutively signaled cells than in cells with unsignaled parents. Similarly, a higher dose of POP-1 alone may strongly repress expression in consecutively unsignaled nuclei, with moderate POP-1 levels leading to weaker repressive dose in unsignaled nuclei. Indeed, *tbx-11*, which requires TCF for repression, shows no expression in POP-1 High-High sublineages and weak expression in POP-1 Low-High sub-lineages (Fig 7A). Our model is consistent with the tendency for genes expressed in double-posterior lineages to depend on *pop-1* for activation, although additional experiments will be required to determine the mechanisms linking these quantitative differences.

### Possible mechanisms to explain cousin enrichment

We found that protein turnover contributes to both sister asymmetry (as shown previously [57]) and cousin enrichment of the β-catenins, SYS-1 and WRM-1. β-catenin may be protected from degradation by nuclear localization or other mechanisms in Wnt signaled cells, subsequently allowing more β-catenin to be inherited by the daughters of these cells. The frizzled receptor gene *mom-5* promotes the cousin enrichment of SYS-1, possibly by protecting SYS-1 from proteasomal degradation or other mechanisms. Other mechanism must regulate the anterior cousin enrichment of POP-1, since it is not affected by proteasome RNAi.

Multiple Wnt pathway components, including β-catenin and frizzled receptors, are preferentially localized to the posterior during mitosis [10,28,34,35,71]. This localization asymmetry could lead to biased inheritance of Wnt pathway proteins and thus differential concentrations of POP-1 or other upstream regulators. Biased inheritance of post-translational modification states, such as phosphorylation, of these proteins could also influence pathway activity, such as by controlling the nuclear localization of POP-1 [30,36,37]. Positive transcriptional feedback seems unlikely to be essential, since SYS-1/β-catenin has been reported to have uniform transcription and translation [28], which we confirmed by lineage analysis, and GFP::POP-1 was similarly expressed from a uniform promoter in our experiments. It also remains to be determined if this enrichment exists in the other species and cell types and if it occurs in canonical Wnt signaling or is limited to the Wnt/β-catenin asymmetry pathway. Wnt signaling from the niche patterns asymmetric stem cell divisions, with higher nuclear β-catenin in the stem cell daughter, so enrichment could explain how asymmetric fate decisions are reinforced [8].

### Context-specific regulation of POP-1 targets

POP-1-dependent transcriptional activity as measured by the POPTOP reporter was biased against very early divisions. The presence of seven consensus binding sites in POPTOP should lead to much higher affinity for POP-1 than occurs at endogenous enhancers, and thus predicts broad expression. The lack of early expression suggests that temporal mechanisms modulate POP-1-dependent activation. Temporal regulation by other transcription factors uses a concentration-based mechanism; for example, PHA-4/FoxA concentration increases over time during pharyngeal development, allowing regulation of early targets through high-affinity PHA-4 binding sites and late targets through lower-affinity sites [72]. The increasing nuclear concentration of β-catenin over time suggests a similar mechanism could regulate temporal patterning of TCF targets. We hypothesize that the activation of the TCF reporters depends on a threshold with a minimum SYS-1 and maximum POP-1 concentration, with the threshold varying with the number of TCF sites. However, we also observed that some cells that do not activate the POPTOP reporter have similar nuclear concentrations of POP-1 and SYS-1 as those that activate POPTOP consistently. These cells are found disproportionately in the ABal and E lineages, suggesting that the threshold for activation may vary by lineage.

The lack of POPTOP expression in early cells where POP-1 is known to regulate expression, such as E, suggests that additional transcription factors are required in those cells for expression. Consistent with this, transcription factors that cooperate with POP-1 to regulate expression have been identified for many targets, such as *med-1* and *med-2* which act together with POP-1 to activate *end-3* in the E lineage [13,42,63,64,73,74]. Thus, the role of POP-1 in activation and repression likely depends not only on its intrinsic activity, but also of the identity and activity of other context-specific transcription factors, which can help overcome the low early TCF/β-catenin concentrations. We expect that all of the targets we identified have additional co-regulators, since none of their expression patterns are similar to the TCF reporters we examined. Our proposed model (Fig 7D) is that a gene’s basal expression pattern in the absence of POP-1 is determined by the activity of additional transcription factors. If these factors are sufficient to initiate transcription, POP-1 will be required to repress expression in the anterior daughter, while if they are not sufficient to induce transcription, POP-1 will instead be necessary for activation in the posterior daughter. Only if the additional factors bring a gene’s activation to a sub-saturation state will POP-1 be required both for full posterior activation and for anterior repression, as observed in the E and C lineages. Along the sliding scale from activation to repression, we find that many targets utilize POP-1 for activation, raising the possibility that some context factors may provide specificity but are not sufficient for activation alone. In addition to context-specific transcription factors, others have shown that regulation of chromatin state also provides specificity to TCF/β-catenin target activation [75]. Our results indicate that transmitotic enrichment of TCF/β-catenin could provide additional context to activate particular target genes in “high-high” lineages, perhaps even without combinatorial regulatory partners.

In summary, we used a quantitative approach to identify the nuclear enrichment of TCF and β-catenin in response to consecutive Wnt signals during development, and showed this further patterns developing cells by impacting the activation and repression of TCF/β-catenin targets. Given the conservation of the Wnt pathway across metazoans it will be important to determine whether similar patterning occurs in other species.

## Materials and Methods

### *C*. *elegans* strains and transgenes and growth

Strains and alleles are listed in S1 Table. All *C*. *elegans* strains were grown on NGM/OP50 plates using standard methods [76]. The ubiquitous histone-mCherry lineaging strain JIM113(*ujIs113* [*pie-1* promoter::mCherry::H2B; *nhr-2* promoter::mCherry::HIS-24-let-858UTR; *unc-119(+)*] II) was generated by co-bombardment of pAA64::mCherry-H2B [77] and pJIM20_*nhr-2* [54] and outcrossed three times. The *ujIs113* lineaging reporter was crossed into transcriptional reporters for the Wnt ligands *cwn-2* (*deEx103*), *egl-20* (*deEx102*), *lin-44*(*deEx100*), and *mom-2* (*deEx104*) each driving NLS-tagged GFP [43]. We crossed strains expressing [wrm-1p::WRM-1::GFP + pRF4] (*neIs2* [35]), [sys-1p::Venus::SYS-1 + pttx-3::dsRed] (*qIs95* [28]), [sys-1p::Venus::SYS-1(stops)] (*qIs130* [28]), [sys-1p::GFP::POP-1 unc-119(+)] (*qIs74*, [55]), [med-1p::GFP::POP-1; pRF4] (*wIs117*, [40]), and *POPHHOP* ([6xHMG-helper::NLS-GFP dpy-20(+)], [65]) into a strain carrying *ujIs113*. The GFP::POP-1 transgene we examined, qIs95 (sys-1p::GFP::POP-1), can rescue the *pop-1(q624)* mutation, which disrupts the DNA binding domain of POP-1 [55]. Expression from the *sys-1* promoter as assessed by the Venus::SYS-1(stops) reporter is largely ubiquitous and equivalent between SYS-1 “high” and “low” cells (S2 Fig) [28]. *neIs2*(GFP::WRM-1) was analyzed in a *wrm-1(tm514)* null background to allow normal localization [35]. *qIs95* was further crossed into *mom-5(or57)*, *mig-14(or78)* [52] and *cwn-1(ok546);cwn-2(ok895)* [43] mutant backgrounds. POPTOP (*syIs187* [59]), and [Psys-1::mCherry::SYS-1] (*uiwIs4*, [57]) were crossed into the ubiquitous histone::GFP-expressing strain RW10029 for lineage analysis. POPTOP variants with 6, 3 or 1 TCF sites were synthesized (Blue Heron Biotech, Bothell, WA), cloned into the original POPTOP plasmid (Addgene #34848)[59], and introduced to *C*. *elegans* by microparticle bombardment [78]. Maternal RNAi targeting *lit-1*, *mom-2*, *pop-1*, *sys-1*, *rpt-4*, and *rsa-2* was performed by feeding as previously described [57,79]. Embryos were examined for control phenotypes to ensure RNAi was effective (i.e. MS->E fate conversion for *pop-1*, E->MS fate conversion for *lit-1* and *mom-2*, cell cycle delays for *rpt-4*, and embryonic arrest for *rsa-2* and *sys-1*) and embryos without these phenotypes were excluded from analysis.

### Lineage analysis

We collected 4D image sequences with a Leica SP5 TCS Resonance-scanning confocal microscope as previously described [50,51] and traced lineages using StarryNite [47,80] and Acetree [48]. Embryos were mounted for microscopy by using a bead-slurry method [81]. Embryonic temperature was maintained at 22.5°C with a custom stage insert (Brook Industries, Lake Villa, IL) to enable all wildtype embryos to hatch. All embryos are listed in S2 Table. For reporters on extrachromosomal arrays (Wnt ligands), embryos were only analyzed if they showed expression of the array marker (*ajm-1*::*GFP*) in late embryogenesis. Reporter expression was quantified by calculating the mean fluorescence intensity in each nucleus (across all planes where that nucleus is present as identified by StarryNite), providing an estimate of the concentration of fluorescent molecules. We subtracted local non-nuclear background and averaged over the life of the cell to get a single mean concentration for each cell [60]. Statistical comparisons of β-catenin and POP-1 localization were performed in R (http://www.R-project.org/) [82].

### Antibody staining

Gravid JIM107 (UNC-130::GFP, wgIs76) adults were bleached and washed with M9 buffer to isolate embryos which were plated onto slides coated with poly-L-lysine and flattened with a coverslip. Slides were frozen at -80°C overnight and fixed in methanol for 15 minutes at -20°C after freeze-crack. Slides were then washed with PBS plus 0.1% Tween-20 and incubated with a blocking buffer containing 1% BSA and 0.1% sodium azide for 30 minutes at 4°C, then incubated with primary antibodies against GFP (ab290, Abcam, 1:2000) and POP-1 (mAbRL2, kind gift of Rueyling Lin, 1:25) diluted in blocking buffer overnight at 4°C in a humidified chamber. Slides were then washed, incubated with fluorescently labeled secondary antibodies (A-21124 and A-11070, Invitrogen, 1:1000) for two hours at room temperature, washed and mounted with ProLong Diamond Antifade with DAPI, and imaged using a Leica SP8 with HyD detectors. Quartets of UNC-130::GFP positive cousin nuclei in 350 cell stage embryos were identified based on embryo stage and relative position using our wildtype model [51]. Average anti-POP-1 intensity was measured using ImageJ.

### TCF site identification

For promoter fusions, the region examined for TCF sites was that found in the promoter construct, while for protein fusions, which contain the larger genomic context as part of a fosmid, the region between a gene’s TSS and nearest 5’ neighbor gene was examined. TCF sites were identified by two approaches: first, the IGV genome browser was used to identify sites using the primary position weight matrices for mammalian TCF1, TCF3 and TCF7. The second approach was to use text-find to search for the *C*. *elegans*-specific HMG motif [65]. A number of sites were found by both methods and are reported only once. For each site identified, the 20 bp upstream and downstream were scanned for GC-rich motifs matching the *C*. *elegans*-specific Helper motif [65].

## Supporting Information

S1 Table List of strains used. (XLS)

S2 Table List of embryos analyzed. (XLS)

S3 Table β-catenin levels in all embryonic cells. (XLSX)

S4 Table List of predicted TCF sites. (XLSX)

S1 Data Raw data files for embryos analyzed. For each cell analyzed, birth time is given in column 3 and the average nuclear reporter intensity over the lifetime of the cell (corrected for background) is given in column 7 (“blot”). For information on each embryo’s strain information and experimental conditions, refer to S2 Table.
(ZIP)

S1 Fig Full localization of Wnt ligand expression. **A**) We observed dynamic expression of the *mom-2* promoter, although maternal expression was undetectable. Early in development, weak expression was observed in ABpra; moderate expression in ABalp, ABarap; strong expression in ABplp, ABprp, MS, and E. Expression is constant or decays in daughter cells after roughly the 50 cell stage, suggesting the promoter is no longer active. Expression is re-activated in ABplpppapaa, (PHshL lumbar ganglion), ABplpppapap (hyp 8/9), ABplpppppaa (intestinal muscle L), ABplppppppa (death), ABplppppppp (hyp 10), ABplpppppaa (body muscle), and ABplpppppap (sphincter muscle). **B**) We observed variable expression of *cwn-2*, likely due to the nature of the extrachromasomal array, which can be silenced or lost during mitosis. Two separate lineages (and part of a third marked by an asterisk) are shown to display the variability. We observed early expression in ABarpp, ABplap, ABplpp, ABprpp, and E. Late activation of expression is also observed in the derivatives of MSaapp and MSpapp, ABaraapapaa (NSML pharynx, death), ABaraapapap (m5L pharynx), ABaraapppaa (NSML pharynx, death), ABaraapppap (m5L pharynx), ABplappaaa (P3/4 blast), and ABplappaap (P5/6 blast). **C**) We observed expression of *cwn-1* in all cells of the C lineage and variable expression in the D lineage, with stronger expression in derivatives of Caap, Cap, and Cpp. **D**) We observed expression driven by the *egl-20* promoter in the sister cells ABplppppaa and ABplppppap at the 350 cell stage and strong expression in their daughter cells: ABplppppaaa, the PVPL interneuron; ABplppppaap, the VL cell of the rectal gland; ABplppppapa, the U rectal epithelial cell; and ABplppppapp, the K rectal epithelial cell. We did not observe embryonic expression through the 1.5 fold stage in the p9/10 or p11/12 seam cells (ABplapapap, ABplapappa), the B rectal epithelial cell (ABprppppapa), or the anal depressor muscle (ABplpppppap), in which expression was reported at the L1 larval stage [41]. Expression in these cells may not begin until later in development or may be driven by elements not found in the promoter, which includes 2 kb of upstream sequence between the *egl-20* start site and the next most 5’ gene. Expression in PVPL and VL were not previously reported at L1 stage, possibly due to detection issues or because expression in these cells is later down-regulated. **E**) We observed expression driven by the *lin-44* promoter in 8 cells from L-R symmetric lineages at comma stage: the PHshL and PHshR phasmid sheath cells (ABplpppapaa, ABprpppapaa), hypodermal cells 8 & 9 (ABplpppapap, ABprpppapap), the two nuclei of hypodermal cell 10 (ABplppppppp, ABprppppppp), and two cells fated for cell death (ABplppppppa, ABprppppppa). We did not observe expression in the hypodermal cell 11 nucleus (Cpappv) at comma stage as was previously reported in the embryo [39,42] using a similar transcriptional reporter approach and *in situ* hybridization. Because similar constructs were analyzed, the major difference between these experiments is our automated lineage analysis approach to determine the identity of cell nuclei compared to manual identification, so we can only conclude that the most likely cause of this discrepancy is misidentification of expressing nuclei in the embryo by these two groups. **F**) Lineage diagram showing the overlapping and independent expression of three Wnt ligands in the comma-stage embryonic tail. Note that these lineages also express *cwn-2* and *mom-2* earlier in development.
(PNG)

S2 Fig Full lineages for nuclear β-catenin and POP-1 localization. **A**) Full β-catenin nuclear localization patterns for GFP::WRM-1 and Venus::SYS-1, data shown is an average of all lineages analyzed. **B**) Confocal plane showing embryonic localization of WRM-1::GFP. Although cytoplasmic expression is brighter than nuclear expression, our quantification approach accounts for this by subtracting local non-nuclear background. **C**) Average nuclear levels for mCherry::SYS-1 through the 350 cell stage. **D**) Confocal image of embryonic nuclear localization of mCherry::SYS-1. **E**) Nuclear localization patterns for GFP::POP-1, through the 350 cell stage (1 round of divisions less than A). Psys-1::GFP::POP-1 first becomes detectable at the 50 cell stage. **F**) Detail of the ABalaaaa lineage, showing that left-right divisions with strong asymmetry maintain the pattern of inverse correlation between nuclear POP-1 and β-catenin. The division of ABalaaaap (marked by brackets), produces two cells with symmetric expression of β-catenin and POP-1, note that nuclear β-catenin is low while POP-1 is high. **G**) Confocal image of embryonic localization of GFP::POP-1. **H**) Mean nuclear Venus levels for the Psys-1::Venus::SYS-1(stops) reporter in a *smg-1* mutant deficient in nonsense mediated decay. This reporter shows expression driven by the Psys-1 promoter, which is activated at the 50-cell stage and virtually ubiquitous and generally uniform. No expression is observed in the germ cells Z3 and Z3, and expression is delayed in the D lineage. Expression in the E lineage is weak with a posterior bias. This corresponds with lower nuclear localization of mCherry::SYS-1 and GFP::POP-1 in these lineages.
(PDF)

S3 Fig Cells that divide along the A-P axis with reversed polarity. **A**) ABalaaaar divides left-right across the midline to produce two daughters with no significant difference in nuclear GFP::WRM-1. B) The daughters of that cell (ABalaaaar(l/r)) divide to produce an “anterior” daughter that is skewed away from the midline and has high nuclear WRM-1, while the “posterior” midline-proximal daughter has low nuclear WRM-1. Several other l-r divisions in this lineage produce high nuclear WRM-1 in the daughter farther from the midline (dashed yellow line). C-E) Six divisions (3 L-R symmetric pairs) with clear A-P polarity reversals in late embryos. In each case the cell with higher nuclear β-catenin is denoted in red on the 3D projections. C’-E’) position cells that express the Wnt ligand cwn-2 (yellow) late in embryogenesis, relative to the position of the cells (pink), just before they divide. E”) rotated view of E’. Note that two of these (panels C and D) occur at adjacent positions but ~50 minutes apart suggesting that this position may have altered Wnt polarization relative to the rest of the embryos. E lineage (green) and ABplp (blue) and ABprp (purple) lineages are shown as positional references. These late reversed-polarity divisions (C-E) could reflect the development of non-posterior sources of Wnt in late embryogenesis, as occurs postembryonically in the developing vulva [54]. We do observed some more anterior cells that begin to express Wnt ligand later in development (C-E’), but it remains to be determined if they are influencing these reversals.
(PNG)

S4 Fig Impact of position on β-catenin asymmetry. **A-C**) Diagrams showing head-on views of the embryonic poles, with asymmetry of nuclear β-catenin shown (red = high, white = low). Organization of anterior pole (**A**) at the 600-cell stage. Organization of posterior pole at ~200-cell stage (**B**) and ~350-cell stage (**C**). At the 600-cell stage organization is similar to that in (**C**) except most C-lineage cells have not recently divided and no longer have nuclear β-catenin. Two of the eleven A-P divisions we observed with revered polarity occur near the anterior pole and are oriented similar to other L-R divisions nearby, suggesting the existence of a defined anterior organizing center for β-catenin asymmetry. Our work defines the positions of this and a presumptive posterior organizing center at the boundary between the ABpxpp and C lineages. At this Wnt signaling center the most posterior ABpxpp descendants polarize towards the C lineage descendants and vice versa. The mechanism by which these boundaries are robustly established and act to regulate polarity is an important ongoing question, but it suggests that in addition to patterning the anterior-posterior axis, Wnt signaling also establishes the medial-lateral axis at the poles. **D**) WRM-1 nuclear asymmetry for divisions as a function of position along the embryo’s anterior-posterior axis. Cells in the anterior of the embryo are more likely to have high levels of asymmetry. Cells below the axis correspond to l/r, d/v or reverse-polarity divisions in Fig 2D and 2E. **E**) WRM-1 nuclear asymmetry by founder lineage. The posterior and largely clonal E, C and D lineages have reduced levels of asymmetry. We observed that levels of nuclear β-catenin are correlated with fate, with higher nuclear WRM-1 concentrations and asymmetry in cells whose sister adopts a different fate compared with cells whose sister adopts the same fate (81% higher asymmetry; p<10–18). **F, G**) Even though nuclear β-catenin concentration increases over developmental time (**F**, SYS-1 shown), total levels of nuclear protein remain roughly constant over time (**G**).
(PNG)

S5 Fig Nuclear dynamics of Wnt-regulated proteins. Average nuclear enrichment across all cells across cellular lifespan for GFP::WRM-1 (**A**), Venus::SYS-1 (**B**), and across EMS-lineage cells for med-1p::GFP::POP-1 (**C**). Time scale is minutes after called division—the first two time points correspond roughly to anaphase and telophase. Error bars are SEM. Negative concentration (e.g. for anterior daughters nuclear GFP::WRM-1 levels) corresponds to nuclear concentration below the local cytoplasmic concentration.
(PNG)

S6 Fig POP-1 enrichment occurs in early EMS divisions and can be detected by antibody staining. The *med-1*::GFP::POP-1 transgene drives a pulse of POP-1 transcripts in the EMS cell and its daughters at the four and eight cell stages. These transcripts disappear by the 16-cell stage but the pulse of GFP::POP-1 protein persists for several additional divisions [36]. The GFP::POP-1 clearly has a bias towards nuclear localization anterior daughter cells (**A**). The round of divisions that generates 4 E cells are left-right (L-R), non-polarized divisions. There is further cousin enrichment for GFP::POP-1 in the POP-1 High-High cells (**B**). **C**) This results in a concentration gradient in which SYS-1 High-High (HH, red) cells have more nuclear SYS-1 and less POP-1 on average than their SYS-1 Low-High (LH, pink) cousins, and POP-1 High-High (HH, dark blue) cells have more nuclear POP-1 and less nuclear SYS-1 on average than their POP-1 Low-High (LH, light blue) cousins. These results are similar to those observed for the Psys-1::GFP::POP-1 transgene (Fig 3), which is expressed ubiquitously but only after the 50 cell stage. (**D-F**). Example of embryonic cell identification and POP-1 antibody quantificiation Quartets of cousin cells were identified by staining embryos carrying an UNC-130::GFP transgene for GFP (**D**). At the 350-cell stage, UNC-130 is expressed in six spatially-distinct quartets of cells. The general orientation of the embryo can be determined from the GFP-positive cells as well as the DAPI stain, such that each GFP-positive cell can be unambiguously identified. Embryos were co-stained with an anti-POP-1 antibody and labeled with a red fluorescent secondary (**E**). Within the nuclei defined by the GFP and DAPI, red intensity was quantified using ImageJ (**F**). For 60 quartets measured, the average POP-1 cousin enrichment is 1.31, while the average enrichment for the same lineages from our analysis of 8 GFP::POP-1 embryos is 1.27; the difference is not statistically significant (**G**).
(PNG)

S7 Fig Effects of other manipulations of Wnt signaling on β-catenin localization and transcription. **A**) WRM-1 nuclear localization decreases after RNAi against the Wnt ligand *mom-2* or the kinase *lit-1*, which prevents the nuclear export of TCF. Knockdown of these factors also decreases WRM-1 sister asymmetry, particularly for earlier divisions (**B**), similar to the effect on SYS-1 nuclear localization (Fig 4). **C**) Heat shock-inducible expression of the Wnt antagonist, *sfrp-1*, which is normally expressed only in the anterior of the embryo (40) causes increased incidences of reversed SYS-1 divisions (black arrows), SYS-1 equivalent divisions (white arrows) and decreased SYS-1 concentration (blue underlines). Heat shock to 32.5°C for 10 minutes was applied at the 28 cell stage for both transgenic and control embryos (black line). **D**) Quantification of all divisions after heat-shock shows statistically significant increases in total numbers of divisions displaying defective division orientation, no sister asymmetry and reversed asymmetry with overexpression of *sfrp-1*. Thresholds for defective division orientation and low SYS-1 asymmetry are set by the bottom 5 percentile of untreated wild-type divisions. Threshold for no asymmetry is set by the maximum asymmetry score for untreated wild-type divisions known to have no β-catenin asymmetry. Divisions are considered reversed if their asymmetry score is above the positive “no asymmetry” threshold. **E**) Average sister asymmetry is reduced after global mis-expression of *sfrp-1*, p = 0.14 pre-HS, p<10^−30^ post-HS). We also observed a small but statistically significant 20% decrease in average SYS-1 concentration post-HS (p<10^−23^). There was no difference in cousin enrichment with induced over-expression of sfrp-1. F) Global mis-expression of *sfrp-1* also causes reversals (black arrows) and decreased expression (blue underlines) of a synthetic TCF reporter transgene, *poptop* (see Fig 5), presumably because of the reversals and reduced concentration of SYS-1.
(PNG)

S8 Fig The effects of Wnt ligand and receptor knockdown on Venus::SYS-1 nuclear localization and cousin enrichment. **A**) Example trees showing nuclear localization of Venus::SYS-1 for the different Wnt ligand and receptor mutants. Reversed divisions (higher SYS-1 in the left, anterior daughter) are marked by black arrows. For untreated *cwn-1(ok546);cwn-2(ok895)*, the two of six embryos that showed reversed divisions are shown. Reductions in *mig-14* and *mom-2* cause an E—>MS fate transformation resulting in more mesoderm (MOM), orange underline. Note that color thresholds are different from Fig 4A. **B**) Box plot showing that the difference in SYS-1 sister asymmetry between cells with parents that did or did not express Wnt ligand observed in wildtype embryos is lost in mutant embryos that have severely reduced expression of Wnt ligands. **C**) Box plot showing the effect on SYS-1 cousin enrichment of different disruptions of sister asymmetry in *mom-5(or57)* mutant embryos. All are significantly different from wild-type (*). Green line indicates that when SYS-1 High-High and Low-High cousin nuclear localization is equal, cousin enrichment equals one. **D**) Average SYS-1 cousin enrichment is not significantly different from wild-type in any Wnt ligand mutants.
(PNG)

S9 Fig Role of the proteasome on POP-1/SYS-1 asymmetry and enrichment. **A**) Full lineages for Venus::SYS-1 treated with RNAi against *rpt-4* and *rsa-2*. Note: color threshold are different from Fig 4A to better show differences in nuclear localization later in development. **B**) RNAi against *rpt-4* causes an increase in cells with defective division orientations as well as low, no and reversed SYS-1 asymmetry. RNAi against *rsa-2* causes an increase in cells with defective division orientations and reversed SYS-1 asymmetry. Both are less severe than the Wnt pathway mutants in Fig 4F. **C**) RNAi against *rpt-4* causes decreased nuclear localization of WRM-1, as well as significantly reduced sister asymmetry (**D**) and cousin enrichment (**E**). This embryo displays a MOM (more mesoderm) phenotype, likely because the reduction in nuclear SYS-1 and WRM-1 in the E cell resulted in the failure of proper fate specification of the E lineage. **F**) RNAi against *rpt-4* has no significant effect on POP-1 nuclear localization, asymmetry (**G**) or cousin enrichment (**H**).
(PNG)

S10 Fig Full expression patterns of POP-1 targeted synthetic enhancers. **A**) POPTOP (7xTCF binding sites) expression is dependent on POP-1/TCF and SYS-1/β-catenin, and is partially inhibited by high nuclear concentrations of TCF that are caused by the knockdown of the kinase *lit-1*. **B**) Full lineages for the 6xTCF, 6x(TCF + Helper), 3xTCF and 1xTCF reporters. Several are shown with a lower threshold so that weaker expression is visible (arrows). Differences between the 6xTCF and 6x(TCF + Helper) reporters could be caused by several factors including the use of a different minimal promoter and 3’UTR, differences in the spacing of the binding sites, and integration site effects [65]). **C**) Physical locations of the cells expressing the 7x, 6x, 6x(TCF + Helper), and 3xTCF reporters. Non-expressing cells appear grey or clear, while weakly expressing cells are black and strongly expressing cells are bright red. Note that high expressing cells are found in the anterior half of the embryo, even though their relative position to other related cells is posterior.
(PNG)

S11 Fig Expression patterns for all posteriorly-expressed target genes in wild-type embryos and after RNAi. The MS (pink) and E (orange) lineages are labeled and underlined for all genes to highlight the MS—>E fate transformation with *pop-1* RNAi and the E—>MS fate transformation with lit-1 RNAi. Additional expressing lineages are also labeled and underlined. Expression was considered to be strongly activated by *pop-1/sys-1* if expression was completely lost with both *pop-1* and *sys-1* RNAi; moderately activated if completely lost with one and reduced in the other, and weakly activated if expression was reduced or delayed with *pop-1* and *sys-1* RNAi. Expression was considered to be strongly repressed if *pop-1* RNAi caused broadly expanded expression at roughly the same level as wild type, moderately repressed if *pop-1* RNAi caused broadly expanded expression at a level less than wildtype and weakly repressed if expression was narrowly expanded at a level less than wildtype. No change was considered unregulated. **A**) Expression of CEH-6::GFP is lost or reduced in most expressing lineages after *pop-1*, *sys-1* or *lit-1* RNAi. **B**) Expression of CEH-13::GFP is expanded in ABalap (white asterisk) after *pop-1* RNAi because of a known fate transformation in which ABala adopts the fate of ABarp (white asterisk)[14]. Since expression in ABarpp is largely unchanged in both *pop-1* and *sys-1* RNAi, we conclude that *ceh-13* is unregulated by *pop-1/sys-1* in ABa (purple underline). Expression in ABp (black underline) is significantly decreased with *pop-1*, *sys-1* and *lit-1* RNAi. Note that the broad, transient early expression is more affected than the later stronger expression limited to a few branches. **C**) Expression of the *ceh-27* promoter is expanded in ABa with *lit-1* RNAi, which is inconsistent with direct regulation. Expression of the *ceh-27* promoter is expanded in ABp with *pop-1* RNAi, and ABp expression is largely unchanged with *sys-1* RNAi and lost with *lit-1* RNAi. **D**) Expression of the *ceh-36* promoter is lost with *pop-1*, *sys-1* and *lit-1* RNAi, while broad expansion is observed in ABp with *pop-1* RNAi and no change in *sys-1* RNAi. Expression in lost in MS with *pop-1* RNAi due to a known MS—>E fate switch [14], but unchanged with *sys-1* or *lit-1* RNAi. **E**) Expression of the *ceh-43* promoter is expanded with *lit-1* RNAi, which is inconsistent with direct regulation by TCF. **F**) The *cwn-1* promoter is only expressed in the C and D lineages, so partial editing is shown for other lineages. Expression of the *cwn-1* promoter is lost or reduced with *pop-1*, *sys-1* and *lit-*1 RNAi. **G**) Expression of the *cwn-2* promoter in ABa and E is lost or reduced with *pop-1*, *sys-1* and *lit-1* RNAi. Expression of the *cwn-2* promoter in ABp is expanded with *pop-1* RNAi, lost with *lit-1* RNAi and largely unchanged with *sys-1* RNAi. **H**) Expression of the *elt-6* promoter is lost or reduced with *pop-1*, *sys-1* and *lit-1* RNAi. **I, J**) Expression of the promoters of the known POP-1 targets, *end-1* (**I**) and *end-3* (**J**), becomes reduced in E and increased in MS, resulting in roughly equal expression with *pop-1* RNAi. Expression is lost with *lit-1* RNAi. Note: The result for *end-3* contradicts the result from Robertson et al., 2014 in which deletion of predicted TCF binding sites from a 1.3 kb end-3 promoter has no effect on expression, leading to their conclusion that it is not regulated by POP-1 [84]. Our promoter contains an additional 1 kb of sequence; a POP-1 regulated region could be found in this region, that POP-1 may act through non-canonical sites to regulate end-3 expression, or differences in the the quantification methods may explain this discrepancy. **K**) Expression of ETS-7::GFP protein is lost or reduced with *pop-1*, *sys-1* and *lit-1* RNAi. **L**) Expression of the *mir-57* promoter is lost, reduced or delayed with *pop-1*, *sys-1* and *lit-1* RNAi. Since *mir-57* is not expressed in ABa, some lineages were only partially edited. **M**) Expression of the *mom-2* promoter is virtually unchanged with *pop-1*, *sys-1* and *lit-1* RNAi, so it is considered to be unregulated by *pop-1*. **N**) Expression of NHR-25::GFP is lost or reduced in ABa and ABp with *pop-1*, *sys-1* and *lit-1* RNAi, but largely unchanged in the C lineage. Expression in ABa is 51% lower than wild type with *sys-1* RNAi. **O**) Expression of the *nhr-67* promoter is quantitatively decreased in the ABp cells which normally express it with *pop-1* RNAi, but also expanded into ABpla and ABpra due to a known fate change caused by reduced *pop-1* (blue arrows) [14]. Since ABp expression is lost in ABp with both *sys-1* and *lit-1* RNAi, we conclude that nhr-67 requires *pop-1/sys-1* primarily for activation in ABp. Expression in MS is lost or reduced with *pop-1*, *sys-1* and *lit-1* RNAi. **P**) Expression of the *nob-1* promoter in the ABp lineage is lost or reduced with *pop-1*, *sys-1* and *lit-1* RNAi, but expression in the E lineage is largely unchanged. **Q**) Expression of PAL-1::GFP is lost or reduced in the ABa, ABp, and D lineages with *pop-1*, *sys-1* and *lit-1* RNAi. Expression in the C lineage is strongest in posterior branches of the C lineage in wildtype and expression in C becomes uniform with pop-1 RNAi and reduced with *sys-1* and *lit-1* RNAi. **R**) Expression of the *pax-3* promoter is lost, reduced or delayed with *pop-1*, *sys-1* and *lit-1* RNAi. Since expression of *pax-3* is limited to ABp, some non-expressing lineages are only partially edited. **S**) Expression of the *sem-2* promoter is expressed in the ABa, ABp, MS and E lineages. Expression is expanded in ABa and ABp with *pop-1* RNAi, unchanged with *sys-1* RNAi and lost with *lit-1* RNAi. **T**) Expression of the *tbx-11* promoter is expanded in the ABa, MS, E, C and D lineages with *pop-1* RNAi. Expression is largely unchanged with *sys-1* RNAi, but reduced with *lit-1* RNAi. Expression in ABp is largely unchanged with *pop-1* and *sys-1* RNAi and slightly reduced with *lit-1* RNAi. **U)** Expression of the *tlp-1* promoter is increased in ABa and ABp with *pop-1* RNAi, largely unchanged with *sys-1* RNAi and decreased with *lit-1* RNAi. Expression in C and E is unaffected by *pop-1* RNAi. **V**) Expression of UNC-30::GFP-tagged protein is expanded with *pop-1* RNAi, unchanged with *sys-1* RNAi and decreased with *lit-1* RNAi. **W**) Expression of the UNC-130::GFP is lost, reduced with *pop-1*, *sys-1* and *lit-1* RNAi. Since expression of *unc-130* is limited to ABp, some non-expressing lineages are only partially edited. **X**) Expression of the *vab-7* promoter is lost, reduced with *pop-1*, *sys-1* and *lit-1* RNAi. Since expression of *vab-7* is limited to C, some non-expressing lineages are only partially edited.
(PDF)
