# Supplementary figures and images for: Quantitative Differences in Nuclear β-catenin and TCF Pattern Embryonic Cells in C. elegans
Source: PLoS Genet. 2015 Oct 21;11(10):e1005585. doi: 10.1371/journal.pgen.1005585 (PMC4619327; doi:10.1371/journal.pgen.1005585)

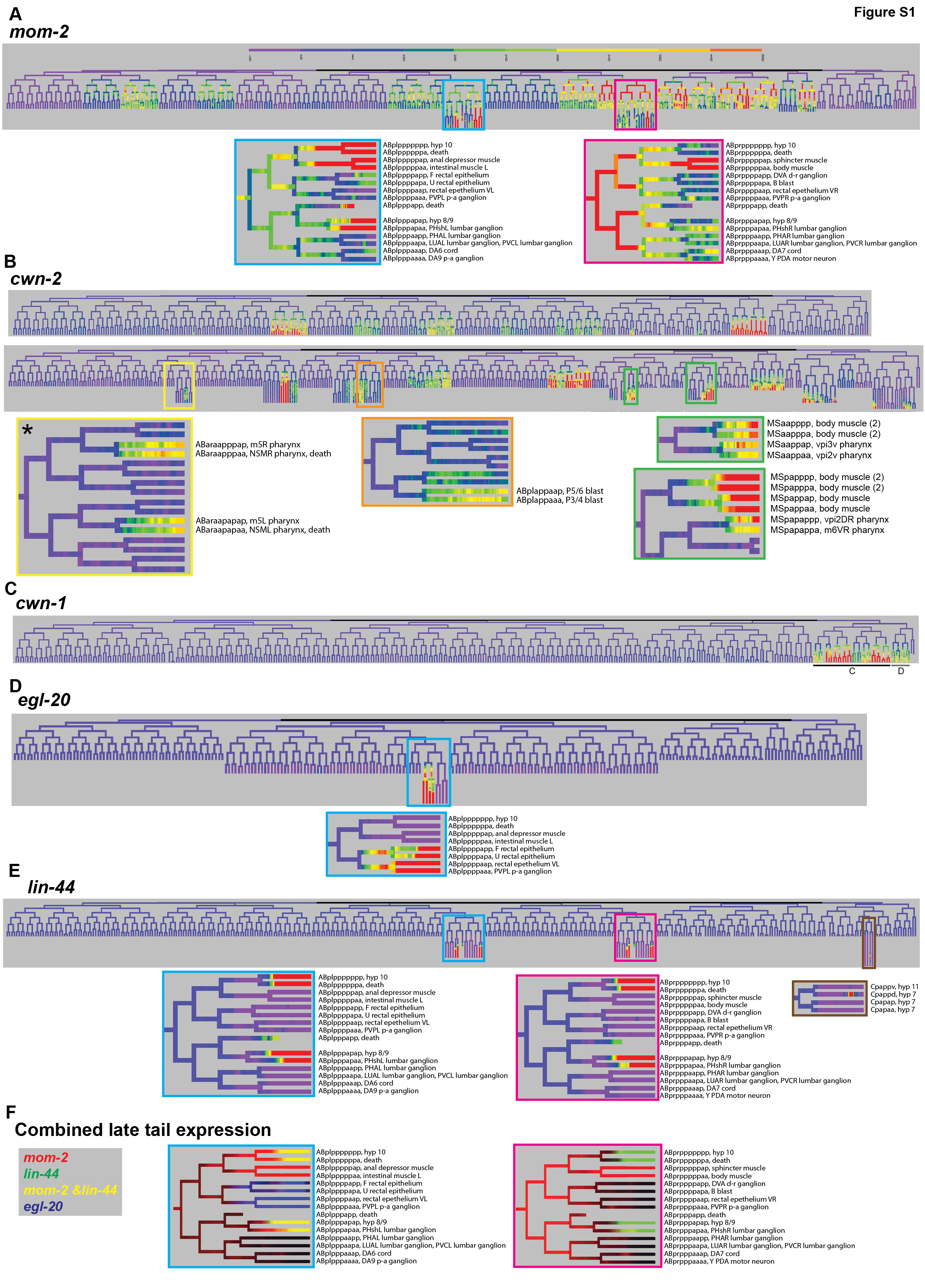

Supplement: S1 Fig — A) We observed dynamic expression of the mom-2 promoter, although maternal expression was undetectable. Early in development, weak expression was observed in ABpra; moderate expression in ABalp, ABarap; strong expression in ABplp, ABprp, MS, and E. Expression is constant or decays in daughter cells after roughly the 50 cell stage, suggesting the promoter is no longer active. Expression is re-activated in ABplpppapaa, (PHshL lumbar ganglion), ABplpppapap (hyp 8/9), ABplpppppaa (intestinal muscle L), ABplppppppa (death), ABplppppppp (hyp 10), ABplpppppaa (body muscle), and ABplpppppap (sphincter muscle). B) We observed variable expression of cwn-2, likely due to the nature of the extrachromasomal array, which can be silenced or lost during mitosis. Two separate lineages (and part of a third marked by an asterisk) are shown to display the variability. We observed early expression in ABarpp, ABplap, ABplpp, ABprpp, and E. Late activation of expression is also observed in the derivatives of MSaapp and MSpapp, ABaraapapaa (NSML pharynx, death), ABaraapapap (m5L pharynx), ABaraapppaa (NSML pharynx, death), ABaraapppap (m5L pharynx), ABplappaaa (P3/4 blast), and ABplappaap (P5/6 blast). C) We observed expression of cwn-1 in all cells of the C lineage and variable expression in the D lineage, with stronger expression in derivatives of Caap, Cap, and Cpp. D) We observed expression driven by the egl-20 promoter in the sister cells ABplppppaa and ABplppppap at the 350 cell stage and strong expression in their daughter cells: ABplppppaaa, the PVPL interneuron; ABplppppaap, the VL cell of the rectal gland; ABplppppapa, the U rectal epithelial cell; and ABplppppapp, the K rectal epithelial cell. We did not observe embryonic expression through the 1.5 fold stage in the p9/10 or p11/12 seam cells (ABplapapap, ABplapappa), the B rectal epithelial cell (ABprppppapa), or the anal depressor muscle (ABplpppppap), in which expression was reported at the L1 larval stage [41]. Expression in [file pgen.1005585.s006.png]

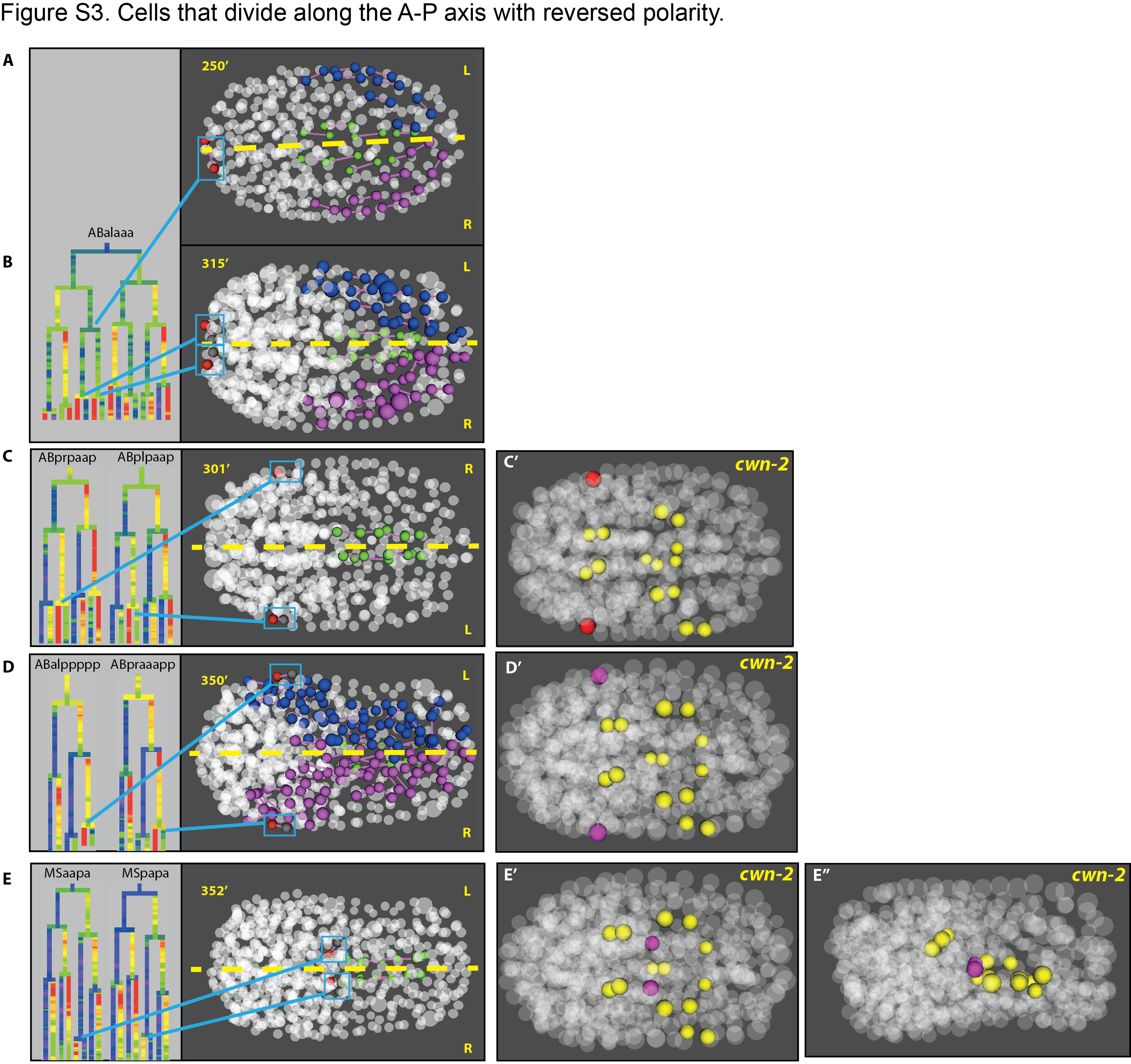

Supplement: S3 Fig — A) ABalaaaar divides left-right across the midline to produce two daughters with no significant difference in nuclear GFP::WRM-1. B) The daughters of that cell (ABalaaaar(l/r)) divide to produce an “anterior” daughter that is skewed away from the midline and has high nuclear WRM-1, while the “posterior” midline-proximal daughter has low nuclear WRM-1. Several other l-r divisions in this lineage produce high nuclear WRM-1 in the daughter farther from the midline (dashed yellow line). C-E) Six divisions (3 L-R symmetric pairs) with clear A-P polarity reversals in late embryos. In each case the cell with higher nuclear β-catenin is denoted in red on the 3D projections. C’-E’) position cells that express the Wnt ligand cwn-2 (yellow) late in embryogenesis, relative to the position of the cells (pink), just before they divide. E”) rotated view of E’. Note that two of these (panels C and D) occur at adjacent positions but ~50 minutes apart suggesting that this position may have altered Wnt polarization relative to the rest of the embryos. E lineage (green) and ABplp (blue) and ABprp (purple) lineages are shown as positional references. These late reversed-polarity divisions (C-E) could reflect the development of non-posterior sources of Wnt in late embryogenesis, as occurs postembryonically in the developing vulva [54]. We do observed some more anterior cells that begin to express Wnt ligand later in development (C-E’), but it remains to be determined if they are influencing these reversals. (PNG) [file pgen.1005585.s008.png]

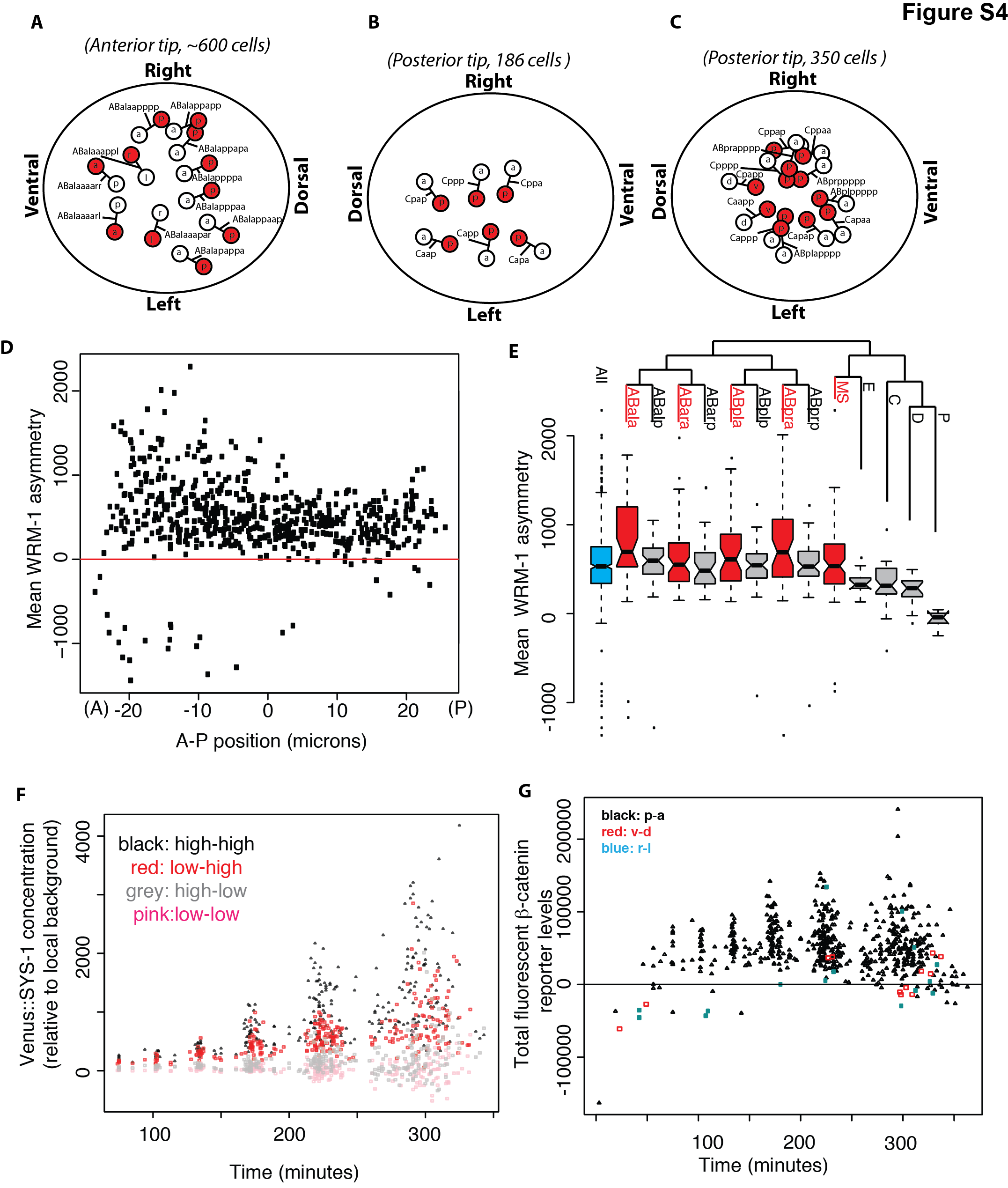

Supplement: S4 Fig — A-C) Diagrams showing head-on views of the embryonic poles, with asymmetry of nuclear β-catenin shown (red = high, white = low). Organization of anterior pole (A) at the 600-cell stage. Organization of posterior pole at ~200-cell stage (B) and ~350-cell stage (C). At the 600-cell stage organization is similar to that in (C) except most C-lineage cells have not recently divided and no longer have nuclear β-catenin. Two of the eleven A-P divisions we observed with revered polarity occur near the anterior pole and are oriented similar to other L-R divisions nearby, suggesting the existence of a defined anterior organizing center for β-catenin asymmetry. Our work defines the positions of this and a presumptive posterior organizing center at the boundary between the ABpxpp and C lineages. At this Wnt signaling center the most posterior ABpxpp descendants polarize towards the C lineage descendants and vice versa. The mechanism by which these boundaries are robustly established and act to regulate polarity is an important ongoing question, but it suggests that in addition to patterning the anterior-posterior axis, Wnt signaling also establishes the medial-lateral axis at the poles. D) WRM-1 nuclear asymmetry for divisions as a function of position along the embryo’s anterior-posterior axis. Cells in the anterior of the embryo are more likely to have high levels of asymmetry. Cells below the axis correspond to l/r, d/v or reverse-polarity divisions in Fig 2D and 2E. E) WRM-1 nuclear asymmetry by founder lineage. The posterior and largely clonal E, C and D lineages have reduced levels of asymmetry. We observed that levels of nuclear β-catenin are correlated with fate, with higher nuclear WRM-1 concentrations and asymmetry in cells whose sister adopts a different fate compared with cells whose sister adopts the same fate (81% higher asymmetry; p<10–18). F, G) Even though nuclear β-catenin concentration increases over developmental time (F, SYS-1 shown), total levels of nuclea [file pgen.1005585.s009.png]

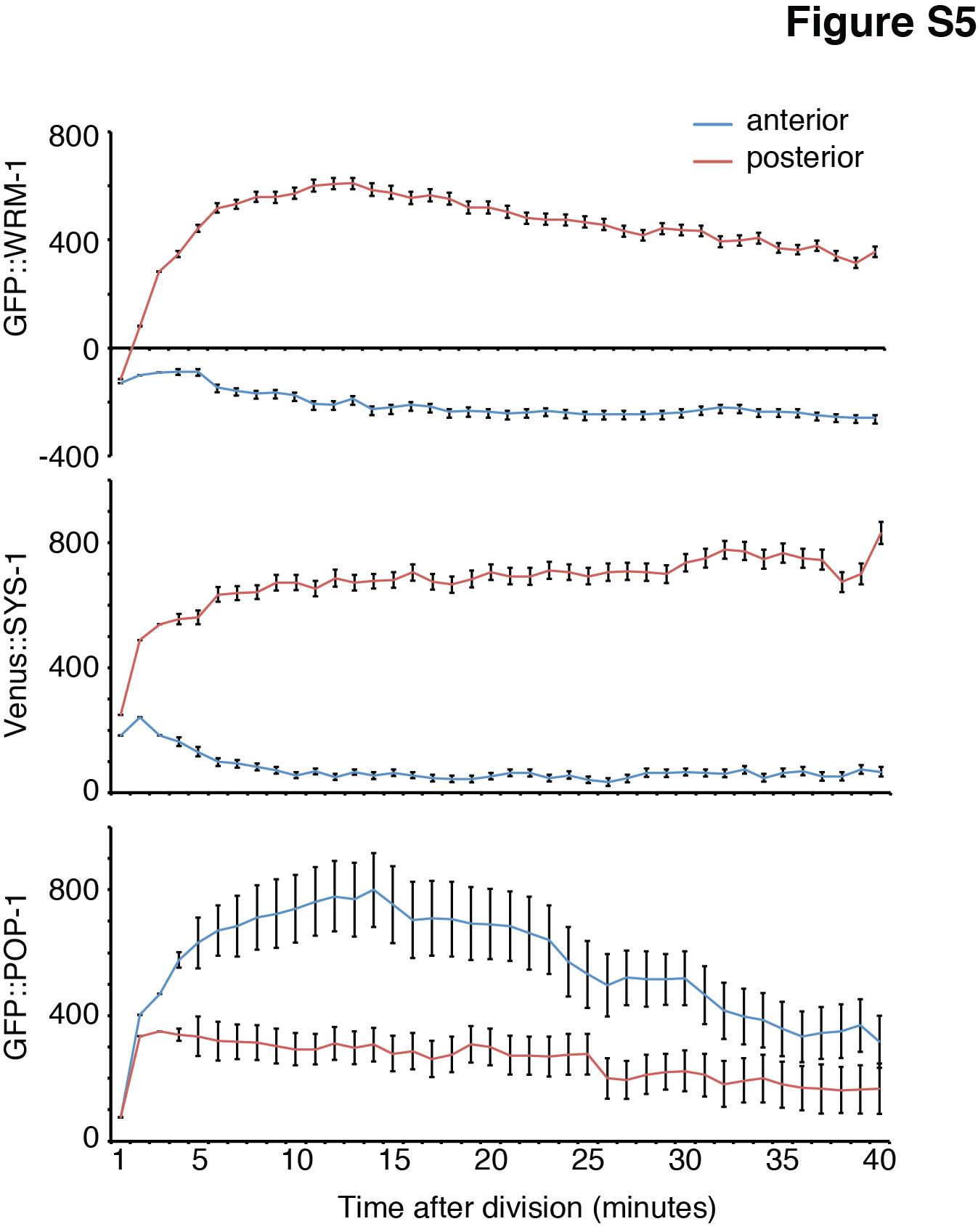

Supplement: S5 Fig — Average nuclear enrichment across all cells across cellular lifespan for GFP::WRM-1 (A), Venus::SYS-1 (B), and across EMS-lineage cells for med-1p::GFP::POP-1 (C). Time scale is minutes after called division—the first two time points correspond roughly to anaphase and telophase. Error bars are SEM. Negative concentration (e.g. for anterior daughters nuclear GFP::WRM-1 levels) corresponds to nuclear concentration below the local cytoplasmic concentration. (PNG) [file pgen.1005585.s010.png]

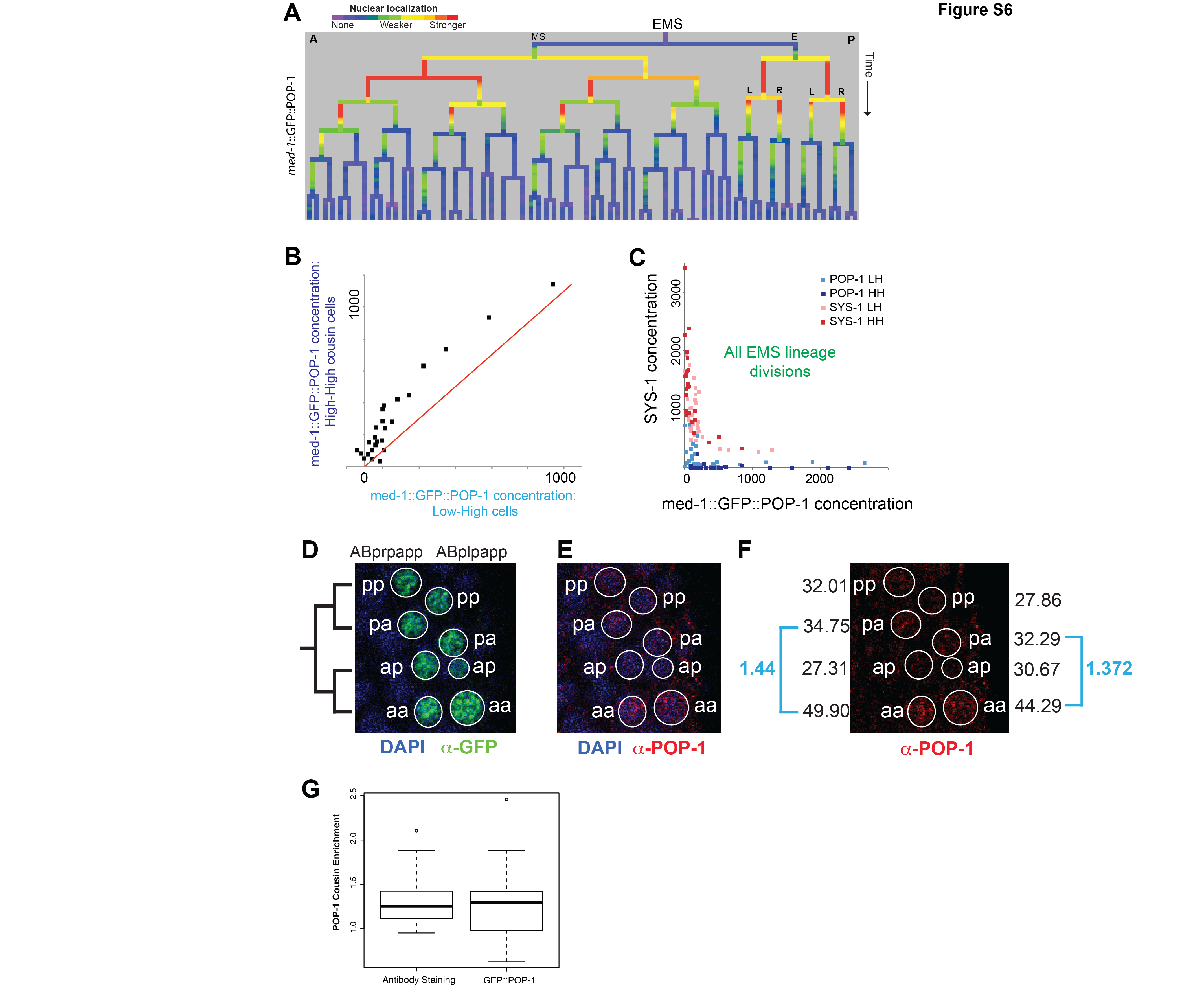

Supplement: S6 Fig — The med-1::GFP::POP-1 transgene drives a pulse of POP-1 transcripts in the EMS cell and its daughters at the four and eight cell stages. These transcripts disappear by the 16-cell stage but the pulse of GFP::POP-1 protein persists for several additional divisions [36]. The GFP::POP-1 clearly has a bias towards nuclear localization anterior daughter cells (A). The round of divisions that generates 4 E cells are left-right (L-R), non-polarized divisions. There is further cousin enrichment for GFP::POP-1 in the POP-1 High-High cells (B). C) This results in a concentration gradient in which SYS-1 High-High (HH, red) cells have more nuclear SYS-1 and less POP-1 on average than their SYS-1 Low-High (LH, pink) cousins, and POP-1 High-High (HH, dark blue) cells have more nuclear POP-1 and less nuclear SYS-1 on average than their POP-1 Low-High (LH, light blue) cousins. These results are similar to those observed for the Psys-1::GFP::POP-1 transgene (Fig 3), which is expressed ubiquitously but only after the 50 cell stage. (D-F). Example of embryonic cell identification and POP-1 antibody quantificiation Quartets of cousin cells were identified by staining embryos carrying an UNC-130::GFP transgene for GFP (D). At the 350-cell stage, UNC-130 is expressed in six spatially-distinct quartets of cells. The general orientation of the embryo can be determined from the GFP-positive cells as well as the DAPI stain, such that each GFP-positive cell can be unambiguously identified. Embryos were co-stained with an anti-POP-1 antibody and labeled with a red fluorescent secondary (E). Within the nuclei defined by the GFP and DAPI, red intensity was quantified using ImageJ (F). For 60 quartets measured, the average POP-1 cousin enrichment is 1.31, while the average enrichment for the same lineages from our analysis of 8 GFP::POP-1 embryos is 1.27; the difference is not statistically significant (G). (PNG) [file pgen.1005585.s011.png]

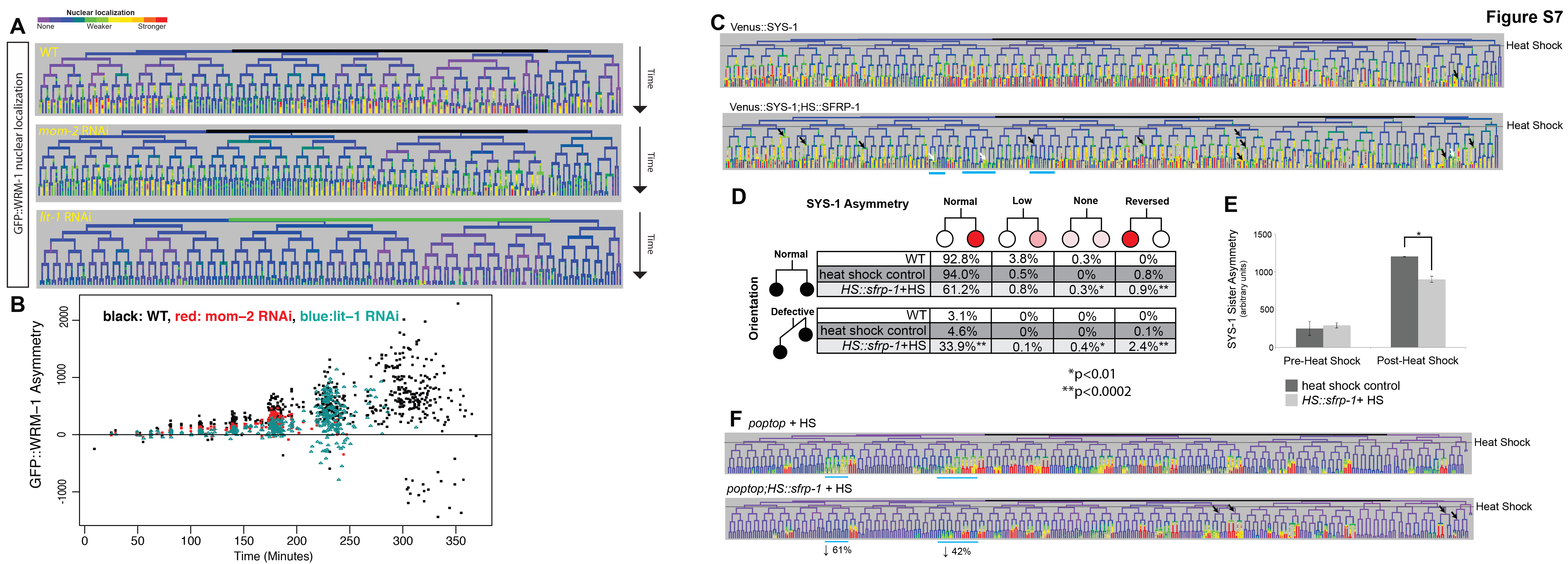

Supplement: S7 Fig — A) WRM-1 nuclear localization decreases after RNAi against the Wnt ligand mom-2 or the kinase lit-1, which prevents the nuclear export of TCF. Knockdown of these factors also decreases WRM-1 sister asymmetry, particularly for earlier divisions (B), similar to the effect on SYS-1 nuclear localization (Fig 4). C) Heat shock-inducible expression of the Wnt antagonist, sfrp-1, which is normally expressed only in the anterior of the embryo (40) causes increased incidences of reversed SYS-1 divisions (black arrows), SYS-1 equivalent divisions (white arrows) and decreased SYS-1 concentration (blue underlines). Heat shock to 32.5°C for 10 minutes was applied at the 28 cell stage for both transgenic and control embryos (black line). D) Quantification of all divisions after heat-shock shows statistically significant increases in total numbers of divisions displaying defective division orientation, no sister asymmetry and reversed asymmetry with overexpression of sfrp-1. Thresholds for defective division orientation and low SYS-1 asymmetry are set by the bottom 5 percentile of untreated wild-type divisions. Threshold for no asymmetry is set by the maximum asymmetry score for untreated wild-type divisions known to have no β-catenin asymmetry. Divisions are considered reversed if their asymmetry score is above the positive “no asymmetry” threshold. E) Average sister asymmetry is reduced after global mis-expression of sfrp-1, p = 0.14 pre-HS, p<10−30 post-HS). We also observed a small but statistically significant 20% decrease in average SYS-1 concentration post-HS (p<10−23). There was no difference in cousin enrichment with induced over-expression of sfrp-1. F) Global mis-expression of sfrp-1 also causes reversals (black arrows) and decreased expression (blue underlines) of a synthetic TCF reporter transgene, poptop (see Fig 5), presumably because of the reversals and reduced concentration of SYS-1. (PNG) [file pgen.1005585.s012.png]

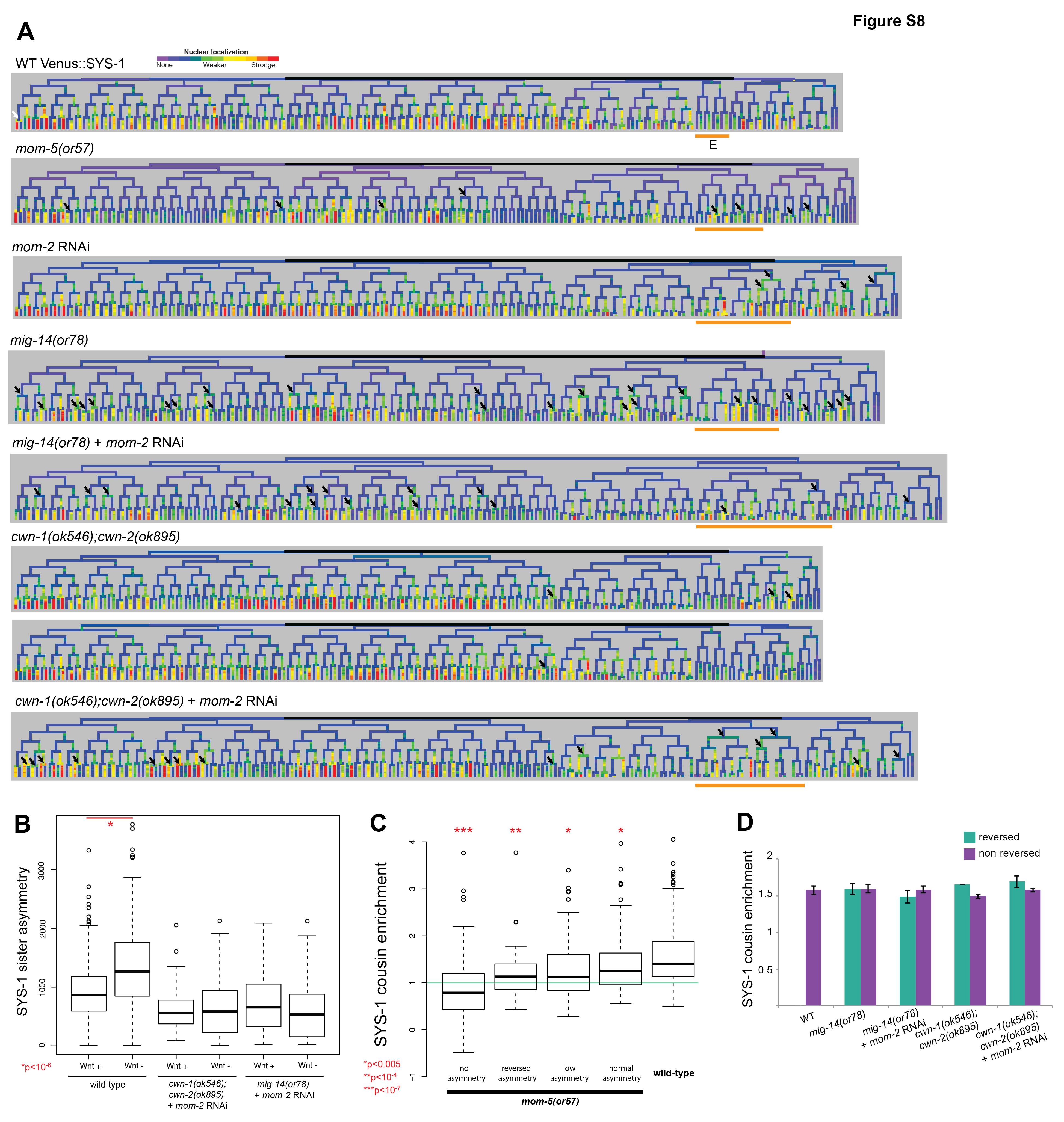

Supplement: S8 Fig — A) Example trees showing nuclear localization of Venus::SYS-1 for the different Wnt ligand and receptor mutants. Reversed divisions (higher SYS-1 in the left, anterior daughter) are marked by black arrows. For untreated cwn-1(ok546);cwn-2(ok895), the two of six embryos that showed reversed divisions are shown. Reductions in mig-14 and mom-2 cause an E—>MS fate transformation resulting in more mesoderm (MOM), orange underline. Note that color thresholds are different from Fig 4A. B) Box plot showing that the difference in SYS-1 sister asymmetry between cells with parents that did or did not express Wnt ligand observed in wildtype embryos is lost in mutant embryos that have severely reduced expression of Wnt ligands. C) Box plot showing the effect on SYS-1 cousin enrichment of different disruptions of sister asymmetry in mom-5(or57) mutant embryos. All are significantly different from wild-type (*). Green line indicates that when SYS-1 High-High and Low-High cousin nuclear localization is equal, cousin enrichment equals one. D) Average SYS-1 cousin enrichment is not significantly different from wild-type in any Wnt ligand mutants. (PNG) [file pgen.1005585.s013.png]

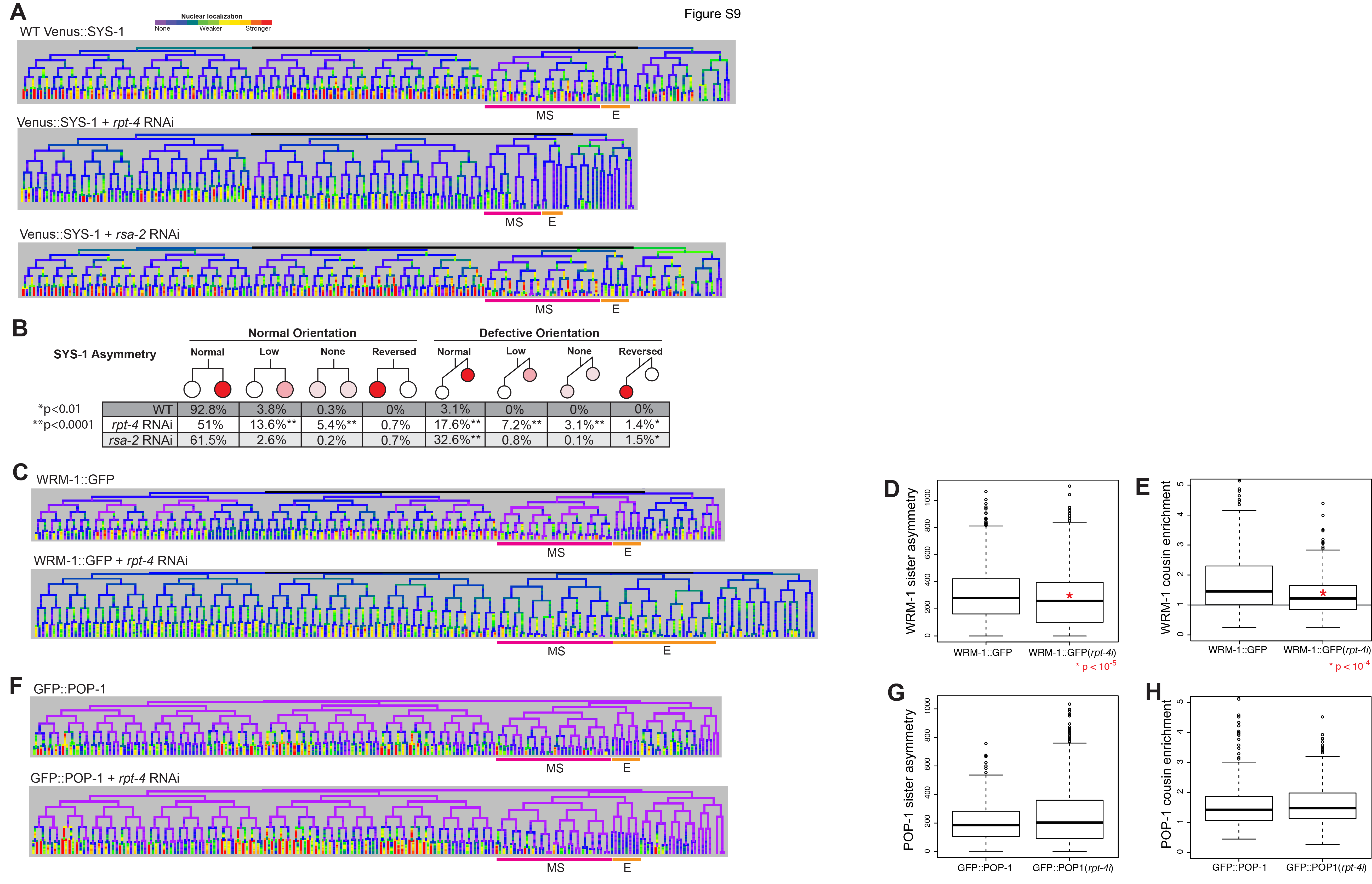

Supplement: S9 Fig — A) Full lineages for Venus::SYS-1 treated with RNAi against rpt-4 and rsa-2. Note: color threshold are different from Fig 4A to better show differences in nuclear localization later in development. B) RNAi against rpt-4 causes an increase in cells with defective division orientations as well as low, no and reversed SYS-1 asymmetry. RNAi against rsa-2 causes an increase in cells with defective division orientations and reversed SYS-1 asymmetry. Both are less severe than the Wnt pathway mutants in Fig 4F. C) RNAi against rpt-4 causes decreased nuclear localization of WRM-1, as well as significantly reduced sister asymmetry (D) and cousin enrichment (E). This embryo displays a MOM (more mesoderm) phenotype, likely because the reduction in nuclear SYS-1 and WRM-1 in the E cell resulted in the failure of proper fate specification of the E lineage. F) RNAi against rpt-4 has no significant effect on POP-1 nuclear localization, asymmetry (G) or cousin enrichment (H). (PNG) [file pgen.1005585.s014.png]

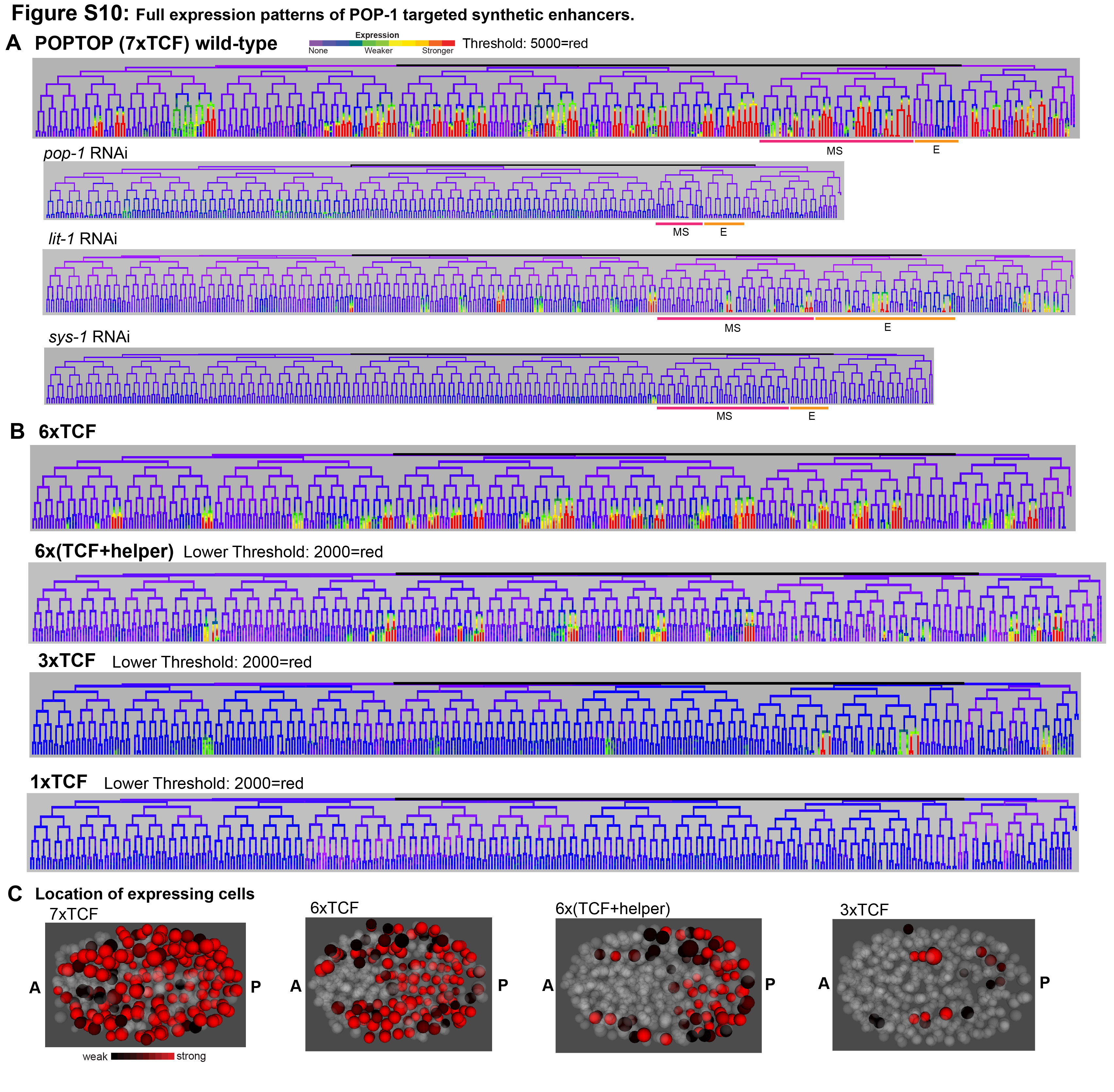

Supplement: S10 Fig — A) POPTOP (7xTCF binding sites) expression is dependent on POP-1/TCF and SYS-1/β-catenin, and is partially inhibited by high nuclear concentrations of TCF that are caused by the knockdown of the kinase lit-1. B) Full lineages for the 6xTCF, 6x(TCF + Helper), 3xTCF and 1xTCF reporters. Several are shown with a lower threshold so that weaker expression is visible (arrows). Differences between the 6xTCF and 6x(TCF + Helper) reporters could be caused by several factors including the use of a different minimal promoter and 3’UTR, differences in the spacing of the binding sites, and integration site effects [65]). C) Physical locations of the cells expressing the 7x, 6x, 6x(TCF + Helper), and 3xTCF reporters. Non-expressing cells appear grey or clear, while weakly expressing cells are black and strongly expressing cells are bright red. Note that high expressing cells are found in the anterior half of the embryo, even though their relative position to other related cells is posterior. (PNG) [file pgen.1005585.s015.png]
