# Supplementary material for: Quantitative Differences in Nuclear β-catenin and TCF Pattern Embryonic Cells in C. elegans
Source: PLoS Genet. 2015 Oct 21;11(10):e1005585. doi: 10.1371/journal.pgen.1005585 (PMC4619327; doi:10.1371/journal.pgen.1005585)

Figure S2: Full lineages for nuclear β-catenin and POP-1 localization

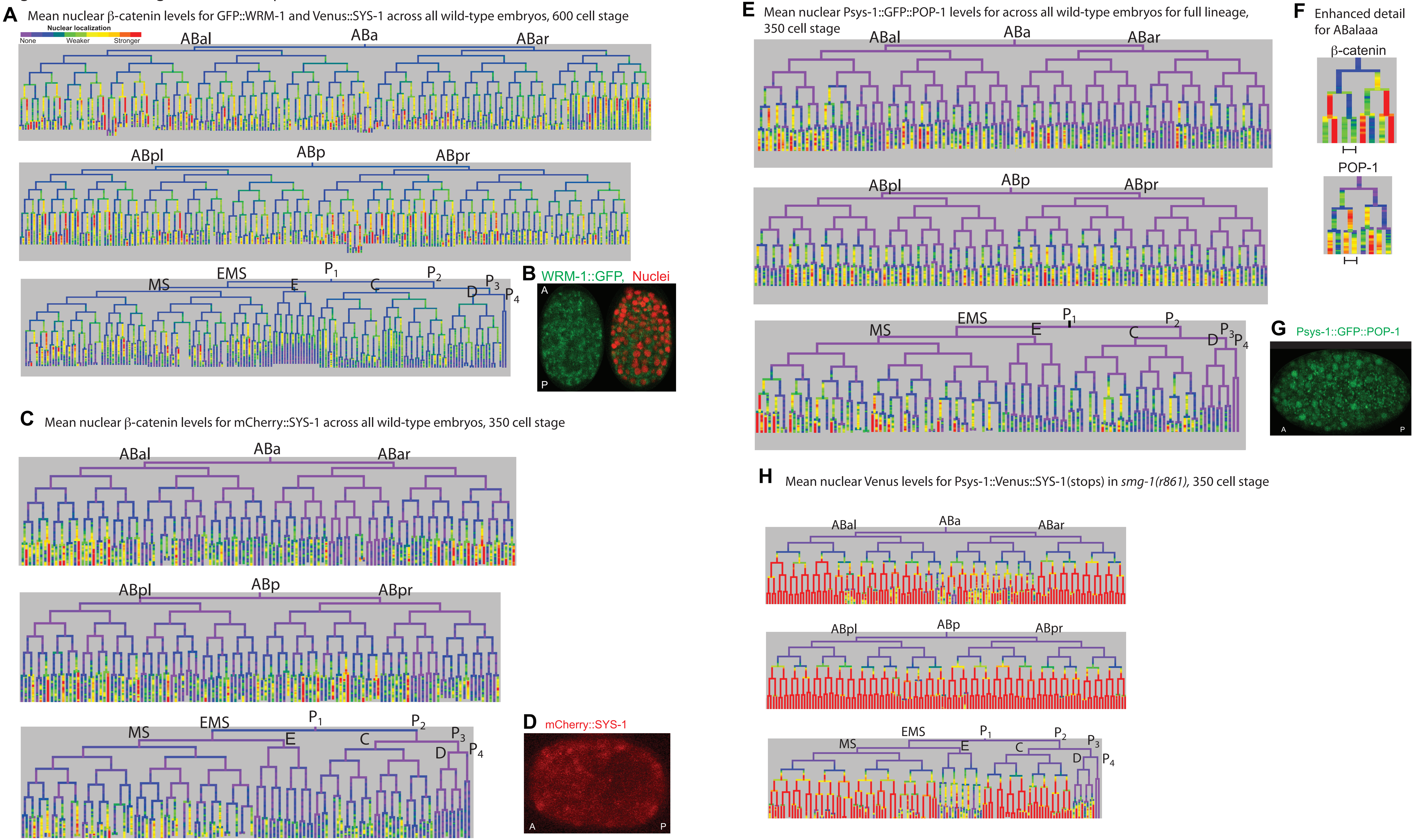

Supplement: S2 Fig — A) Full β-catenin nuclear localization patterns for GFP::WRM-1 and Venus::SYS-1, data shown is an average of all lineages analyzed. B) Confocal plane showing embryonic localization of WRM-1::GFP. Although cytoplasmic expression is brighter than nuclear expression, our quantification approach accounts for this by subtracting local non-nuclear background. C) Average nuclear levels for mCherry::SYS-1 through the 350 cell stage. D) Confocal image of embryonic nuclear localization of mCherry::SYS-1. E) Nuclear localization patterns for GFP::POP-1, through the 350 cell stage (1 round of divisions less than A). Psys-1::GFP::POP-1 first becomes detectable at the 50 cell stage. F) Detail of the ABalaaaa lineage, showing that left-right divisions with strong asymmetry maintain the pattern of inverse correlation between nuclear POP-1 and β-catenin. The division of ABalaaaap (marked by brackets), produces two cells with symmetric expression of β-catenin and POP-1, note that nuclear β-catenin is low while POP-1 is high. G) Confocal image of embryonic localization of GFP::POP-1. H) Mean nuclear Venus levels for the Psys-1::Venus::SYS-1(stops) reporter in a smg-1 mutant deficient in nonsense mediated decay. This reporter shows expression driven by the Psys-1 promoter, which is activated at the 50-cell stage and virtually ubiquitous and generally uniform. No expression is observed in the germ cells Z3 and Z3, and expression is delayed in the D lineage. Expression in the E lineage is weak with a posterior bias. This corresponds with lower nuclear localization of mCherry::SYS-1 and GFP::POP-1 in these lineages. (PDF) [file pgen.1005585.s007.pdf]
