# Supplementary material for: Quantitative Differences in Nuclear β-catenin and TCF Pattern Embryonic Cells in C. elegans
Source: PLoS Genet. 2015 Oct 21;11(10):e1005585. doi: 10.1371/journal.pgen.1005585 (PMC4619327; doi:10.1371/journal.pgen.1005585)

Figure S11: Expression patterns for all posteriorly-expressed target genes in wildtype embryos and after RNAi.

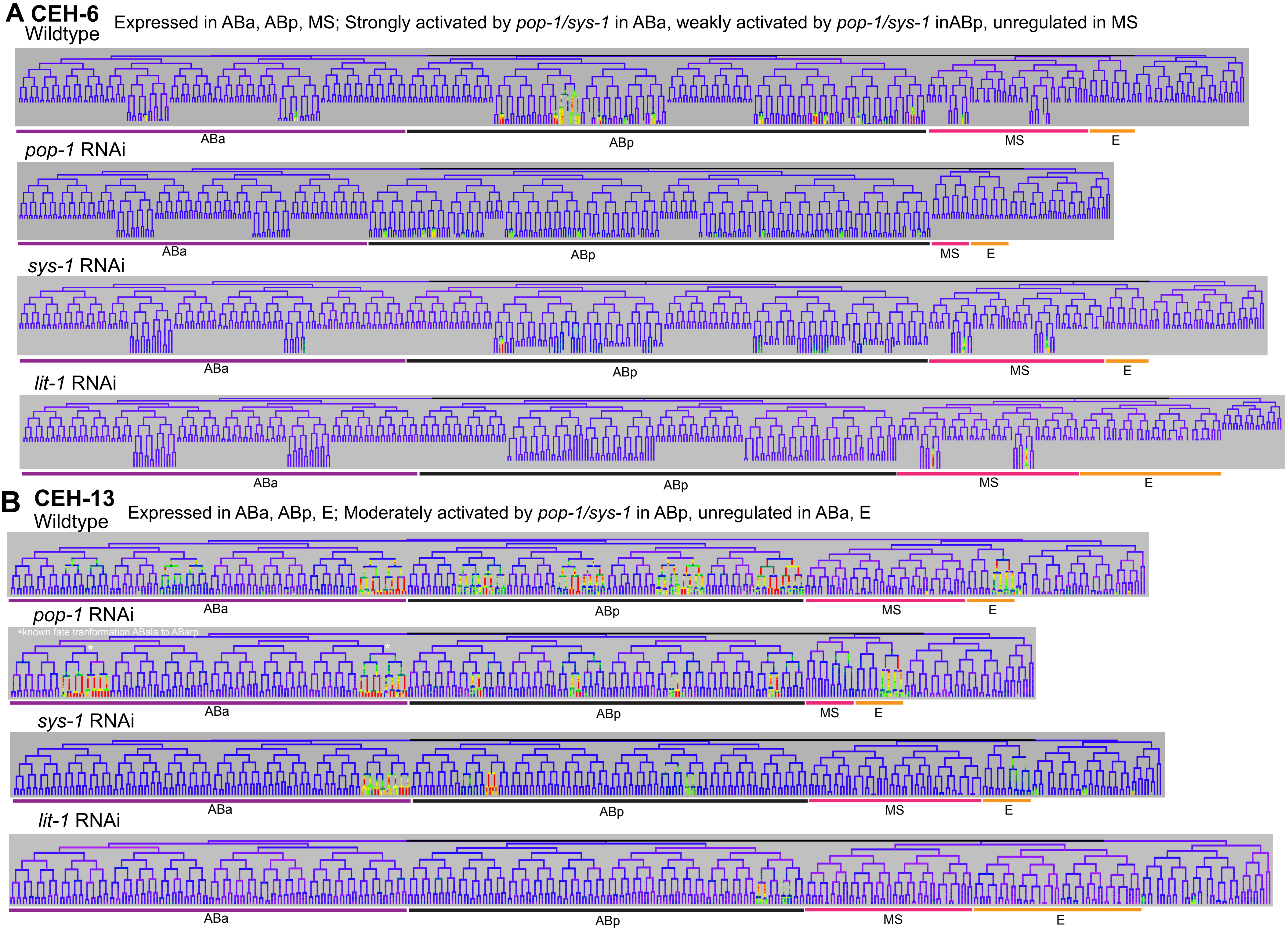

Figure S11C

CEH-27 Expressed in ABa, ABp; Indirectly regulated by *pop-1* in ABa, Moderately repressed by *pop-1* in ABp,

Wildtype

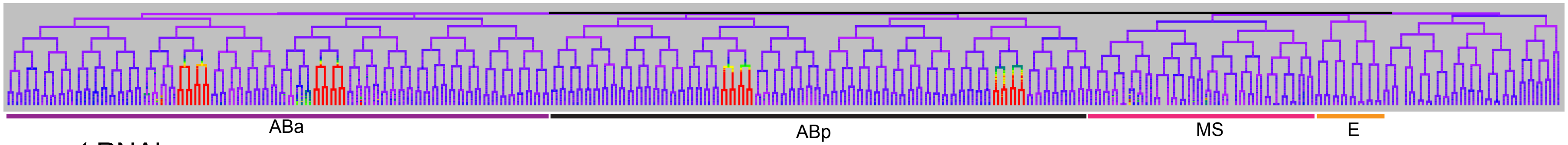

pop-1 RNAi

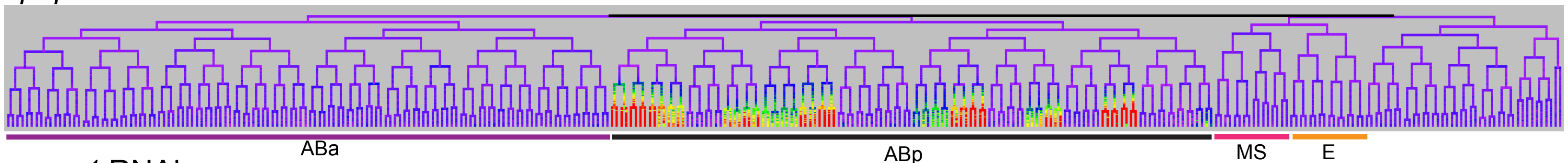

sys-1 RNAi

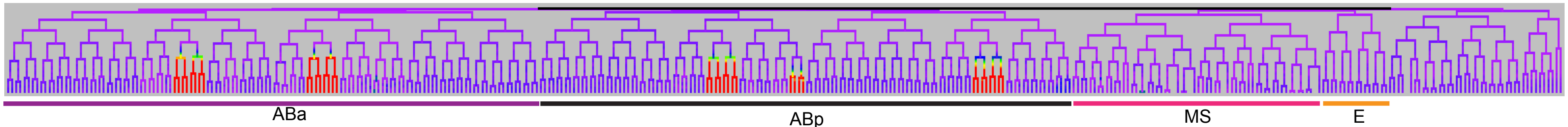

lit-1 RNAi

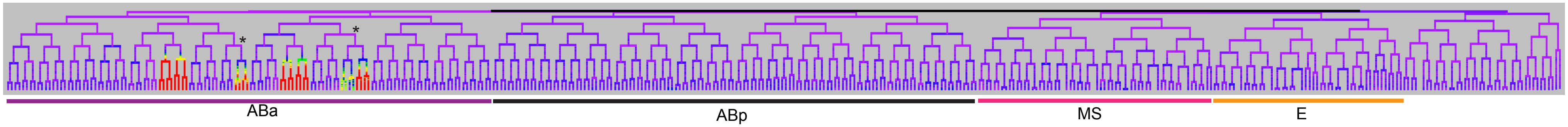

D **CEH-36** Expressed in ABa, ABp, MS; Strongly activated by *pop-1/sys-1* in ABa, strongly repressed by *pop-1* in ABp, unregulated in MS

Wildtype

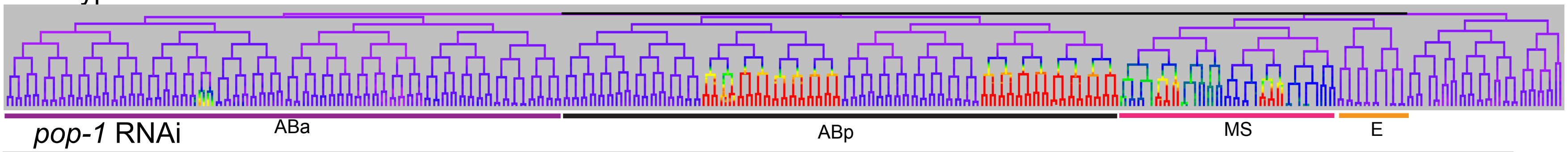

pop-1 RNAi

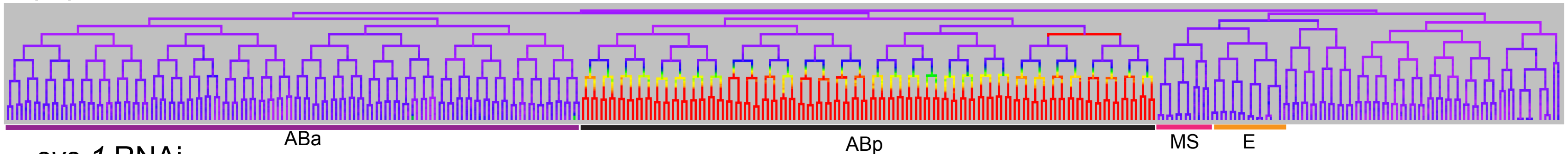

sys-1 RNAi

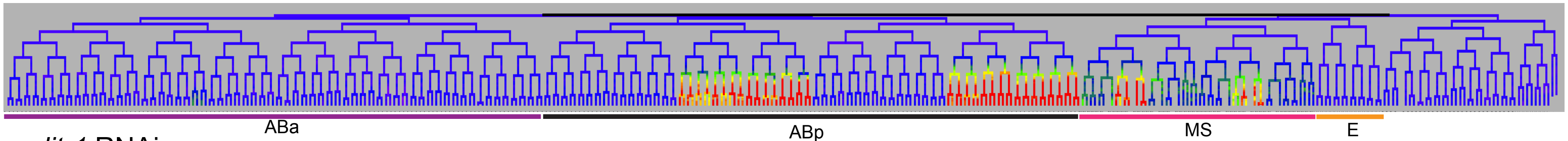

lit-1 RNAi

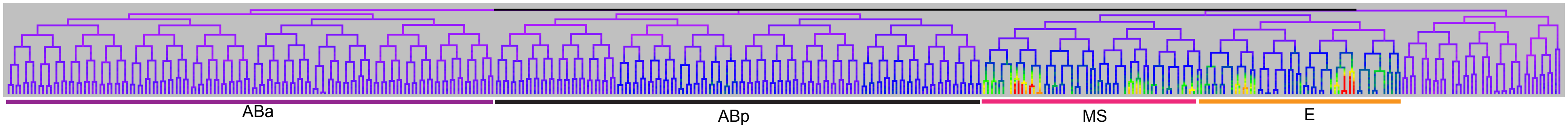

Figure S11E

CEH-43 Expressed in ABa, ABp; Indirectly regulated by *pop-1*

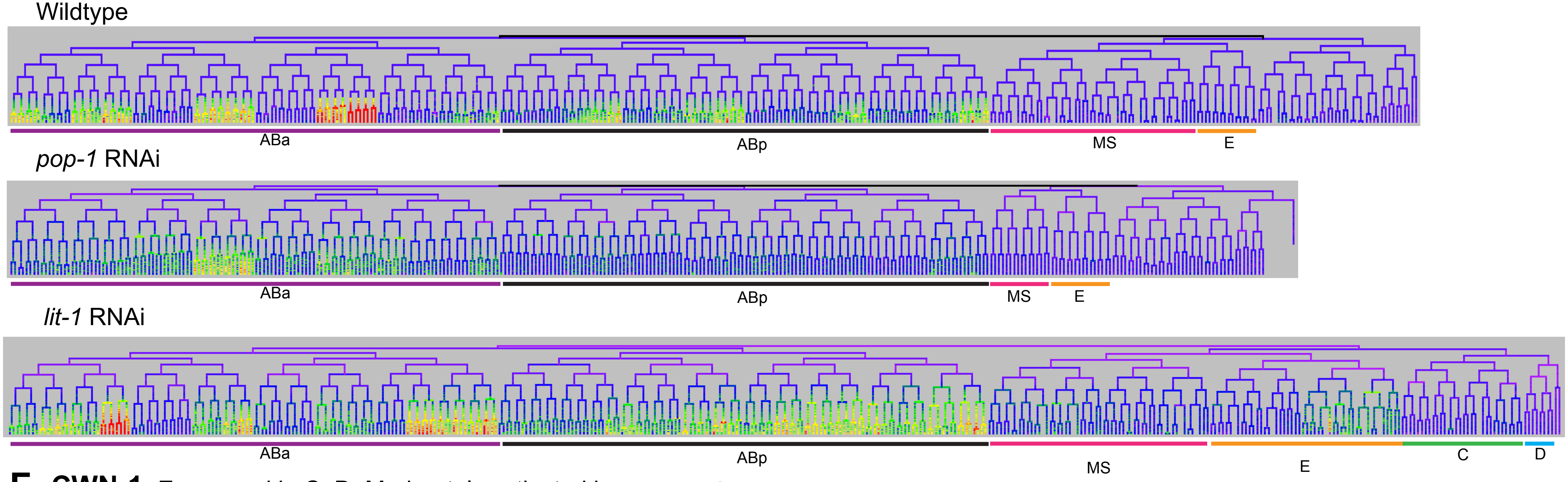

F **CWN-1** Expressed in C, D; Moderately activated by *pop-1* in C, D

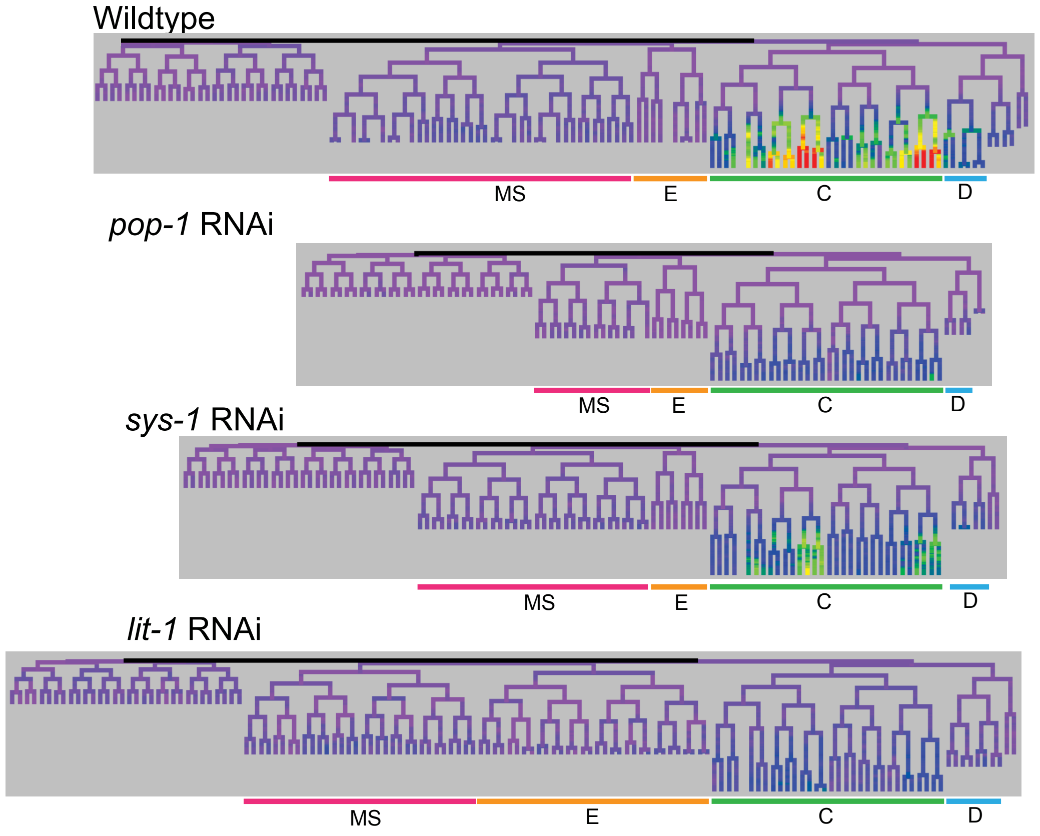

Figure S11G

CWN-2 Expressed in ABa, ABp, E; Weakly activated by *pop-1/sys-1* in ABa and E, weakly repressed by *pop-1* in ABp

Wildtype

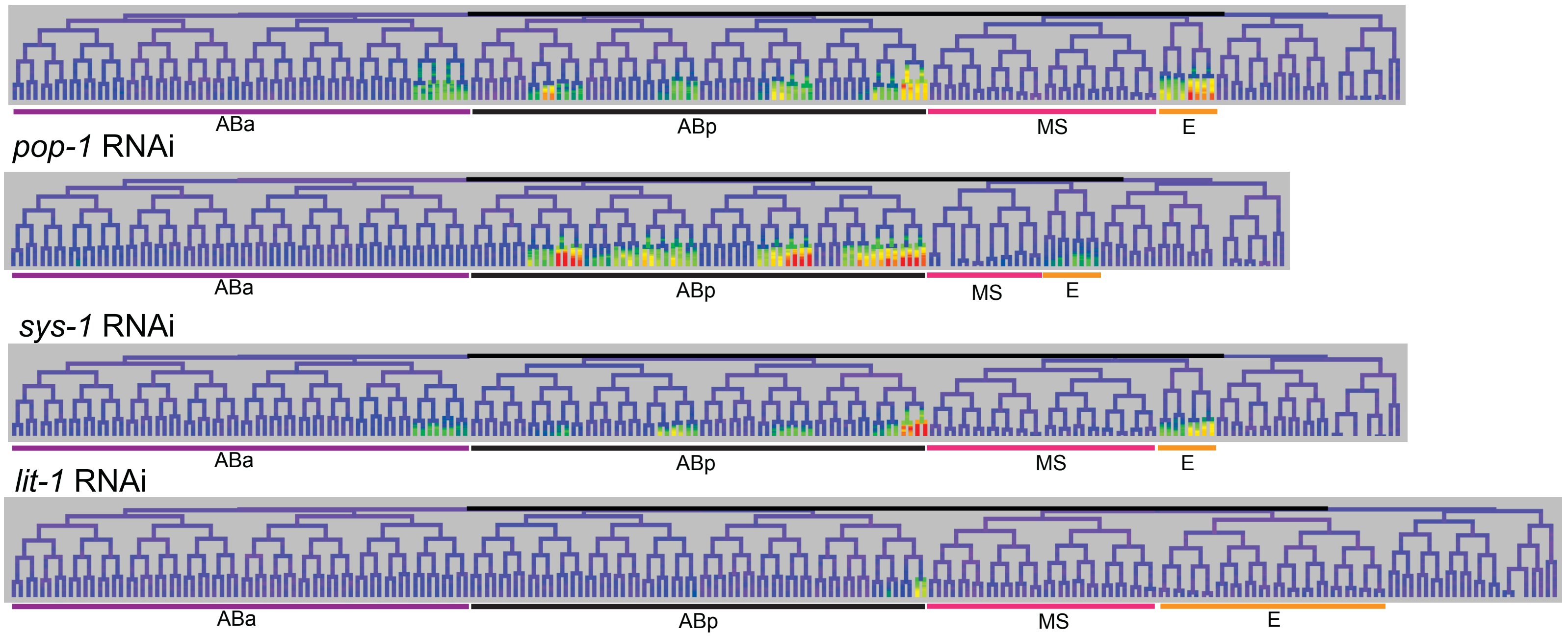

H **ELT-6** Expressed in ABa, ABp, MS, C, D; Moderately activated by *pop-1/sys-1* in ABa, ABp, MS, C, D

Wildtype

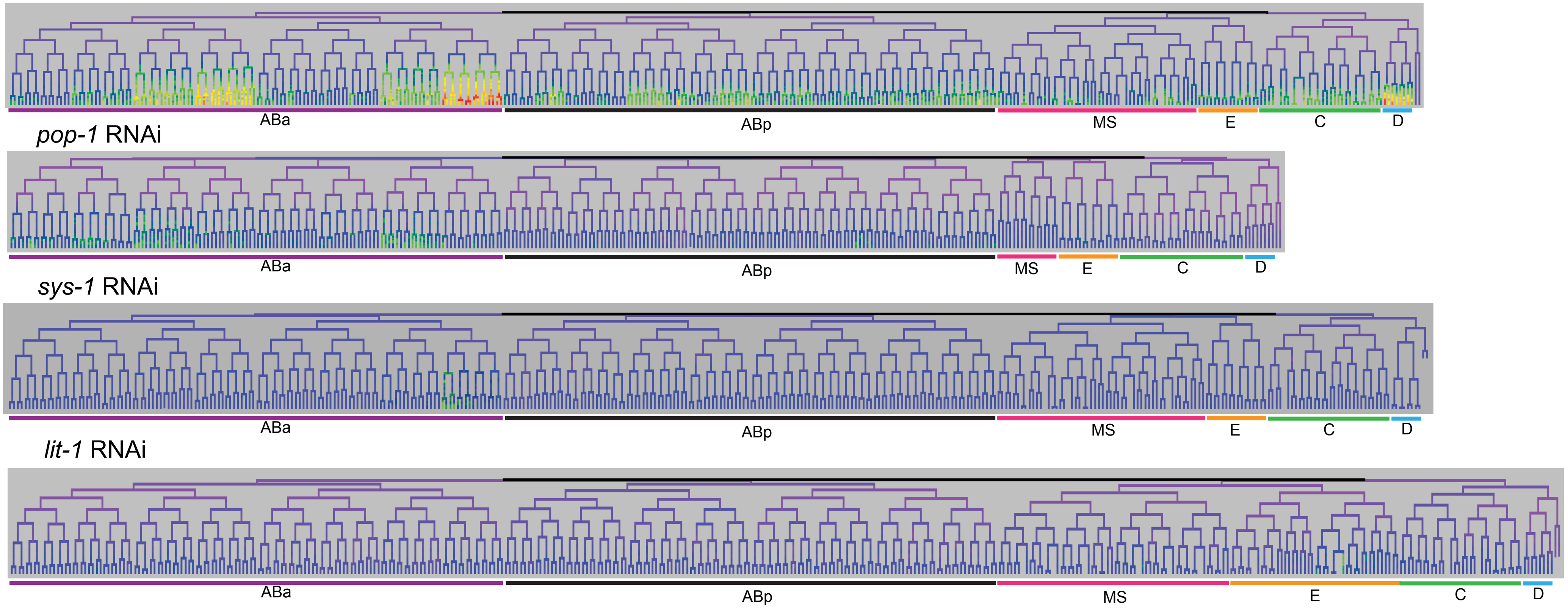

Figure S11I

END-1 Expressed in E; Activated by *pop-1* in E, repressed by *pop-1* in MS

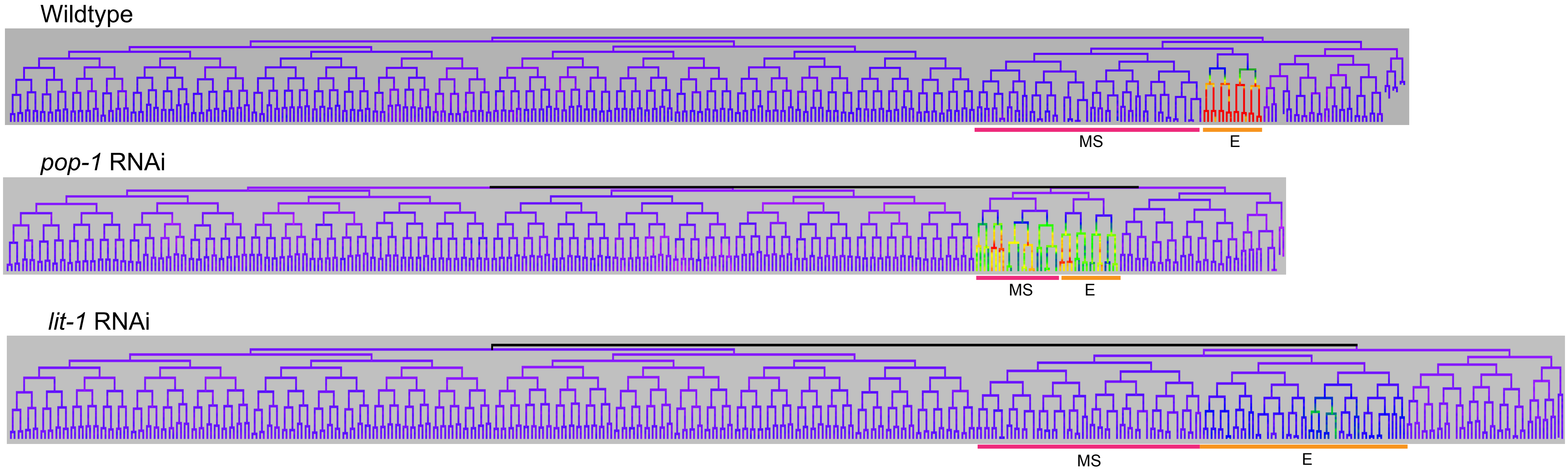

J

END-3 Expressed in E; Activated by *pop-1* in E, repressed by *pop-1* in MS

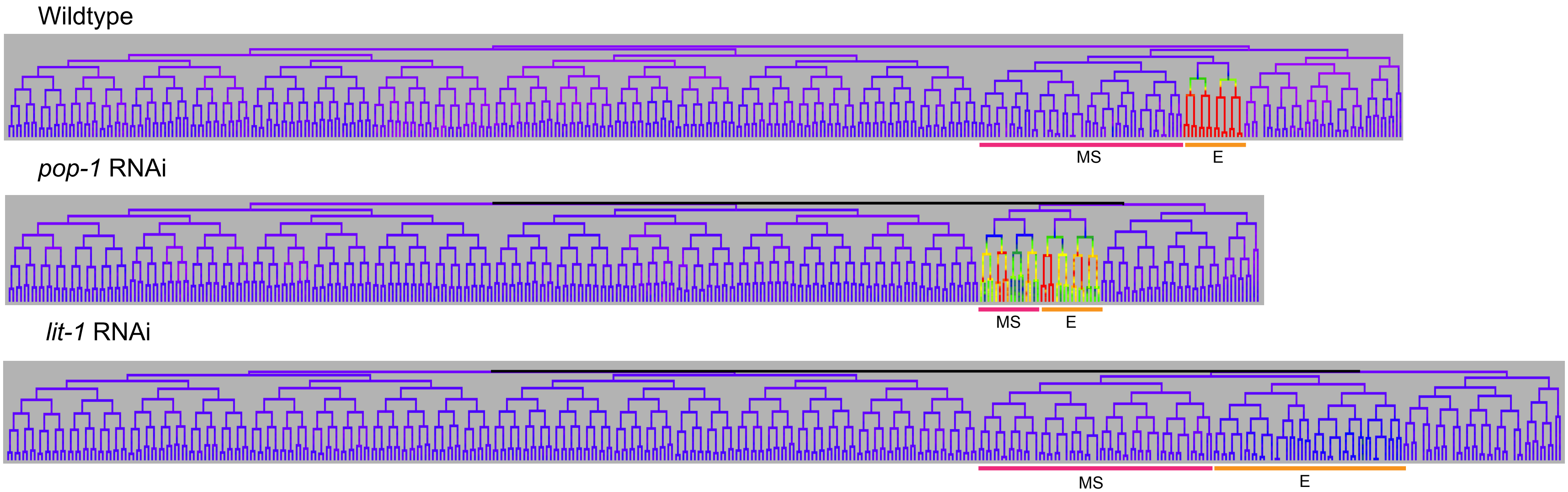

Figure S11K

ETS-7 Expressed in ABa, ABp, E, C; Moderately activated by *pop-1/sys-1* in ABa, ABp, E, C

Wildtype

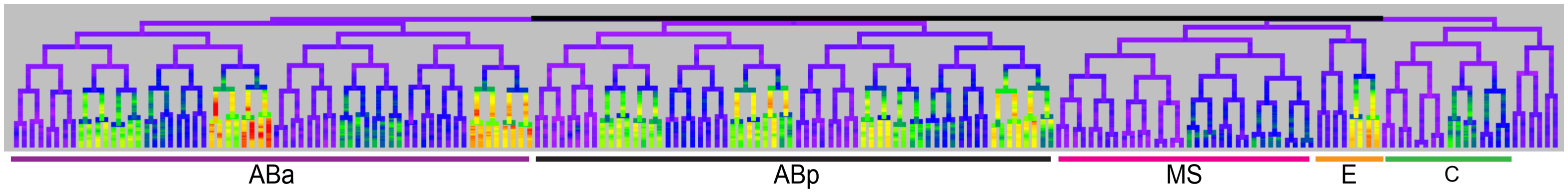

pop-1 RNAi

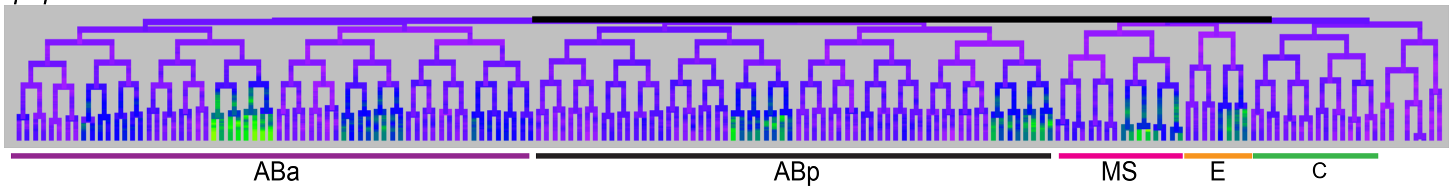

sys-1 RNAi

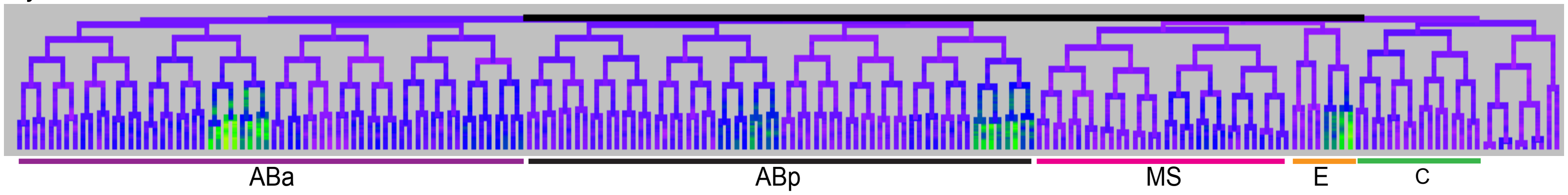

lit-1 RNAi

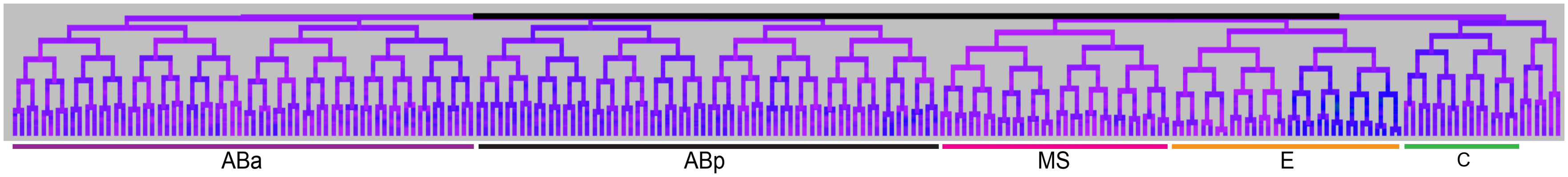

L

MIR-57 Expressed in ABp, C; Weakly activated by *pop-1/sys-1* in ABp and C

Wildtype

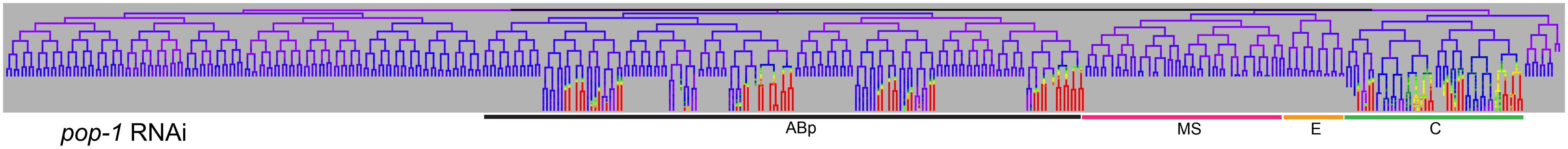

pop-1 RNAi

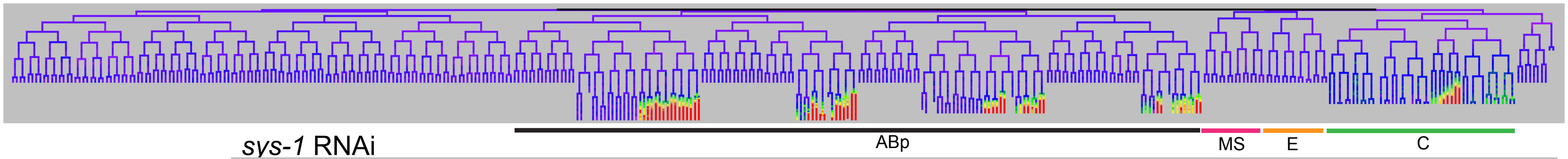

sys-1 RNAi

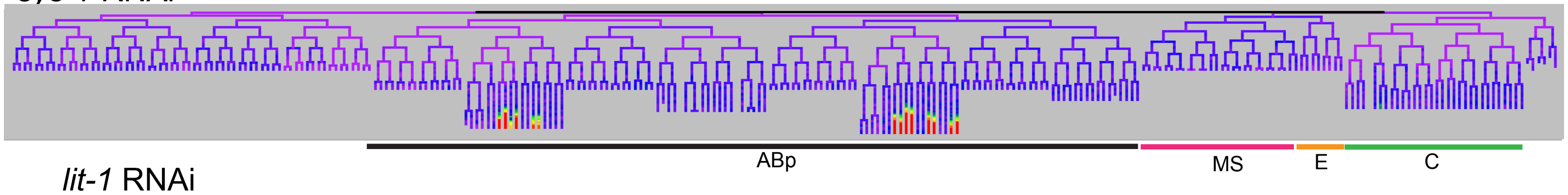

lit-1 RNAi

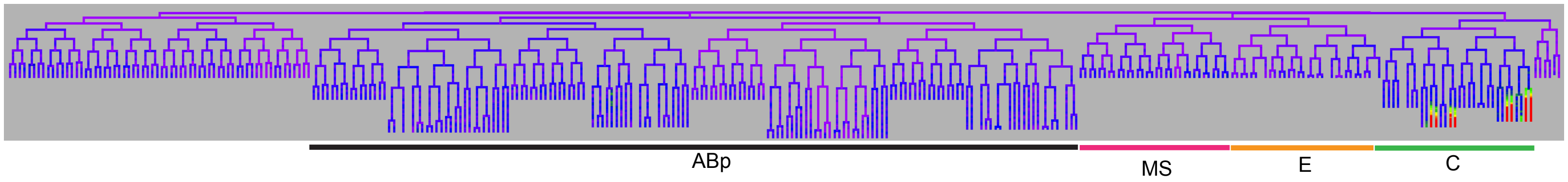

Figure S11

M **MOM-2** Unregulated by *pop-1*

Wildtype

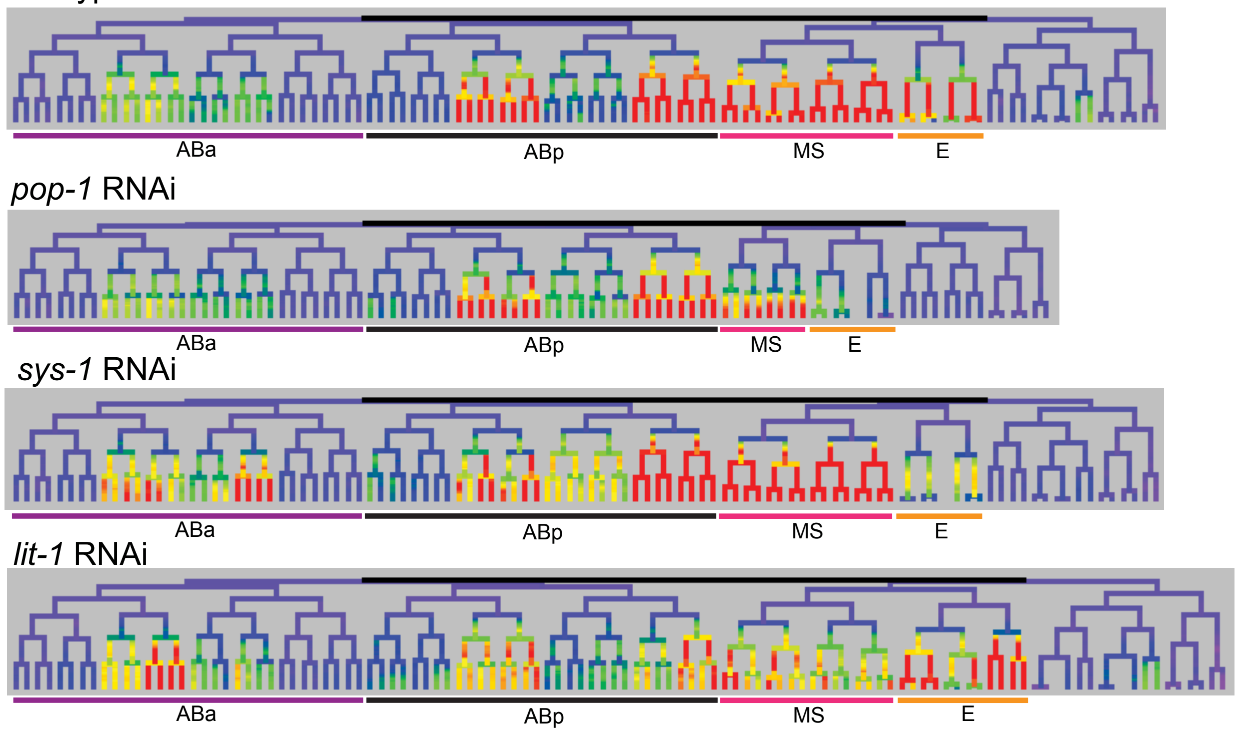

N **NHR-25** Expressed in ABa, ABp, C; Weakly activated by *pop-1/sys-1* in ABa, moderately activated in ABp, unregulated in C

Wildtype

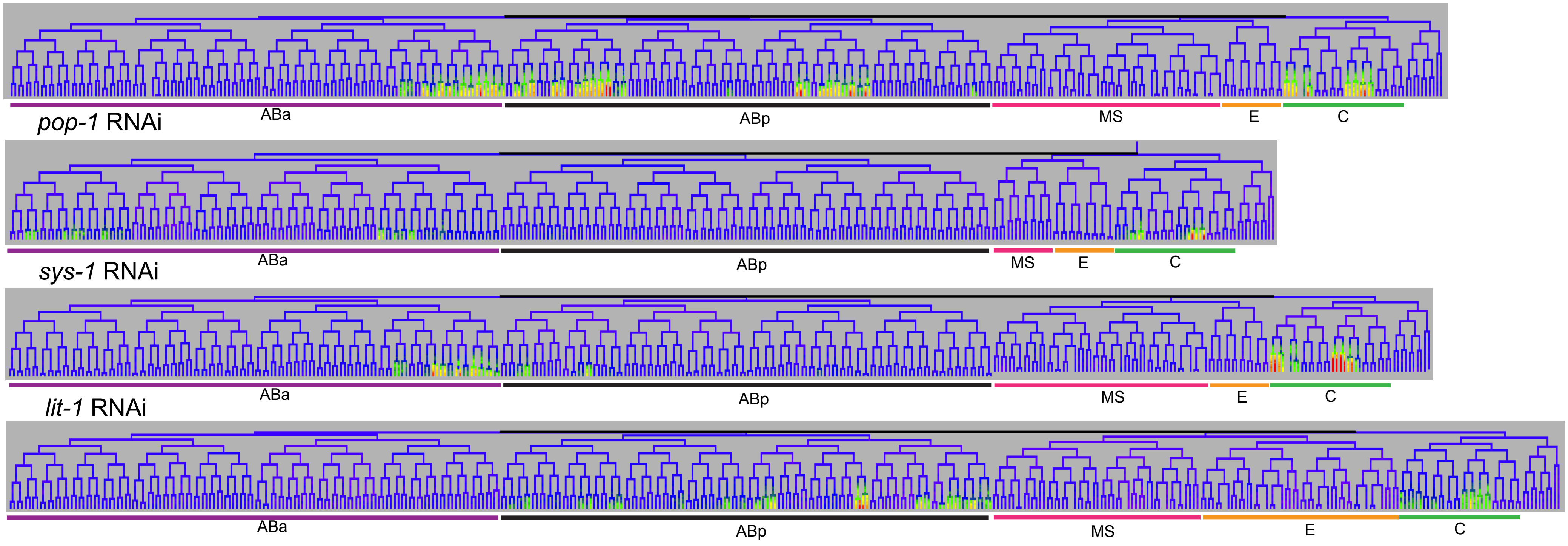

Figure S11 O

NHR-67 Expressed in ABp, MS; Weakly activated by *pop-1/sys-1* in ABp, moderately activated in MS

Wildtype

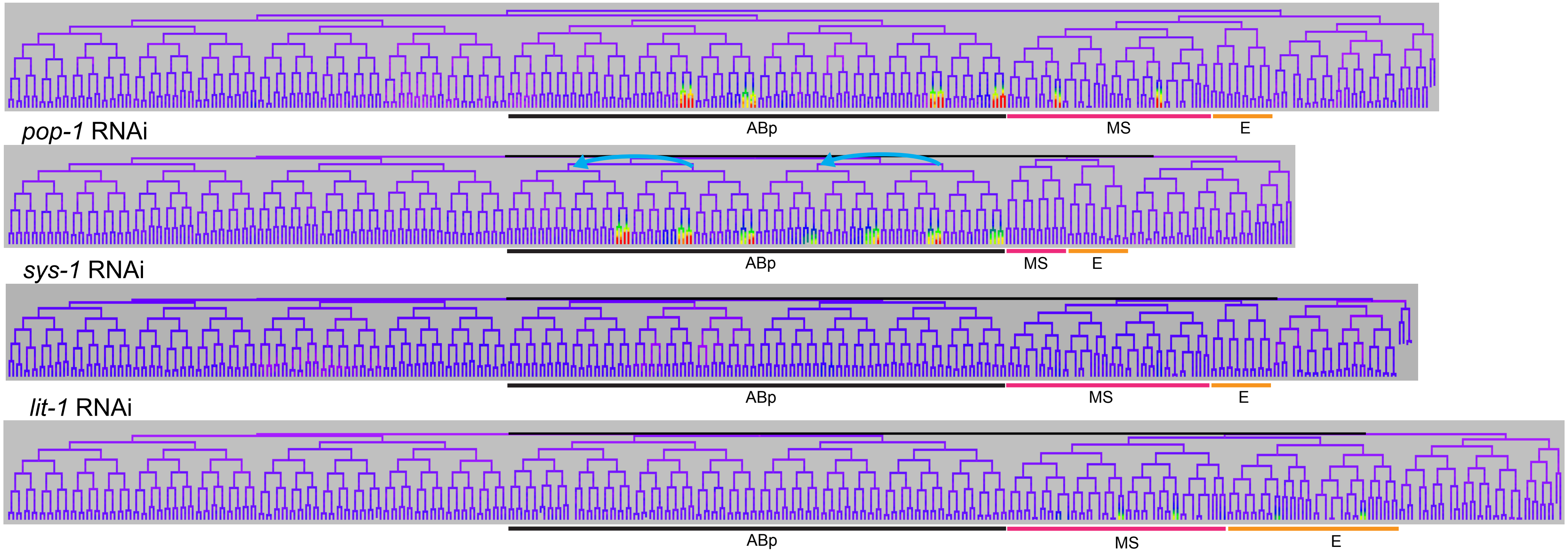

P **NOB-1** Expressed in ABp, E, C; Strongly activated by *pop-1/sys-1* in ABp, C, unregulated in E

Wildtype

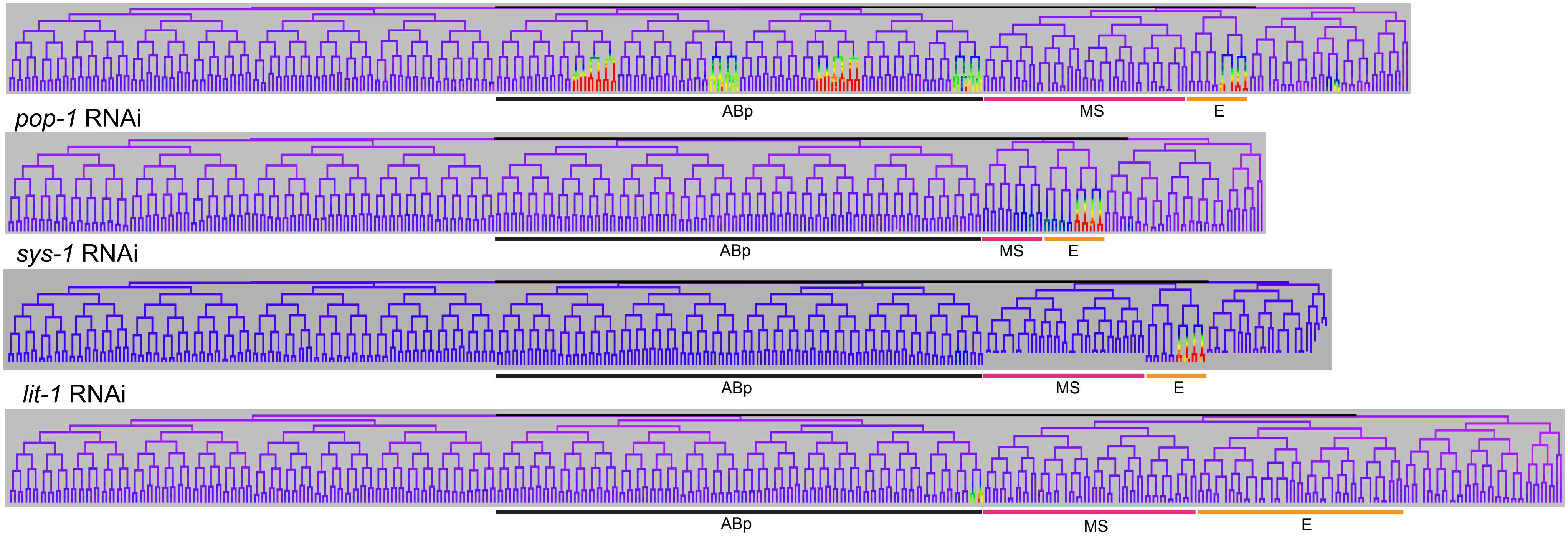

Figure S11Q

PAL-1 Expressed in ABa, ABp, C, D; Moderately activated by *pop-1/sys-1* in ABa, ABp, weakly activated in D, dual regulated by *pop-1* in C

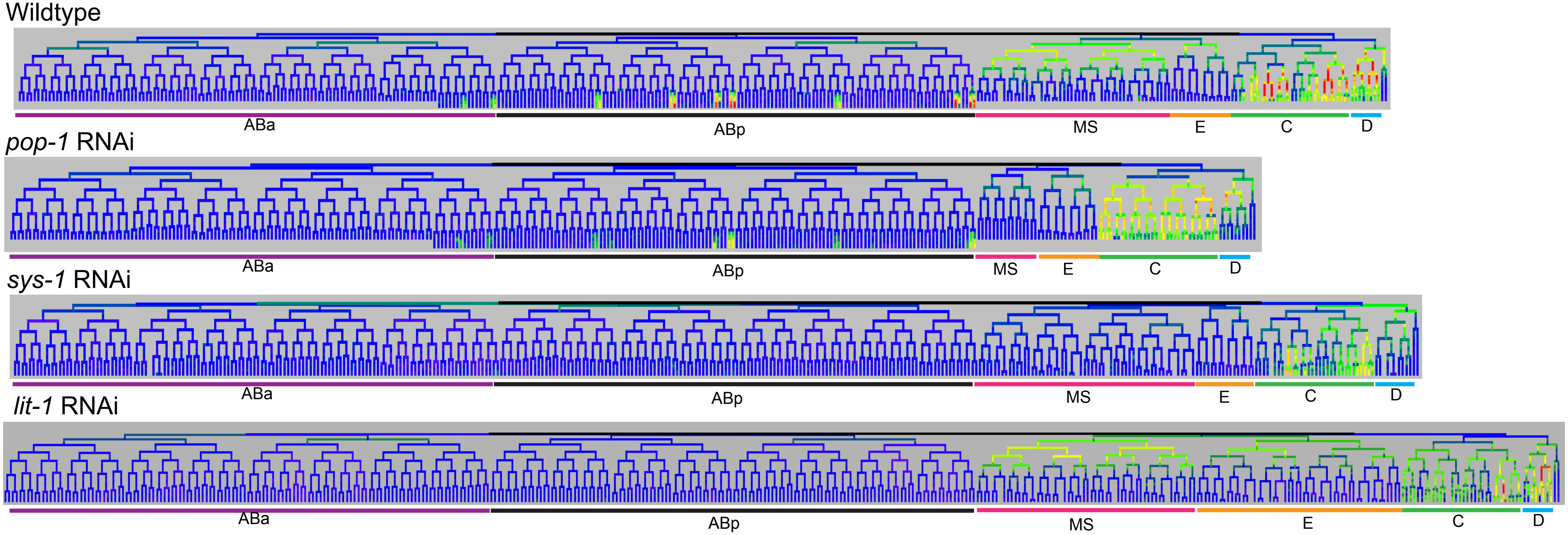

R **PAX-3** Expressed in ABp; Moderately activated by *pop-1/sys-1* in ABp

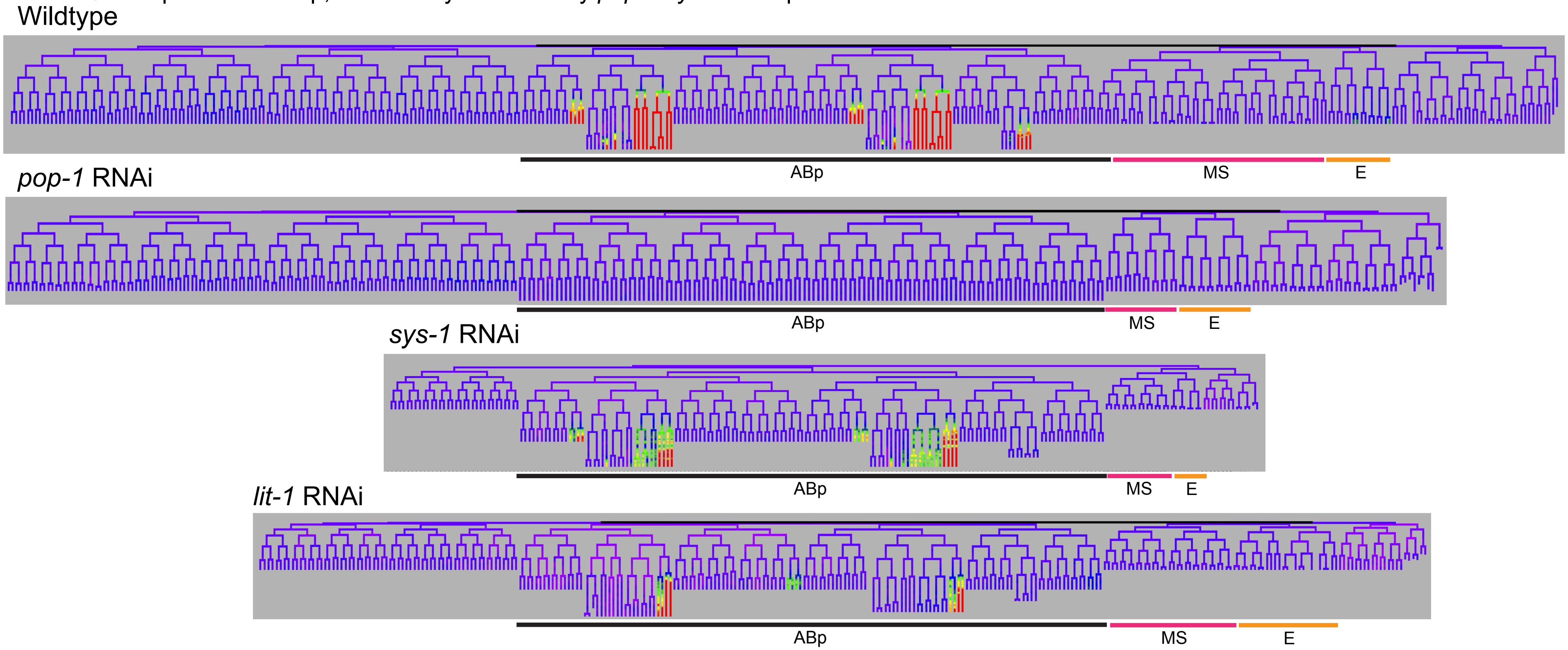

Figure S11S

SEM-2 Expressed in ABa, ABp, MS, E; Strongly repressed by *pop-1* in ABa,ABp, unregulated in MS, E

Wildtype

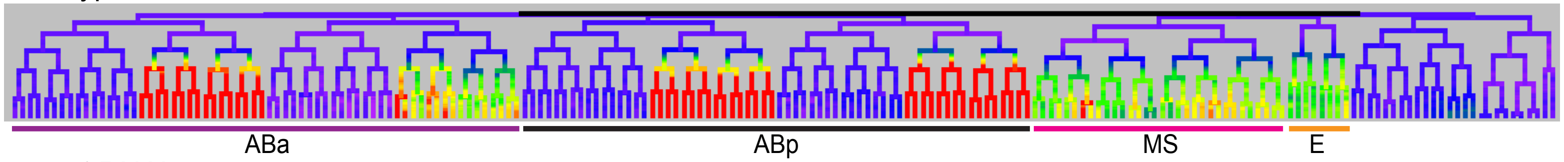

pop-1 RNAi

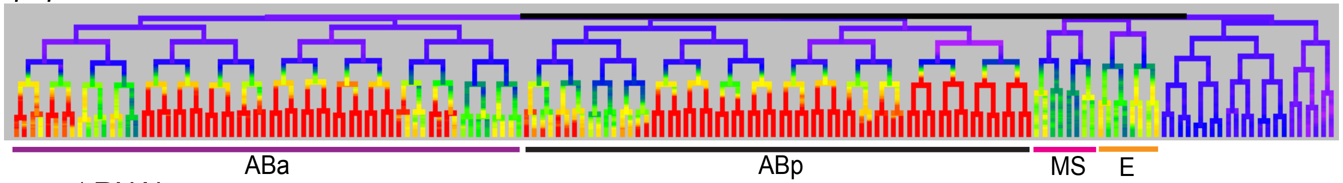

sys-1 RNAi

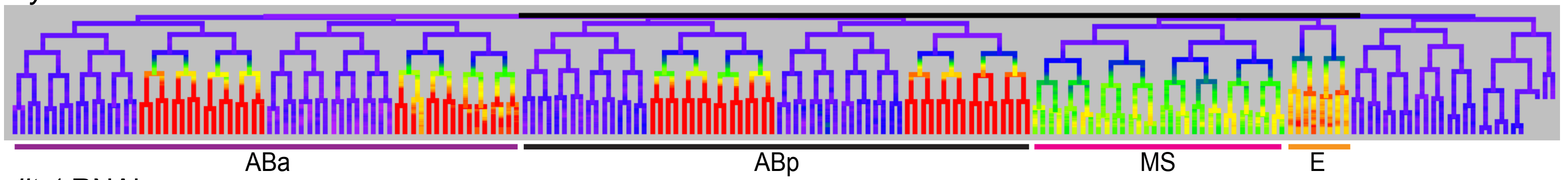

lit-1 RNAi

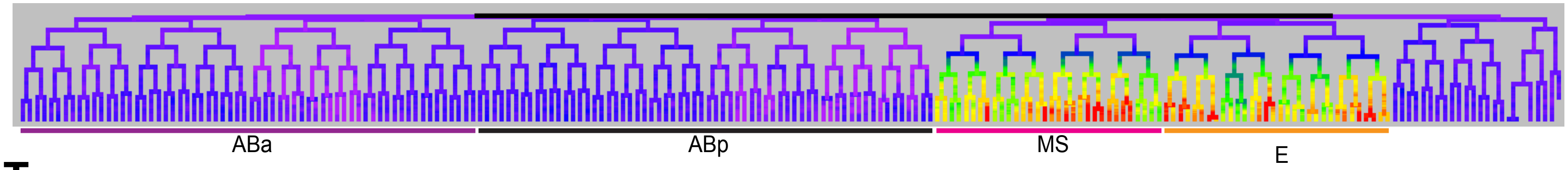

T

TBX-11 Expressed in ABa, ABp, MS, E, C; Moderately repressed by *pop-1* in ABa, MS, E, C, D, unregulated in ABp

Wildtype

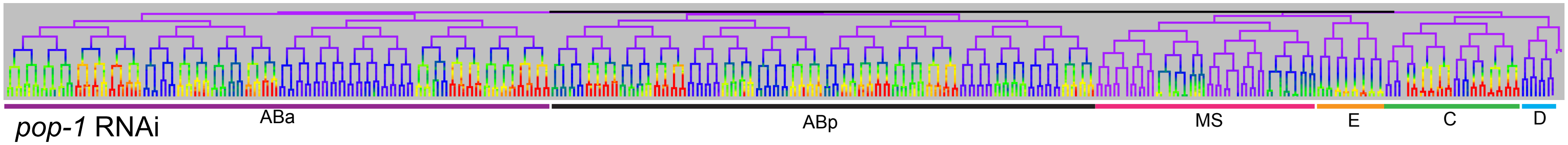

pop-1 RNAi

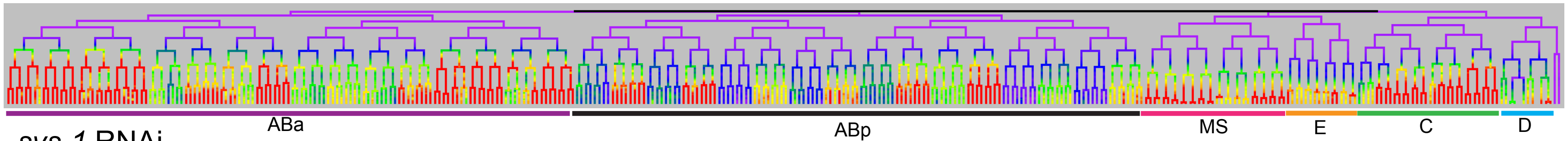

sys-1 RNAi

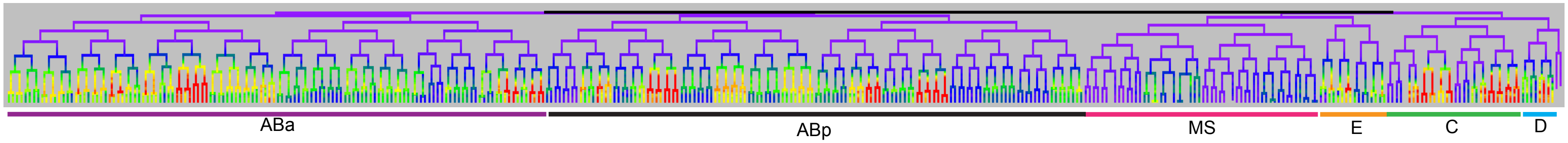

lit-1 RNAi

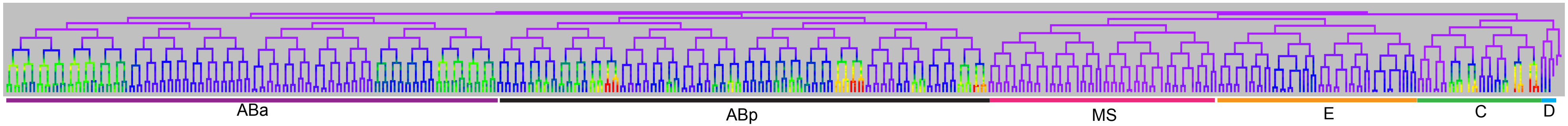

Figure S11U

TLP-1 Expressed in ABa, ABp, E, C; Strongly repressed by *pop-1* in ABa, ABp, unregulated in E, C

Wildtype

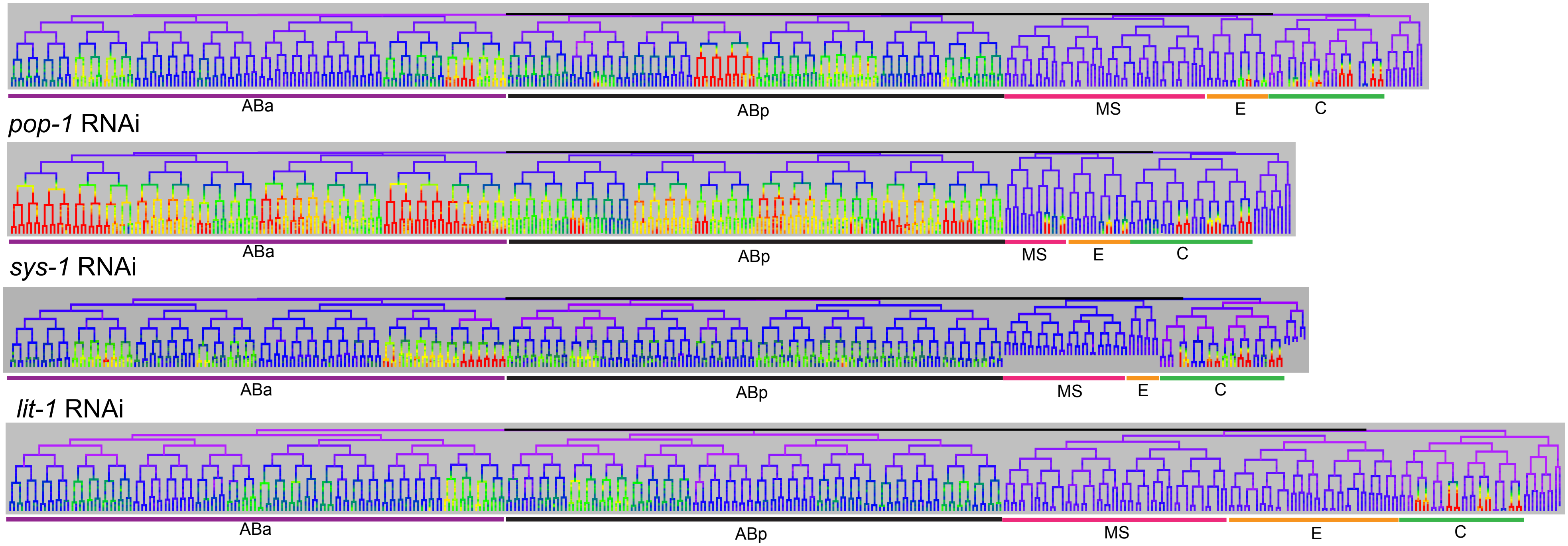

V **UNC-30** Expressed in ABp; Strongly repressed by *pop-1* in ABp

Wildtype

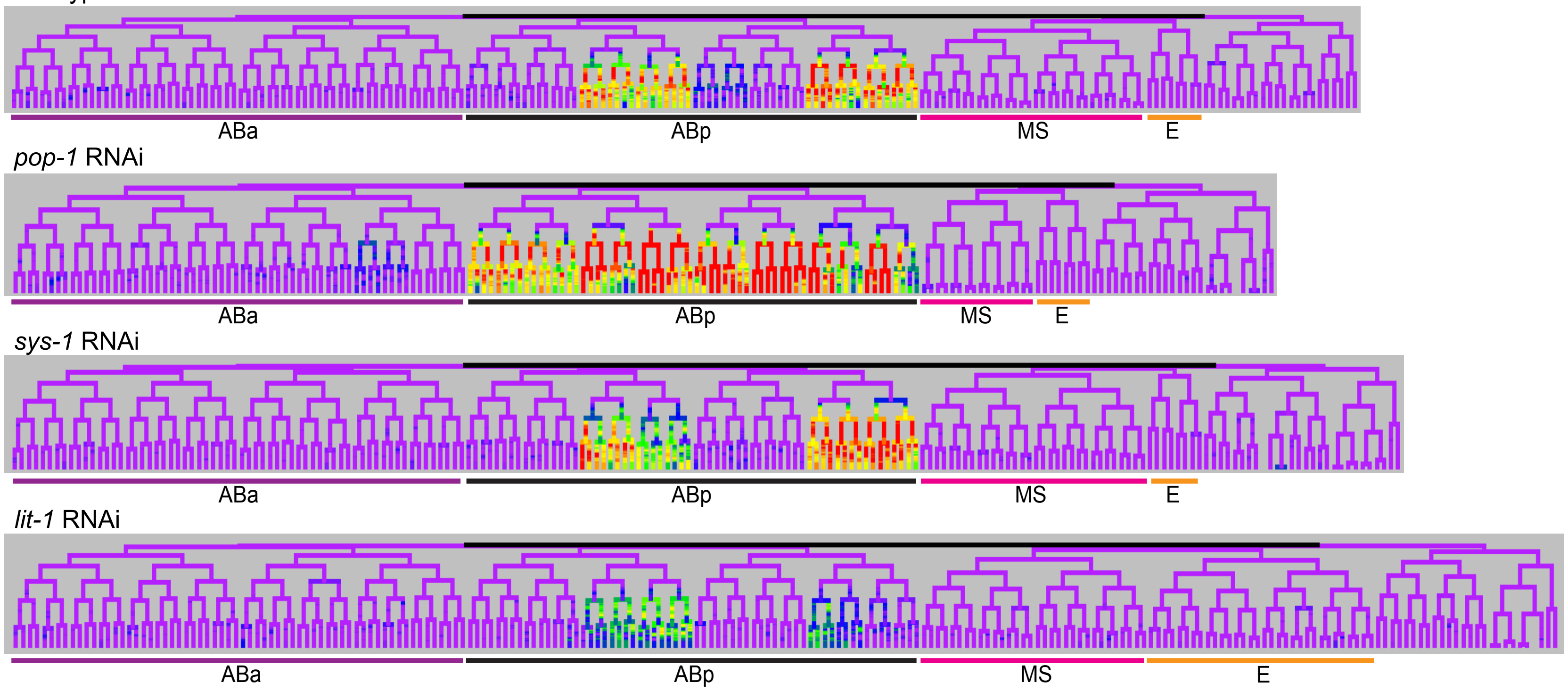

Figure S11W

UNC-130 Expressed in ABp; Weakly activated by *pop-1/sys-1* in ABp

Wildtype

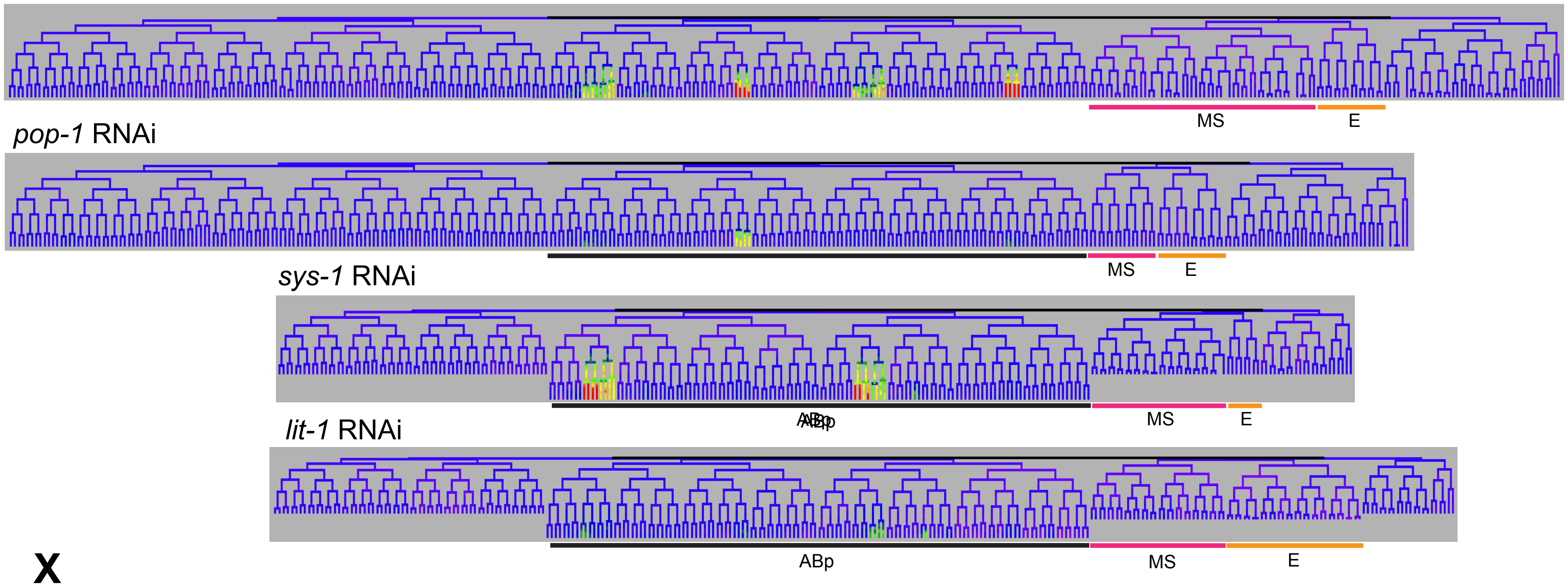

X

VAB-7 Expressed in C; Moderately activated by *pop-1/sys-1* in C

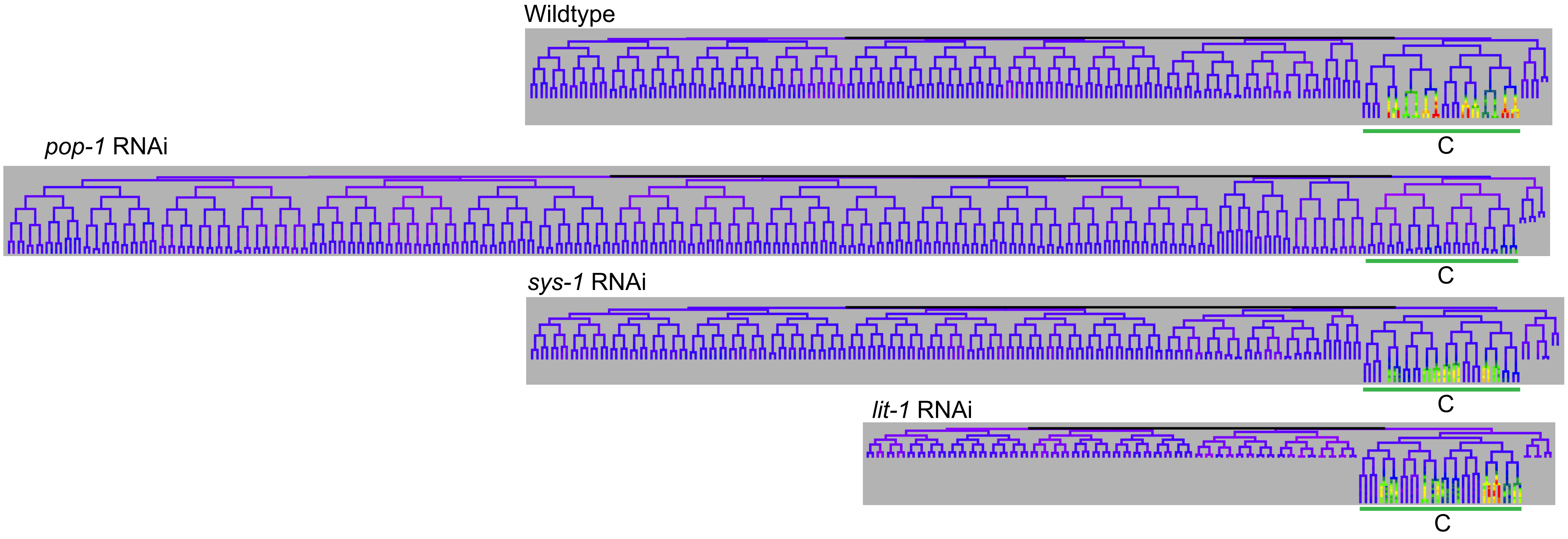

Supplement: S11 Fig — The MS (pink) and E (orange) lineages are labeled and underlined for all genes to highlight the MS—>E fate transformation with pop-1 RNAi and the E—>MS fate transformation with lit-1 RNAi. Additional expressing lineages are also labeled and underlined. Expression was considered to be strongly activated by pop-1/sys-1 if expression was completely lost with both pop-1 and sys-1 RNAi; moderately activated if completely lost with one and reduced in the other, and weakly activated if expression was reduced or delayed with pop-1 and sys-1 RNAi. Expression was considered to be strongly repressed if pop-1 RNAi caused broadly expanded expression at roughly the same level as wild type, moderately repressed if pop-1 RNAi caused broadly expanded expression at a level less than wildtype and weakly repressed if expression was narrowly expanded at a level less than wildtype. No change was considered unregulated. A) Expression of CEH-6::GFP is lost or reduced in most expressing lineages after pop-1, sys-1 or lit-1 RNAi. B) Expression of CEH-13::GFP is expanded in ABalap (white asterisk) after pop-1 RNAi because of a known fate transformation in which ABala adopts the fate of ABarp (white asterisk)[14]. Since expression in ABarpp is largely unchanged in both pop-1 and sys-1 RNAi, we conclude that ceh-13 is unregulated by pop-1/sys-1 in ABa (purple underline). Expression in ABp (black underline) is significantly decreased with pop-1, sys-1 and lit-1 RNAi. Note that the broad, transient early expression is more affected than the later stronger expression limited to a few branches. C) Expression of the ceh-27 promoter is expanded in ABa with lit-1 RNAi, which is inconsistent with direct regulation. Expression of the ceh-27 promoter is expanded in ABp with pop-1 RNAi, and ABp expression is largely unchanged with sys-1 RNAi and lost with lit-1 RNAi. D) Expression of the ceh-36 promoter is lost with pop-1, sys-1 and lit-1 RNAi, while broad expansion is observed in ABp with pop-1 RNAi and [file pgen.1005585.s016.pdf]
